# Molecular characterization of vaginal microbiota using a new 22-species qRT-PCR test to achieve a relative-abundance and species-based diagnosis of bacterial vaginosis

**DOI:** 10.3389/fcimb.2024.1409774

**Published:** 2024-06-28

**Authors:** Ayodeji B. Oyenihi, Ronald Haines, Jason Trama, Sebastian Faro, Eli Mordechai, Martin E. Adelson, John Osei Sekyere

**Affiliations:** ^1^ Institute for Biomarker Research, Medical Diagnostic Laboratories, Genesis Biotechnology Group, Hamilton, NJ, United States; ^2^ Memorial Women’s Care, Houston, TX, United States

**Keywords:** bacterial vaginosis (BV), *MDL-BV index*, vaginitis, qRT-PCR, BVAB, vaginal microbiome, machine learning

## Abstract

**Background:**

Numerous bacteria are involved in the etiology of bacterial vaginosis (BV). Yet, current tests only focus on a select few. We therefore designed a new test targeting 22 BV-relevant species.

**Methods:**

Using 946 stored vaginal samples, a new qPCR test that quantitatively identifies 22 bacterial species was designed. The distribution and relative abundance of each species, α- and β-diversities, correlation, and species co-existence were determined per sample. A diagnostic index was modeled from the data, trained, and tested to classify samples into BV-positive, BV-negative, or transitional BV.

**Results:**

The qPCR test identified all 22 targeted species with 95 – 100% sensitivity and specificity within 8 hours (from sample reception). Across most samples, *Lactobacillus iners, Lactobacillus crispatus, Lactobacillus jensenii, Gardnerella vaginalis, Fannyhessea (Atopobium) vaginae, Prevotella bivia*, and *Megasphaera* sp. type 1 were relatively abundant. BVAB-1 was more abundant and distributed than BVAB-2 and BVAB-3. No *Mycoplasma genitalium* was found. The inter-sample similarity was very low, and correlations existed between key species, which were used to model, train, and test a diagnostic index: *MDL-BV index*. The *MDL-BV index*, using both species and relative abundance markers, classified samples into three vaginal microbiome states. Testing this index on our samples, 491 were BV-positive, 318 were BV-negative, and 137 were transitional BV. Although important differences in BV status were observed between different age groups, races, and pregnancy status, they were statistically insignificant.

**Conclusion:**

Using a diverse and large number of vaginal samples from different races and age groups, including pregnant women, the new qRT-PCR test and *MDL-BV index* efficiently diagnosed BV within 8 hours (from sample reception), using 22 BV-associated species.

## 1 Introduction

Bacterial Vaginosis (BV), a condition in which the normal *Lactobacillus*-rich vaginal microbiome becomes dominated by polymicrobial anaerobic bacterial species under non-acidic pH, remains the most common cause of abnormal vaginal discharge in reproductive-age women, with an estimated prevalence rate of 29% in North America (Abou Chacra et al., 2021; Elnaggar et al., 2023). The normal vaginal microbiome is dominated by three major *Lactobacillus* species, *L. crispatus, L. jensenii*, and *L. gasseri*, with low abundance of *L. acidophilus*, which protects the vagina by producing lactic acid, hydrogen peroxide, and bacteriocins that suppress bacterial growth (Pacha-Herrera et al., 2022; Wu et al., 2022). *Lactobacillus iners*, however, is quite enigmatic as it occurs in both healthy and unhealthy vaginal environments; *L. iners*, unlike *L. crispatus, L. jensenii*, and *L. gasseri*, produces the human non-functional L form of lactic acid (Basavaprabhu et al., 2020).

To produce lactic acid, which is necessary to maintain an acidic pH of < 4.5, *Lactobacillus* sp. uses glycogen deposited unto the vaginal walls from the epithelial cells (Shen et al., 2022; Navarro et al., 2023). The acidic pH keeps away most microbial species and allows *Lactobacillus* sp. to proliferate, keeping the species diversity in the normal vaginal microbiota low. Lactic acid is metabolized from glycogen, whose production and deposition are mediated by circulating estrogen in a dose-dependent manner. In the absence of glycogen or lactic-acid–producing species, the pH rises, allowing other microbes to proliferate, colonize the vagina, and increase the microbiota diversity. Hence, physiological factors disrupting or increasing estrogen levels indirectly affect *Lactobacillus* sp. and BV-associated bacterial species’ prevalence (Shen et al., 2022; Navarro et al., 2023). The etiology and pathogenesis of BV are still not fully understood, making it important to study the involved microbes for timely diagnosis and treatment. Particularly, the interactions between species within the BV microbiome that cause pathologies, treatment failure and recurrence, or healing need further interrogations to enable a species biomarker-based diagnosis of BV.

The colonization of the vaginal microbiota by anaerobic bacterial and fungal species causes BV or vulvovaginal candidiasis, respectively, characterized by increased vaginal discharge with a fishy odor (Swidsinski et al., 2023). Dysbiosis also predisposes the vagina to sequelae of other sexually transmitted infections (STIs) and obstetric disorders. Although BV is asymptomatic in many women, it is associated with the development of common obstetric and gynecologic complications (Javed et al., 2019; Cheng et al., 2020; Gustin et al., 2021). It also increases the risk of contracting HIV and other STIs and pelvic inflammatory disease (PID) (Unemo et al., 2017). Notably, BV recurrence after treatment is common (estimated to affect 50% of women annually) (Hilbert et al., 2016; Coudray and Madhivanan, 2020; Coudray et al., 2020) and may be due to re-infections from sexual partners or failure of current treatment options (Coudray et al., 2020; Vodstrcil et al., 2021). Besides the health impacts of recurrence, BV treatment costs are increasing annually, specifically in the USA: this economic impact is particularly pronounced in BV-associated preterm births and other obstetric complications (Peebles et al., 2019; Watkins et al., 2024).

Species such as *Gardnerella vaginalis, Fannyhessea vaginae, Prevotella bivia, Megasphaera* sp., and BVAB are commonly implicated in the etiology of BV, with *G. vaginalis* and *F. vaginae* being common in most BV infections (Swidsinski et al., 2023) as sessiles. Swidsinksi et al. (2023) observed that vaginal epithelial cells from females with BV were covered with *G. vaginalis* biofilms that encapsulated other species, forming a polymicrobial biofilm, as well as by non-adherent planktonic bacterial cells. The biofilms associated with BV explain their ability to escape both the immune response and antimicrobial chemotherapy as biofilms prevent the immune cells and antimicrobial agents from reaching the pathogens (Cangui-Panchi et al., 2023; Swidsinski et al., 2023). This leads to high treatment failure and recurrence rates of about 50% (Muñoz-Barreno et al., 2021); indeed, the ability of these biofilm-coated polymicrobial species to spread into the uterus and fallopian tube explains their resistance to antimicrobials and the immune response (Swidsinski et al., 2023).

To diagnose BV, clinicians rely mostly on the classical clinical signs and symptoms outlined in Amsel’s criteria (Amsel et al., 1983) or on the microscopically based Nugent score (Nugent et al., 1991). While these standard diagnostic methods have been effective over the years, they have been confronted with limitations such as being labor-intensive and time-consuming, subjective and unable to accurately identify pathogens (Muzny et al., 2023). As recently discussed by Muzny et al. (2023), nucleic acid tests ease the burden of laboriously going through the clinical testing criteria, saving clinicians time and effort for other duties. The development of culture-independent molecular diagnostics has enabled the detection of non-cultivable bacterial species associated with BV and continues to revolutionize infectious disease diagnosis (Hilbert et al., 2016; van den Munckhof et al., 2019; Bhujel et al., 2021). Molecular tests such as real-time polymerase chain reaction (RT-PCR), combined with the traditional clinical diagnostic criteria, have greatly improved the sensitivity and specificity of detecting BV pathogens (Hilbert et al., 2016; van den Munckhof et al., 2019; Bhujel et al., 2021). They are also applicable in monitoring patient response to antibiotic therapy (Onderdonk et al., 2016; Elnaggar et al., 2023). This approach has proven more useful for identifying patients at risk for recurrent BV (Jones, 2019; Coudray and Madhivanan, 2020; Vodstrcil et al., 2021; Wu et al., 2022; Sobel and Vempati, 2024; Watkins et al., 2024).

Besides next-generation sequencing (NGS)-based metagenomics that target all genomes, current PCR diagnostics for BV mainly focus on a smaller spectrum of bacterial species. Hence, to increase the resolution and diagnostic power of PCR-based BV diagnostics (Balashov et al., 2014), we designed a new 22-species quantitative Real-Time PCR assay (qRT-PCR) that quantitatively detects 22 vaginal bacterial species within 8 hours (from sample reception). This newly designed proprietary qRT-PCR assay (Bacterial Vaginosis (with *Lactobacillus* Profiling) Panel^®^, Medical Diagnostic Laboratories, L.L.C. (MDL), New Jersey, USA) was used to screen 946 vaginal samples routinely obtained from different health centers across the United States. We further used the results, with assistance from machine-learning algorithms (Decision Trees and Random Forests) to design, train, and test an index (herein termed the *MDL-BV index*) that used a relative-abundance and species-based markers to classify the vaginal microbiome in three categories: BV-negative (healthy microbiome), transitional BV (between healthy and unhealthy vaginal microbiome), and BV-positive (abnormal microbiome).

## 2 Materials and methods

### 2.1 Specimen collection and processing

Clinical vaginal samples are routinely obtained from different healthcare centers across the United States for diagnostic processing at MDL. Historical vaginal specimens (n = 946) marked for disposal were received in *One*Swab^®^ (Copan Diagnostics, CA, USA) or *Thin*Prep^®^ (Hologic, MA, USA) transport media in a Clinical Laboratory Improvement Amendments (CLIA)-certified infectious disease laboratory facility between January and June 2023 and stored at −80°C were selected randomly for this study. This included specimens from symptomatic, asymptomatic, pregnant, or non-pregnant females and from whom vaginal profiling was requested. For each biological specimen that arrives at the laboratory facility, specimen accessioning occurs that assigns a random MDL number to ensure the de-identification of specimens. To further the de-identification of specimens during this study, samples that matched our collection criteria were randomized and the MDL numbers associated with each sample were not recorded.

### 2.2 Targeted bacterial species

The newly designed quantitative real-time PCR (qRT-PCR) assay (Bacterial Vaginosis (with *Lactobacillus* Profiling) Panel^®^) is a BV diagnostic assay designed by MDL to qualitatively and quantitatively detect 22 bacterial species that are found in the eubiotic and dysbiotic vaginal microbiome. These species are *L. crispatus, L. jensenii, L. gasseri, L. iners, L. acidophilus, Gardnerella vaginalis, Fannyhessea vaginae* (*Atopobium vaginae*), *Megasphaera* sp. types 1 and 2, *Prevotella bivia*, Bacterial Vaginosis-Associated Bacterium (BVAB) 1–3, *Ureaplasma urealyticum, Mycoplasma hominis, Mycoplasma genitalium, Mobiluncus curtisii, Mobiluncus mulieris, Sneathia sanguinegens, Bifidobacterium breve, Bacteroides fragilis*, and *Streptococcus anginosus* (Onderdonk et al., 2016; Muzny et al., 2020; Mondal et al., 2023; Powell et al., 2023).

### 2.3 DNA preparation

DNA from the vaginal specimens was extracted according to validated in-house laboratory protocols using QIAamp^®^ DNA Mini Kit (QIAGEN, Maryland, USA) and the X-tractor Gene^®^ DNA workstation (QIAGEN, Maryland, USA) with slight modifications. Canine Herpes Virus DNA was spiked into the samples and subsequently detected as an internal extraction control.

To serve as amplification standards, plasmids for each of the 22 bacterial species were generated by whole-gene synthesis (Genewiz^®^, Azenta Life Sciences, Waltham, USA). Briefly, a species-specific region of each species was amplified, synthesized, and cloned into the pUC-GW-AMP plasmid vector with an ampicillin-resistance marker. These plasmids were transformed into chemically competent *Escherichia coli* cells (One Shot™ TOP10, ThermoFisher Scientific, New Jersey, USA) and grown in liquid Lysogeny Broth (LB) containing 50 µg/mL Ampicillin. Plasmid DNA was isolated from overnight cultures using Wizard^®^ Plus SV Minipreps DNA Purification Systems (Promega, Wisconsin, USA) according to the manufacturer’s protocol. Extracted DNA was subsequently quantified spectrophotometrically using NanoDrop 1000 equipment (ThermoFisher Scientific, New Jersey, USA). Standard 10-fold serial dilutions of 10^8^ to 10^1^ DNA copies/µL were prepared for each bacterial species.

### 2.4 Identification of bacterial species by multiplex qRT-PCR

Each vaginal and plasmid control DNA sample was amplified in triplicates using species-specific primers and fluorescent probes on a CFX384 Touch Real-Time PCR Detection System (Bio-Rad, California, USA). The specific primer and probe sequences, as well as the multiplex RT-PCR conditions in the Bacterial Vaginosis (with *Lactobacillus* Profiling) Panel by Real-Time PCR assay are proprietary to MDL. An aliquot of 0.5μL of DNA was used as the template in a 4μL total PCR reaction mixture containing four primer sets, four probes, and the in-house—prepared mastermix of Taq DNA polymerase and dNTPs (MDL, New Jersey, USA) in multiplex PCR reactions. Human β-globulin DNA served as the internal control while no-template control was included to account for potential extraneous nucleic acid contamination. After amplification, a standard curve (fluorescence vs cycle number) was generated for each species and the target DNA copy for each sample was extrapolated. A 10^2^ copies/μL concentration in each sample was established as the positive cutoff for a given bacterium. The relative percentage concentration of all the bacterial species identified in each vaginal specimen was then computed and tabulated (**Supplementary Table S1**).

### 2.5 Relative abundance- and species-based classification of BV

The mean, median, standard deviation, and concentration distribution of each species across samples as well as alpha-diversity, Shannon index, beta-diversity (Bray-Curtis Dissimilarity matrix), and relative abundance were calculated from the gDNA concentration data (obtained from the qRT-PCR amplification results of each species from the samples) and represented as box and Whisker plots, histograms, heatmaps, and charts. Using the distribution patterns in the data, a relative abundance or concentration-based cut-off criteria was established and used to define BV-positive (abnormal vaginal microbiota with bacterial vaginosis and depleted *Lactobacillus* sp.: ≤ 40% relative abundance), BV-negative (i.e., normal vaginal microbiota with ≥ 70% *Lactobacillus* sp. relative abundance), and transitional BV (transition between negative and positive BV microbiota with 30–50% *Lactobacillus* sp. relative abundance) status (see **Table 1** for full definitions). Using the species-species correlation and co-existence data as well as previous research from literature, a species-based criteria was also established for determining BV-positive, BV-negative, and transitional BV status. A two-tier system was then created by synthesizing the species-based and concentration-based BV diagnosis criteria, which were used to interpret the PCR results. This criterion was called the *MDL-BV index*, which was further trained and tested on the qRT-PCR and demographics data using machine-learning algorithms (Decision Trees and Random Forests) in Python. The datasets were divided into two: one for training and one for testing. Python libraries such as numpy, pandas, scikitlearn, XGBoost, and matplotlib, were used for analysis and visualization.

**Table 1:** *MDL-BV index* designed to interpret the new 22 species qRT-PCR results and diagnose vaginal samples as BV-positive, BV-negative, or transitional BV.

BV Status^#^	Relative abundance (%)			
	*Lactobacillus* sp.	BVAB (1, -2, -3), *S. anginosus, B. fragilis*	*G. vaginalis, P. bivia, A. vaginalis, Megasphaera* sp. *1*	Megasphaera sp. 2, *U. urealyticum, Mycoplasma* sp.*, Mobiluncus* sp.*, S. sanguinegens, B. breve*
**Healthy microbiome (BV negative)**	≥ 70	≤3	≤ 20	≤ 20
**Transitional BV**	30 – 60	0 - 10	≤ 30	≤ 50
**BV positive**	≤ 40	0 - 100	20 – 100	0 – 100

# (1) When a sample falls within both BV-negative and transitional BV, assign it to transitional BV. (2) When a sample falls within both BV-positive and transitional BV, assign it to BV-positive. (3) When at least three of a sample’s four groups/biomarkers’ relative abundances fall within a single BV state and only one falls in another BV state, override the single disagreement, and assign or classify the sample to the BV state with which the three relative abundances agree or fall into. (4) When biomarkers 2 (BVAB group) and 4 (Megasphaera sp. 2 group) have a relative abundance of zero, then a relative abundance of 0 – 30% for Group 1 and 70 – 100% for Group 3 is BV-positive; a relative abundance of 30 – 50% (Group 1) is transitional BV; and 60–100% (Group 1) is BV negative. (5) When every group is zero and only group 4 is 100%, define it as BV positive. (6) When all groups are zero, filter out that sample as there are no results to report

### 2.6 Demographics

The age, race, and pregnancy status of the females from whom the samples were taken were collected and used to stratify the data to compare the different rates, distributions, and relative abundance of the various species, BV-positive, BV-negative, and transitional BV according to age, race, and pregnancy status.

### 2.7 Statistical analysis

Statistical analyses were performed with Prism version 10.1.2 (Graph-Pad, California USA) or the R statistical package. Quantitative data obtained in this study were expressed as the mean ± standard error of the mean (SEM). To detect bacterial associations and correlations, Pearson’s correlation coefficient was chosen. The Kruskal–Wallis test was employed to determine any statistically significant differences in age distributions among BV classification groups. The data were parsed through Python (and Biopython) to calculate the significance of the coexistence between any two species using Chi-square and T-test.

Three types of groupings were used in this (Chi-square and T-test) analysis: (1) all *Lactobacillus* species were grouped into one and their absence/presence was compared with the absence/presence of each non-*Lactobacillus* species; (2) all *Lactobacillus* species except *L. iners* were grouped into one and their absence/presence was compared with the absence/presence of each non-*Lactobacillus* species; (3) the presence/absence of each *Lactobacillus* species was compared with that of each non-*Lactobacillus* species. The resulting data were tabulated in **Supplementary Table S2**, and the significant results were filtered out and used to generate heat maps. The results were only considered significant if *P* < 0.05.

## 3 Results

### 3.1 Demographics

**Figure 1:**
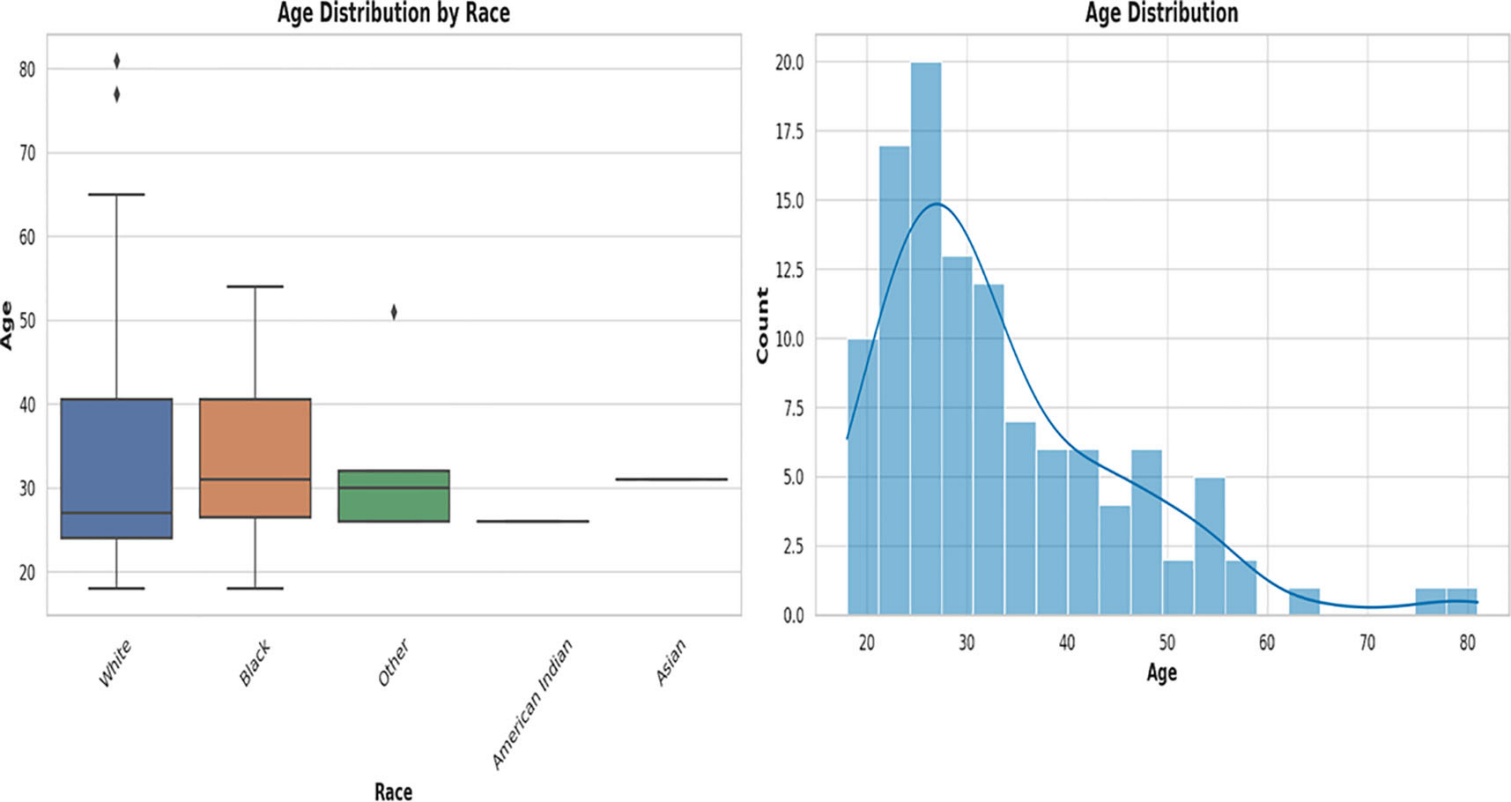
Box plot and histogram showing the age distribution among the races from which the vaginal samples were obtained. The box plot on the left, showing the age distribution among White people, Black people, Other (races), Native American or Alaska Natives, and Asians. White people were the largest population, followed by Black people. Other races are those who belong to none of the four races. The median ages among the races were very close within 28 and 35. An age distribution histogram on the right, shows the overall age distribution of participants in the study. The histogram includes a kernel density estimate (KDE) to show the smooth distribution of ages. The data is somewhat right-skewed, indicating a younger population with fewer older women. Most women fell into the 20–40 age range, with a peak around the late 20s to early 30s.

The vaginal samples were obtained from 946 women whose average age was 34.8 years, with a standard deviation of 13.3 years. The ages ranged from 18 to 84 years. The median age was 32 years; hence, the age distribution is somewhat skewed towards younger women [**Figure 1**; **Supplementary Table S1** (Demographics)]. Fifty-three women reported as pregnant while the rest were either not pregnant or unknown. Race data was obtained for 245 samples: White (n = 145 samples), Black (n = 79), “Other race” (n = 15), Asian (n = 4), and Native American (n = 2) [**Figure 1**; **Supplementary Table S1** (Demographics)]. The “Other race” category refers to those not falling within any of the four races above: Hispanic/Latino, Native Hawaiian or Pacific Islander, and mixed races.

The age distribution of the races [**Figure 1**; **Supplementary Table S1** (Demographics)] shows a broader distribution of ages within the White population (with outliers) than that of the other races, albeit the median ages across all races fell within a narrow range of 28 – 35 years. The age distribution of White people ranged from 18 – 83 years, with 50% falling within 28 – 40 years. Whereas the ages of Black people ranged from 18 to 55 years, 50% were within the same 28 – 40 years range. The “Other races”, which includes Hispanics/Latinos, had ages between 28 and 32 years and the median age was different among all the races [**Figure 1**; **Supplementary Table S1** (Demographics)].

### 3.2 Identification and distribution of species across samples

**Figure 2:**
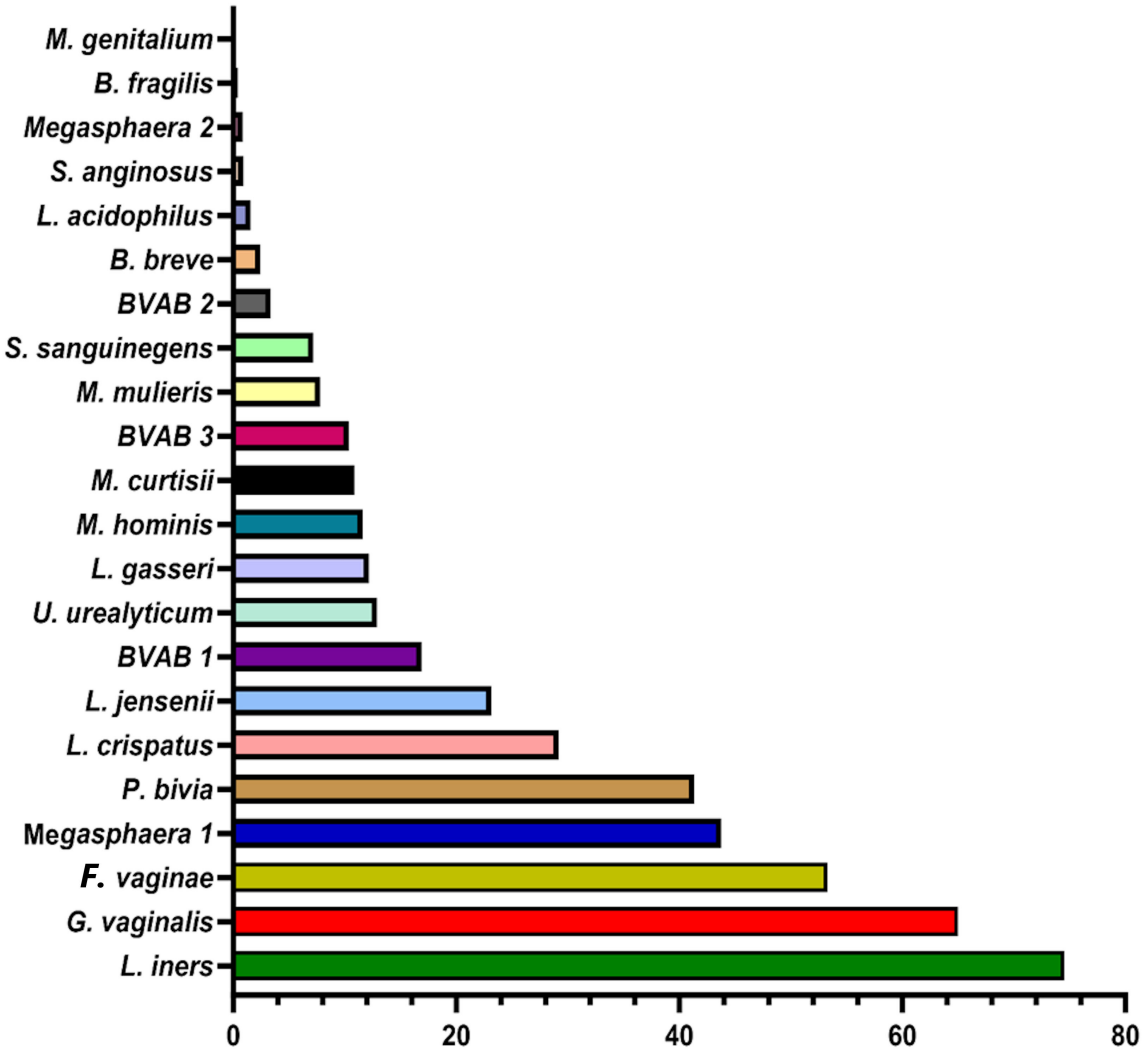
Percentage of the count of each species across all vaginal specimens (n = 946) as detected by qPCR. Among the 946 samples, it is observed that the most dominant species are *Lactobacillus iners, Gardnerella vaginalis, Atopobium (Fannyhessea) vaginae, Megasphaera* sp. type 1*, Prevotella bivia, Lactobacillus crispatus, and Lactobacillus jensenii.* The horizontal axis is the percentage of each species across all samples. No *Mycoplasma genitalium* was detected in any of all the samples.

The BV qRT-PCR assay efficiently identified the targeted 22 species with 95 – 100% sensitivity and specificity. Except for *M. genitalium* which was not detected in any sample, all the other species were present in at least one of the 946 samples. The percentage of the count of the identified species across all the samples are as follows: *L. iners* (75%), *G. vaginalis* (65%), *F. vaginae* (53%), *Megasphaera* sp. type 1 (44%), and *P. bivia* (41%) (**Figure 2**). These are followed by *L. crispatus* (29%), *L. jensenii* (23%), BVAB-1 (17%), *U. urealyticum* (13%), *L. gasseri* (12%), *M. hominis* (12%), *M. curtisii* (11%), and BVAB-3 (10%). The remaining species had less than 10% relative abundance across all samples combined (**Figure 2**).

To understand the distribution (spread) of the species and their concentrations across the samples, we used bar charts, histograms, and box and Whisker plots. *L. iners*, followed by *G. vaginalis, F. vaginae, Megasphaera* sp. type 1*, P. bivia, and L. crispatus* had better concentration distributions across the samples than the other species, with their median concentrations (10^2^ and 10^6^ gDNA copies/µL) being found in more than 100 samples. Indeed, other species were found in low concentrations across all the samples (**Supplementary Figures S1**–**S22**). The relative concentration for each identified species ranged between 10^2^ and 10^8^ genomic DNA copies/µL and the median range was between 10^2^ and 10^6^ copies/µL (**Figure 3**).

**Figure 3:**
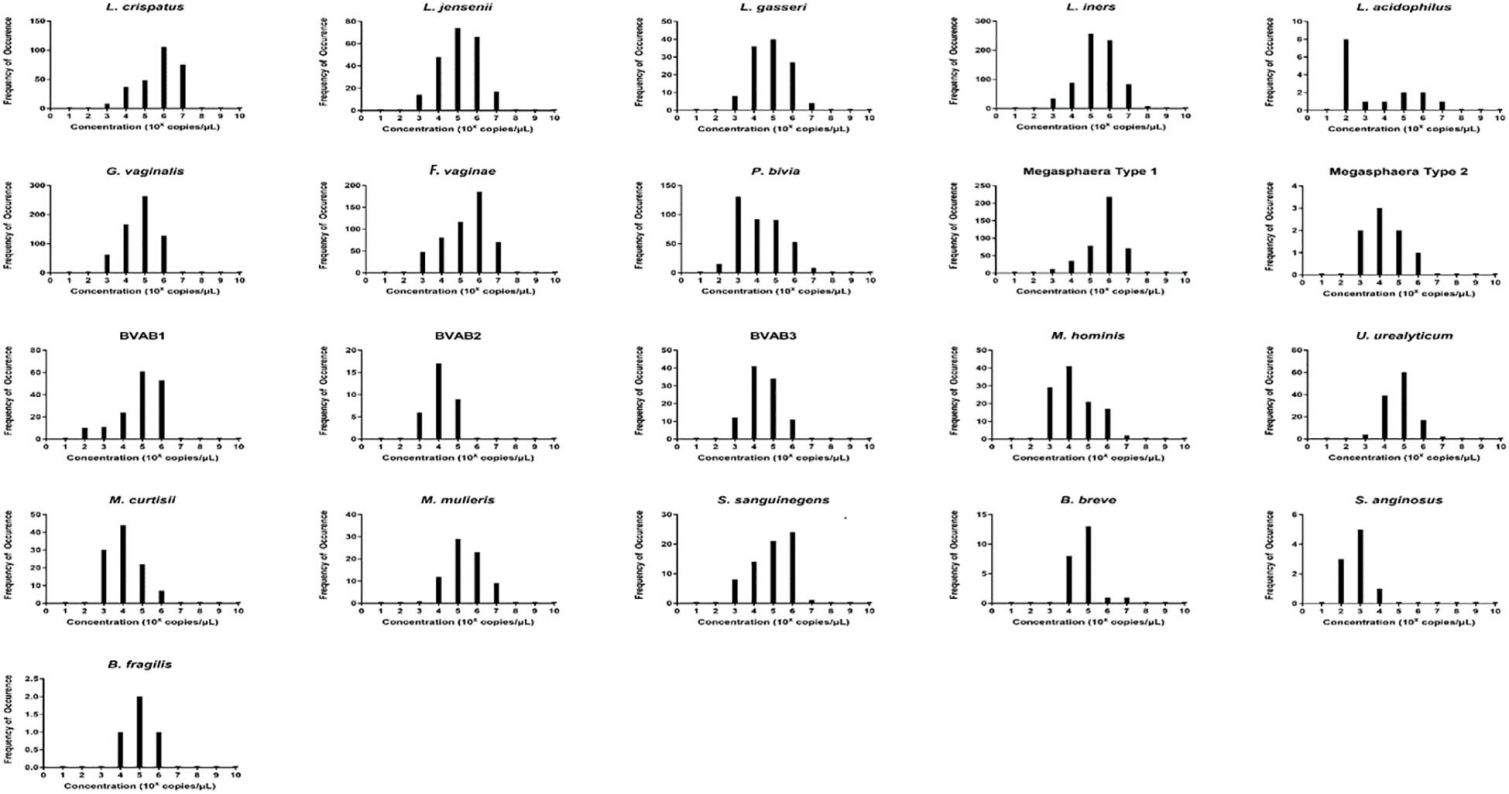
The relative concentration distribution of the identified bacterial species in vaginal swabs (n = 946). Each chart represents the concentration distribution per species; *M. genitalium* is not shown as was not detected in any of the samples. The vertical axis shows the count (frequency) of samples while the concentrations are shown on the horizontal axis. The median concentrations (10^2^ and 10^6^ gDNA copies/µL) of *Lactobacillus iners* and *L. crispatus, Gardnerella vaginalis, Atopobium (Fannyhessea) vaginae, Prevotella bivia*, and *Megasphaera* sp. type 1 were found in more than 100 samples. The charts also do not show the count of samples with zero concentrations but only counts of samples with higher concentrations.

The concentrations of the *Lactobacillus* species detected in this study were in the order, *L. iners > L. crispatus > L. jensenii > L. gasseri > L. acidophilus* (**Figure 2**). Additionally, *F. vaginae* and *G. vaginalis* occurred in similar amounts (~10^5^ copies/µL) and were slightly more than the values obtained for *P. bivia* (10^4^ copies/µL). BVAB-1 was the most predominant of the BVABs, and its relative concentration was 44% and 31% more than BVAB-3 and BVAB-2, respectively. Notably, over 98% of the *Megasphaera* species identified in this study belonged to type 1 with a mean concentration of ~10^6^ copies/µL. Of the Mycoplasmas, *U. urealyticum* was found in higher concentrations than *M. hominis*. Although *M. curtisii* was 39% more abundant, it occurred in lower concentrations in vaginal swabs than *M. mulieris*.

### 3.3 Relative abundance, α- and β-diversities

We further investigated the per-sample species relative abundance and richness as well as inter-sample species diversity using alpha and beta diversities. The relative abundance of the species perfectly mirrored their concentration distribution across the samples (**Figure 3**; **Supplementary Figures S1**–**S22**), with *L. iners, L. jensenii, P. bivia, G. vaginalis, Megasphaera* sp. type 1, and *F. vaginae* being more abundant in many samples than the other species. The relative abundance, shown as a heatmap and a box plot (**Figure 4**), represents the abundance of each species across samples, normalized relative to the total concentration of microbial species within each sample. However, the other less abundant species also presented greater variability in relative abundance across the samples, as shown in the outliers (black stars) and absence of boxes (**Figure 4B**).

**Figure 4:**
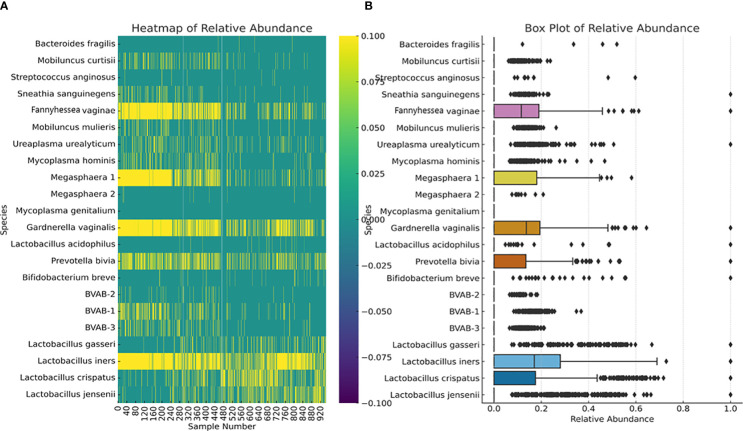
Relative abundance of the 22 species across all the 946 vaginal samples. The heatmap **(A)** highlights the distribution and intensity of species’ relative abundance across all samples, offering a color-coded representation that easily identifies the most prevalent species. The box plot **(B)** provides a statistical summary of each species’ relative abundance, including the median, interquartile range, and any outliers, offering insight into the variability and distribution of abundance for each species. This plot provides insights into the central tendency, spread, and outliers for the relative abundance of each microbial species within the dataset. Median values are represented by the line within each box. Interquartile range (IQR), indicating the middle 50% of the data, is shown by the box itself. Whisker extend to show the range of the data, i.e., 1.5 * IQR from the quartiles. Outliers are points outside the Whisker and are indicated as individual points.

**Figure 5:**
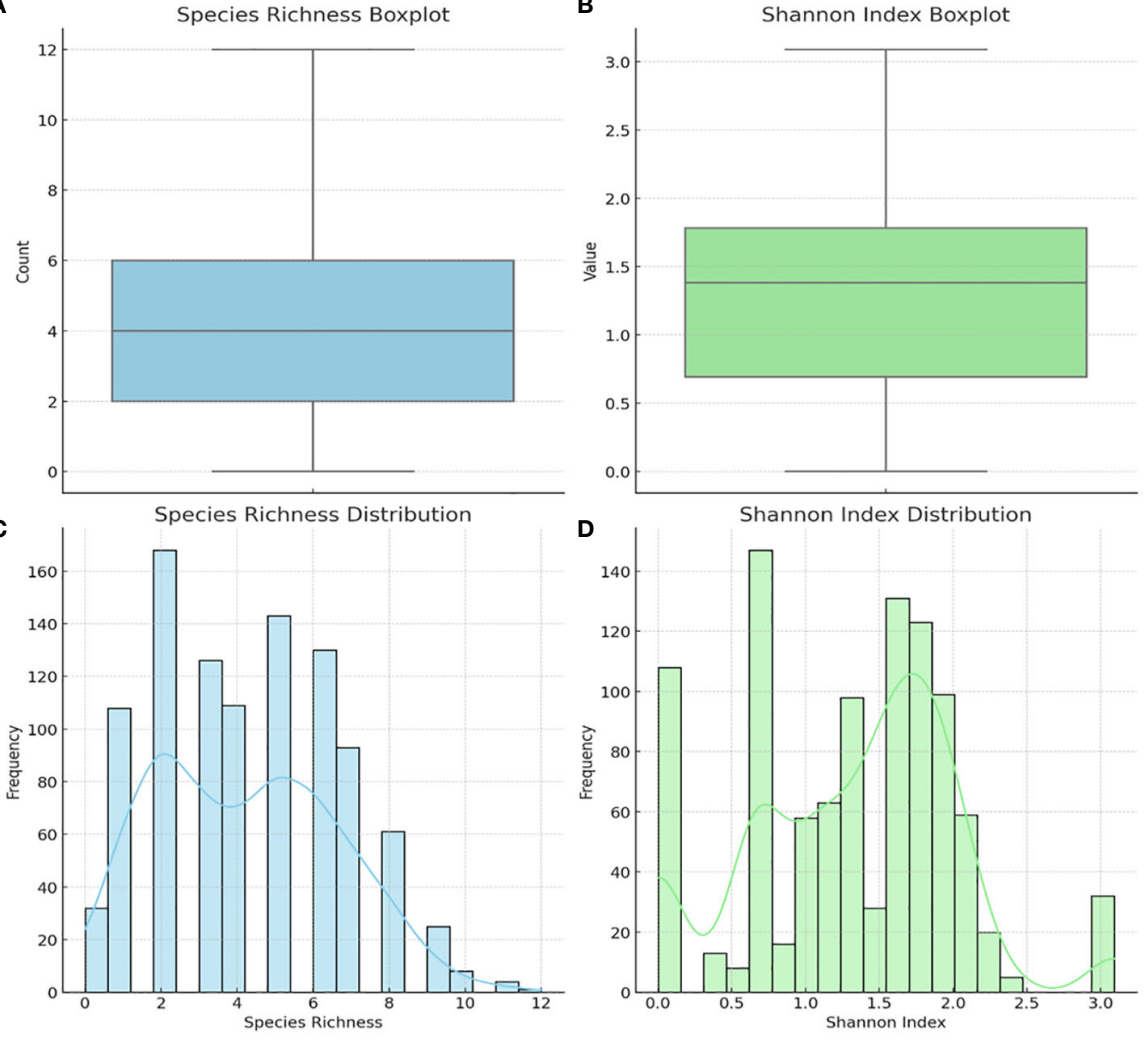
Alpha diversity of the 946 vaginal samples: species richness and Shannon index. Boxplot of Species Richness **(A)**, showing the count of species present across samples. This boxplot provides a clear visualization of the central tendency and variability in species richness. Boxplot of the Shannon Index **(B)**, indicating the diversity value that considers both abundance and evenness of species. Histogram of Species Richness Distribution **(C)**, providing a view of the frequency distribution of species counts across samples. Histogram of Shannon Index **(D)** visualizes the distribution of the Shannon index across samples, reflecting both the abundance and evenness of species. The Shannon index is a more comprehensive measure of diversity, considering not just the presence of species but also their relative abundances.

The alpha diversity of the species within each sample was determined using species richness and Shannon diversity index (**Figure 5**). The species richness shows that 50% of the samples had 2 – 6 species, with 8 – 12 species occurring in 100 – 300 samples (**Figures 5A, C**). The Shannon index, which ranges from 0 (no diversity) to 5 (practically 3.5, i.e., most diverse), showed that 50% of the samples’ diversity index was between 0.7 and 1.8, with 200 – 280 samples having a diversity of > 2.0 (**Figures 5B, D**).

**Figure 6:**
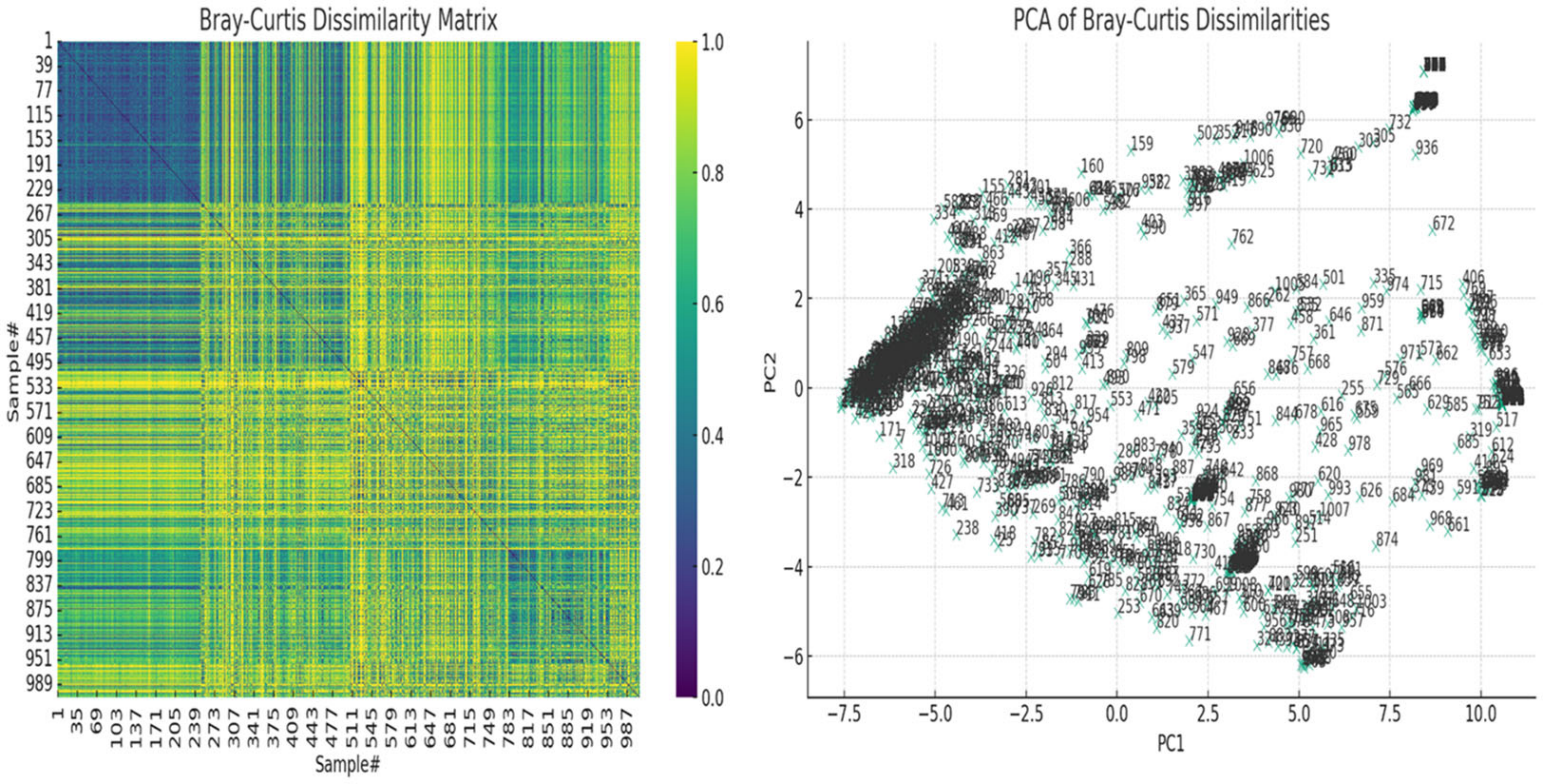
The Bray-Curtis dissimilarity matrix heatmap and associated principal component analysis (PCoA) for beta diversity. On the left, the Bray-Curtis Dissimilarity Matrix heatmap shows the pairwise dissimilarities between the samples. Higher values (closer to yellow, 1.0) indicate greater dissimilarity between samples, while lower values (closer to purple, 0.0) indicate greater similarity. On the right, the PCoA of Bray-Curtis Dissimilarities scatter plot visualizes the samples in a reduced two-dimensional space based on their dissimilarities. Each point represents a sample, and their positions reflect patterns of variation across the samples. Labels on the plot correspond to the sample numbers, helping to identify specific samples within the context of the PCoA. These show how dissimilar the samples were from each other.

Inter-sample (β-) diversity, using the Bray-Curtis dissimilarity index and associated principal component analysis (PCoA), showed that a few of the samples shared strong similarities (**Figure 6**). Samples with close similarity in species diversity are shown as blue (purple) while those with little similarity are shown as yellow. The abundance of yellow in the matrix shows how different most of the samples are from one another (**Figure 6A**). The PCoA chart also reflected this, with a few samples clustering together but most of the samples were spatially separated (**Figure 6B**).

### 3.4 Correlation and co-existence of species

**Figure 7:**
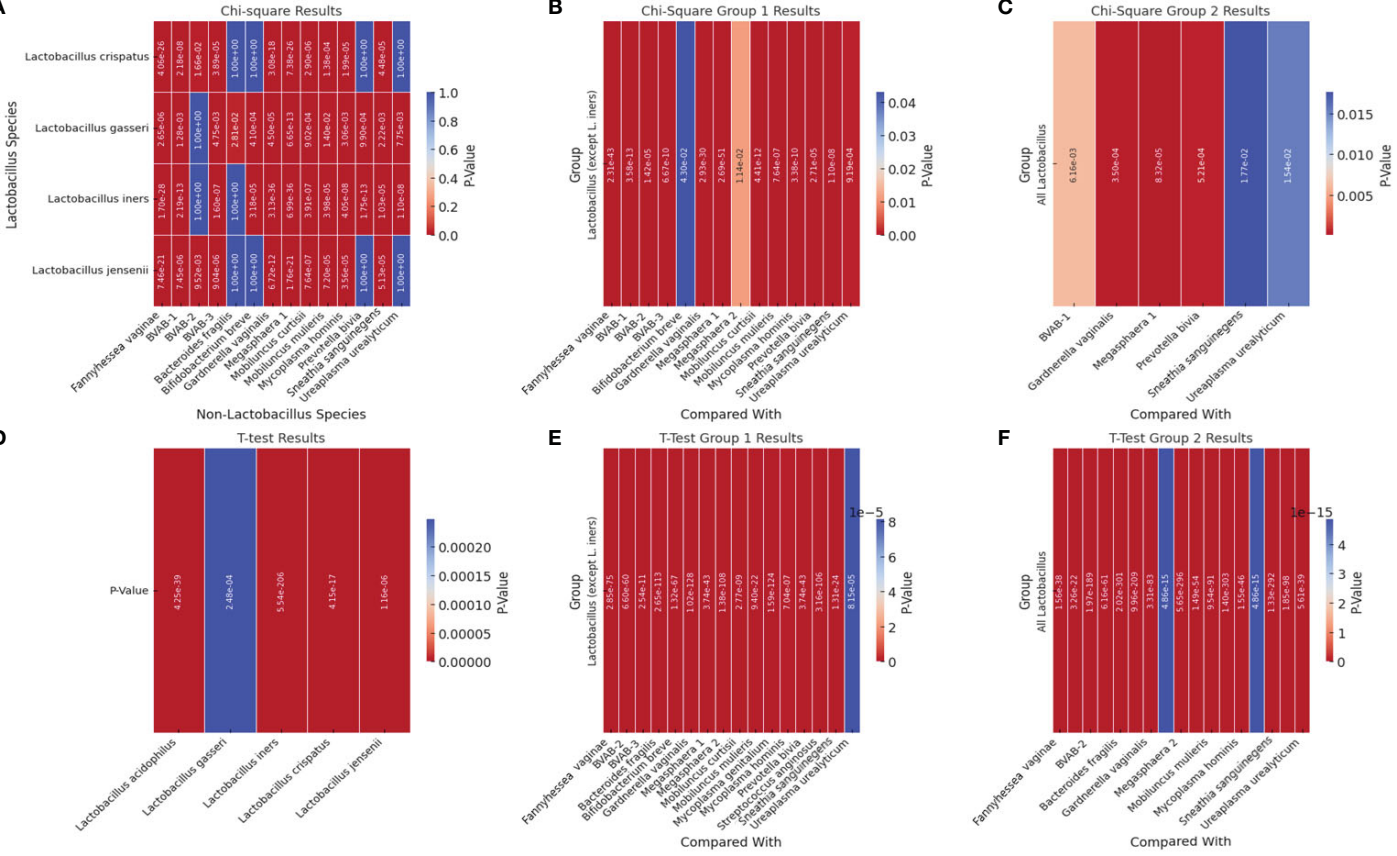
Heatmap of significant p-square values obtained from Chi-square and T-test analysis of pairwise comparisons between *Lactobacillus* and other non-*Lactobacillus* species within the samples. **(A)** visualizes the significant Chi-square p-values between *Lactobacillus* species and non-*Lactobacillus* species. **(B)** (Group 1) visualizes the significant Chi-square p-values of all *Lactobacillus* species (except *L. iners*) against each non-*Lactobacillus* species. **(C)** visualizes the significant Chi-square p-values from grouped comparisons of all *Lactobacillus* species against each non-*Lactobacillus* species. **(D)** shows the significant T-test p-values of all *Lactobacillus* species against all the other non-*Lactobacillus* species grouped together (T-test could not get individual species comparisons because of the structure of the data). **(E)** visualizes the significant T-test p-values from T-Test Group 1, which involves grouped comparisons of all *Lactobacillus* species (except *L. iners*) against each non-*Lactobacillus* species. **(F)** visualizes the significant T-test p-values from the “T-Test Group 2” sheet, which involves grouped comparisons of all *Lactobacillus* species against each non-*Lactobacillus* species. Rows represent different *Lactobacillus* species. Columns represent different non-*Lactobacillus* species. The color intensity indicates the level of significance, with cooler colors (towards blue) indicating lower p-values (higher significance) and warmer colors (towards red) indicating higher p-values (lower significance). Cells filled with a p-value of 1.0 represent non-significant results that were not included in the original significant results table and are shown for completeness.

We performed correlation, Chi-square and T-test analyses of the data to determine the significance of the species-species co-existence in the samples (**Supplementary Table S2**). A heat map of the significant results was generated for easy visualization of the data (**Figure 7**; **Supplementary Table S2**). Unlike the the Chi-square test (**Figures 7A**–**C**), the T-test provided a significant association between the non-*Lactobacillus* species (BVAB-1 was significantly affected by the presence/absence of *L. iners* only but not by the other *Lactobacillus* species)–**Figure 7D** and the presence/absence of the *Lactobacillus* species (without *L. iners*–**Figure 7E** or as a group–**Figure 7F**). In the absence of *L. iners*, the *Lactobacillus* species were not significantly associated with *B. fragilis* (which was only significantly associated with *L. gasseri*) but rather, with *Megasphaera* sp. type 2 (**Figure 7B**). Hence, the Chi-square test provided a more stringent cut-off than the T-test: individually, the *Lactobacillus* species were significantly associated with 14 species (**Figure 7C**).

Therefore, the presence of BVAB-1 and *B. fragilis* were independent of *L. iners.* Furthermore, *L. crispatus* and *L. jensenii* were significantly associated with the presence/absence of the same non-*Lactobacillus* species except *B. fragilis, B. breve, P. bivia*, and *U. urealyticum* (**Figure 7**). Although *L. acidophilus* was included in the Chi-square pairwise association test, it did not yield any significant results with any of the species (**Supplementary Table S2**).

Using Pearson’s correlation coefficient, a clear pattern was observed regarding the co-existence of the 21 species within the vaginal microbiota: all the *Lactobacillus species*, except *L. iners*, inversely correlated with most of the other non-*Lactobacillus* species while species such as BVAB (1–3), *Megasphaera* sp. type 1*, P. bivia, G. vaginalis, F. vaginae, M. hominis, M. mulieris, M. curtisii*, and *S. sanguinegens* were positively correlated with each other. Notably, some species had very little or no inverse correlation with the *Lactobacillus* sp. (except *L. iners*): *S. anginosus, B. breve, U. urealyticum, M. hominis, Megasphaera* sp. type 2, and *B. fragilis. S. anginosus, B. breve*, and *B. fragilis* were the only non-*Lactobacillus* species with an inverse correlation with *L. iners;* the other species had a positive correlation with *L. iners.* The BVAB species did not correlate with each other as BVAB -2 was less correlated with both BVAB-1 and BVAB-3; these latter two species, however, had a strong positive correlation (**Supplementary Figure S23**).

### 3.5 Relative abundance- and species-based diagnostic criteria

All the *Lactobacillus* sp. were bundled together as a marker of a normal vaginal microbiome (Group 1). Owing to the absence of a Nugent score or Amsel data for the samples, we used a species-based criteria to select species that are not found in normal vaginal microbiota: BVAB-1, -2, -3, *B. fragilis*, and *S. anginosus.* These five species were grouped into a species marker (Group 2) to identify BV-positive samples (**Table 1**). Owing to the strong co-existence association between *G. vaginalis, P. bivia, Megasphaera* sp. type 1, and *A. vaginalis* (**Supplementary Figures S10**, **S23**), they were tied together to serve as a marker (Group 3) to fine-tune our criteria in distinguishing between transitional BV, BV-positive, and BV-negative samples. Finally, the remaining eight species were also grouped into a marker (Group 4) to further distinguish between BV-positive, BV-negative, and transitional BV (**Table 1**).

The relative abundance distribution of the species within each of the four biomarker groups above was then used to select cut-off ranges for each group, incorporating a relative abundance-based criteria into the species-based criteria above. This resulted in a four-marker criterion, called the *MDL-BV index*, for diagnosing BV. We trained and tested this two-tier diagnostic *MDL-BV index* on our samples using machine-learning codes (Decision Trees and Random Forests) in Python, adjusting the ranges of each (species group) biomarker’s relative abundance until an optimal range per (species group) biomarker was found. The results of the *MDL-BV index’s* classifications were manually verified to ensure its veracity.

Observations made during the training and testing process made us include the following four instructions into the *MDL-BV index* code to enable categorization of all types of vaginal samples into their respective BV states: 1. When a sample falls within both BV-negative and transitional BV, assign it to transitional BV; 2. When a sample falls within both BV-positive and transitional BV, assign it to BV-positive; 3. When at least three of a sample’s four groups/biomarkers’ relative abundances fall within a single BV state and only one falls in another BV state, override the single disagreement, and assign or classify the sample to the BV state with which the three relative abundances agree; 4. When biomarkers 2 and 4 have a relative abundance of zero, then a relative abundance of 0 – 30% for Group 1 and 70 – 100% for Group 3 is BV-positive; a relative abundance of 30 – 50% (Group 1) is transitional BV and 60 – 100% (Group 1) is BV negative; 5. When every group is zero and only group 4 is 100%, define it as BV positive; 6. When all groups are zero, filter out that sample as there are no results to report (**Table 1**).

The final *MDL-BV index* was then tested on the data used in this study and 490 samples were classified as BV positive, 335 samples were classified as BV negative, and 151 samples were classified as Transitional BV. A manual verification of these classifications found them to be accurate, based on the MDL-BV index ranges. The relative abundance of each of the four biomarkers/groups and the final BV status classification based on these relative abundances, produced by the Python code, is shown in **Supplementary Table S3** and summarized in **Table 1**.

### 3.6 Demographics affect BV

**Figure 8:**
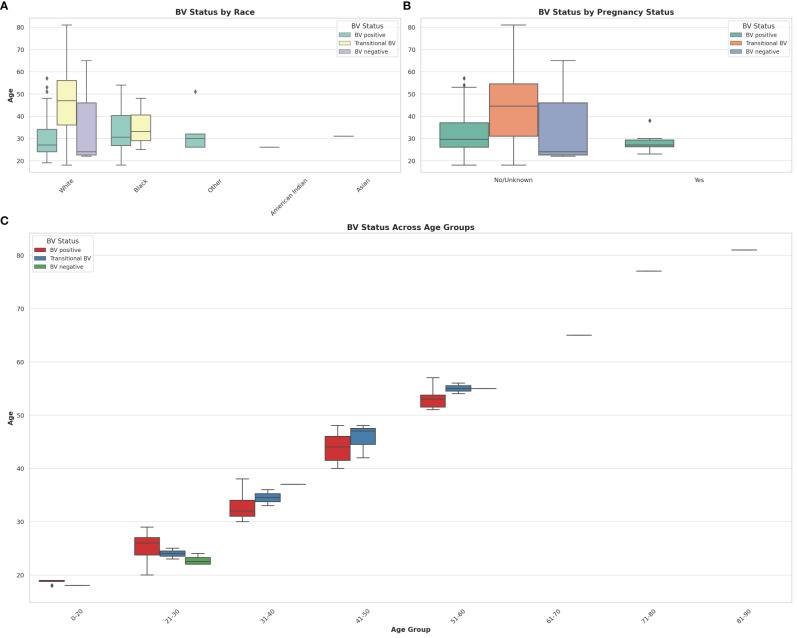
Box and Whisker plots showing the distribution of BV-positive, Transitional BV, and BV-negative samples across different races, ages, and pregnancy status. **(A)** shows the distribution of BV status across different races. **(B)** illustrates the distribution of BV status among pregnant and non-pregnant women. **(C)** visualizes the distribution of BV status across different age groups. The horizontal axes show the ages for the different classifications and distributions. The box colors are not the same for all the three plots and their respective keys in the upper right or left corners designate the correct interpretation of each color. The Whisker show the upper and lower quartiles while the boxes show the 25^th^ and 75^th^ percentile range (50% of the population) while the stars show outliers. Chi-square and Kruskal-Wallis tests respectively showed that there were no significant effect/association between race, pregnancy status, and age groups with BV.

The effect of age, pregnancy status, and race on BV status was analyzed using a correlation heatmap (**Supplementary Figure S24**) and Box and Whisker distribution plots (**Figure 8**). Notably, almost all BV-negative cases were found within the White population. Transitional BV was found only among White and Black people, with BV-positive cases being widely distributed among the Black population aged 28 – 40; most White women who had BV were aged between 25 and 35. The age groups of the BV-positive and transitional BV populations were almost the same among Black people but very different among White people. The ‘Other” race (including Latinos/Hispanics) also had substantial BV, with their ages between a tight window of 28 to 32 years. Although the number of Asian and American Indian samples was relatively few, they were all BV-positive and fell within the median age range.

It is notable that most women who were pregnant were also BV-positive, with no transitional and BV-negative status being found among them. Further, the age differences between BV-positive pregnant women (24 – 30 years) and non-pregnant women (28 – 38 years) were wide. The Box plot in 8C shows a similar occurrence of BV among women aged 21 – 60, with very little incidence being found among women aged above 60 and below 20. Notably, transitional BV cases were more common among women aged between 41 and 50 than among the other age groups. BV-negative cases were mostly found among women aged between 21 – 30 years.

Chi-square tests indicated that there was no statistically significant association between race and BV status (*P* = 0.217), and between pregnancy status and BV status (*P* = 0.527) at significance levels (*P* < 0.05). The Kruskal-Wallis test, which was used to assess whether there are statistically significant differences in age distributions between the BV positive and BV negative groups, found no statistically significant differences in the age distributions between the BV positive and BV negative groups (*P* = 0.820) at significance levels (*P* < 0.05). Therefore, age by itself, may not be a distinguishing factor between these two groups within the dataset analyzed.

### 3.7 Demographics affect variations in relative abundance

#### 3.7.1 Variance of Lactobacillus shows lower abundance in BV-positive samples and higher abundance in BV-negative samples.

**Figure 9:**
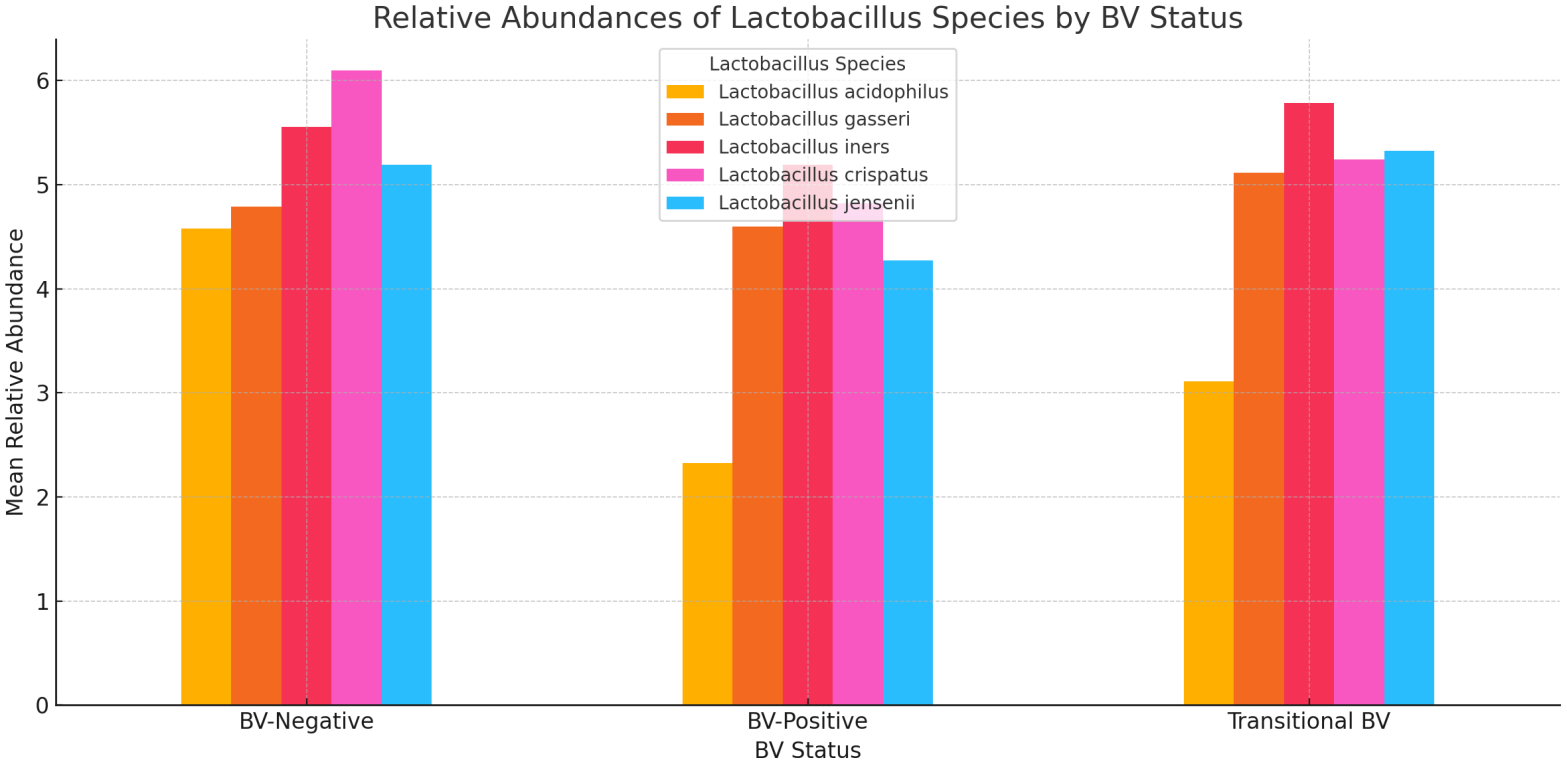
A bar chart displaying the relative abundances of five *Lactobacillus* species across three BV-Negative, BV-Positive, and Transitional BV statuses. Overall, Lactobacillus species tend to have higher mean abundances in BV-Negative samples, indicating their potential protective role against BV.

The relative abundance of *Lactobacillus* sp. showed that while *L. acidophilus, L. jensenii*, and *L. crispatus* had low mean abundance in BV-positive and transitional BV samples, and high mean abundance in BV-negative samples, *L. iners* and *L. gasseri* did not exhibit such phenomena. The mean abundance of *L. gasseri* was similar across all three BV states (marginal variance). *L. iners* was more prevalent in transitional BV samples and less abundant in both BV-negative and BV-positive samples; its mean relative abundance in both BV-positive and –negative species was similar. The greatest variance was observed in *L. acidophilus*’ relative abundance levels between BV-positive and BV-negative samples (**Figure 9**).

#### 3.7.2 Variance of Lactobacillus and vaginal anaerobes show differences in pregnancy

**Figure 10:**
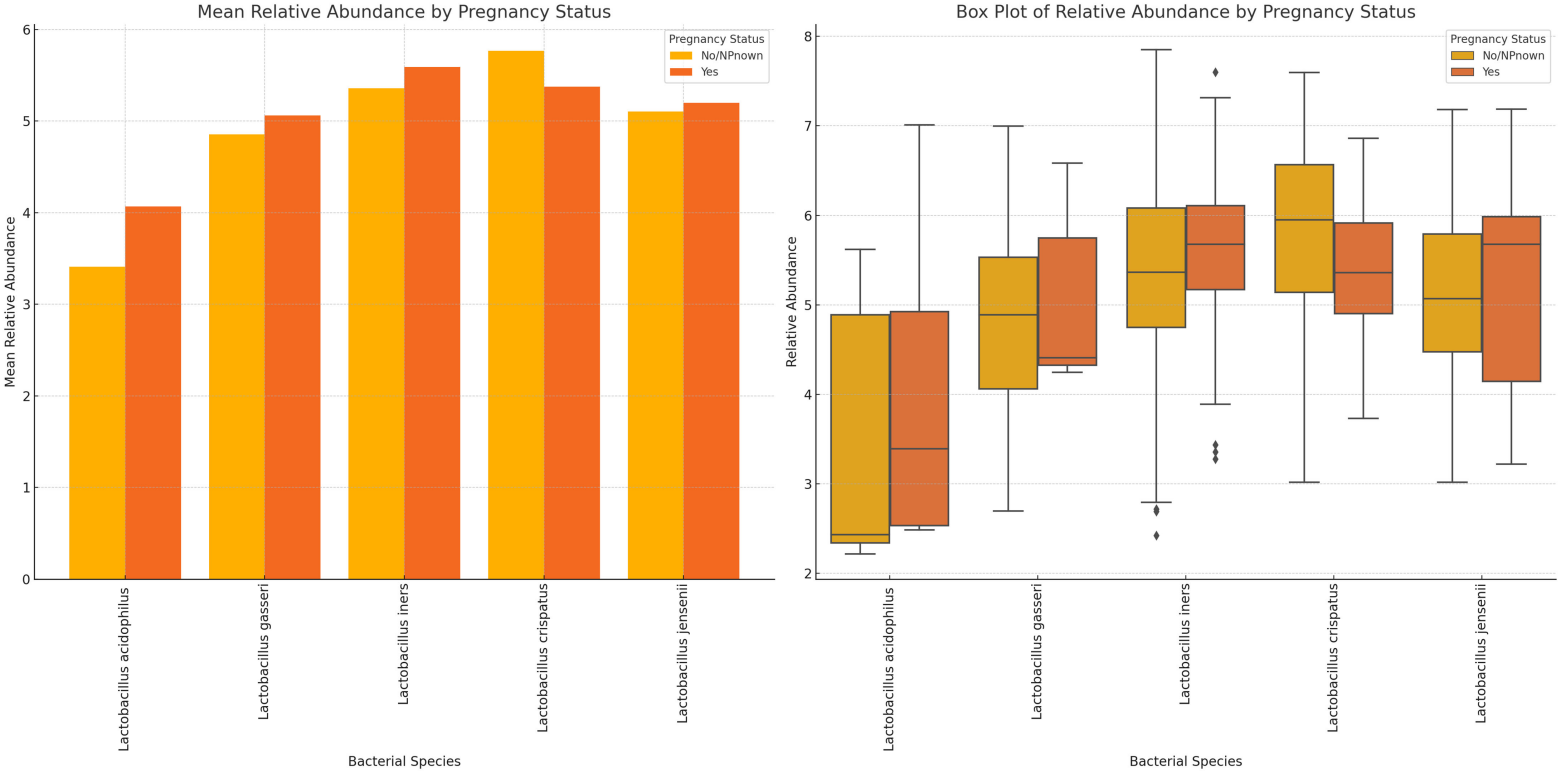
Plots displaying the relative abundances of five *Lactobacillus* species in vaginal samples collected from pregnant and non-pregnant women. *L. crispatus* was the highest in non-pregnant women while *L. iners* was highest in pregnant women. In all, the variance between these two cohorts were not so wide. *L. acidophilus* had a wider spread/distribution of relative abundances across the samples for the two cohorts.

The mean abundance of *L. acidophilus, L. gasseri*, and *L. iners* were slightly higher in pregnant women than in non-pregnant women. *L. crispatus* and *L. iners* had the highest relative abundance in non-pregnant and pregnant women, respectively. While *L. jensenii’s* mean relative abundance in both pregnant and non-pregnant women was similar, *L. crispatus* stood out as the only *Lactobacillus* with a slightly higher relative abundance in non-pregnant women than in pregnant women. Notably, the variance between the mean relative abundance of *L. acidophilus* in pregnant and non-pregnant women was markedly wider than the other species (**Figure 10**; **Supplementary Figure S25**). Except for *L. crispatus*, the mean relative abundance of the *Lactobacillus* sp. was marginally higher in non-pregnant women than in pregnant women (**Supplementary Figure S25**).

**Figure 11:**
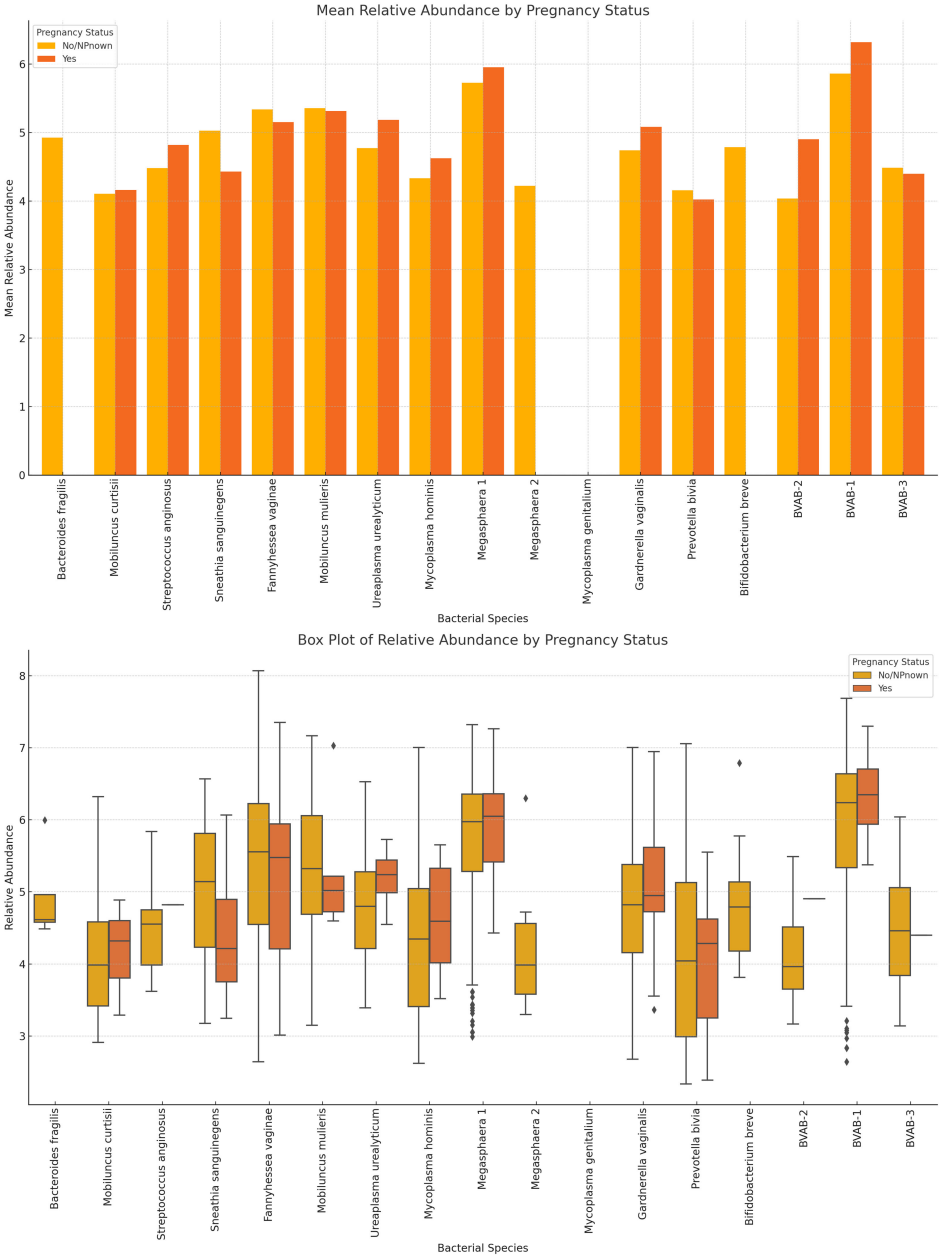
The mean and absolute relative abundances of various non-*Lactobacillus* bacterial species across pregnancy statuses (Pregnant and Not Pregnant). Species such as *B*. *fragilis, Megasphaera* sp. type 2, and *B*. *breve* were only present in non-pregnant women while the *S. sanguinegens* variance between pregnant and non-pregnant women was higher (in non-pregnant women). BVAB-3, *P. bivia, F. vaginae*, and *M. mulieris* had slightly higher mean abundances in non-pregnant women than in pregnant women. The rest had higher or slightly higher mean abundances in pregnant women than in non-pregnant women.

The variance in mean relative abundance of the facultative and obligate anaerobes in pregnant and non-pregnant women differed per species (**Figure 11**; **Supplementary Figure S26**). Instructively, *B. fragilis*, *Megasphaera* sp. type 2, and *B. breve* were only present in non-pregnant women and absent in pregnant women. BVAB-3, *P. bivia, M. curtisii, F. vaginae and M. mulieris* had similar levels in both cohorts, with a slight decrease in four out of the five species for pregnant women; only *M. curtisii* was slightly higher in pregnant women. The differential abundance of BVAB-1, BVAB-2, *G. vaginalis, Megasphaera* sp. type 1, *S. anginosus*, and *U. urealyticum* was higher in pregnant women. *S. sanguinegens* alone was higher in non-pregnant women (**Figure 11**; **Supplementary Figure S26**). Non-pregnant women had higher diversity of anaerobes (n = 16 species) and generally lower mean relative abundance than pregnant women (n = 13 species) (**Supplementary Figure S27**).

#### 3.7.3 Variance of Lactobacillus and vaginal anaerobes show differences by age group

**Figure 12:**
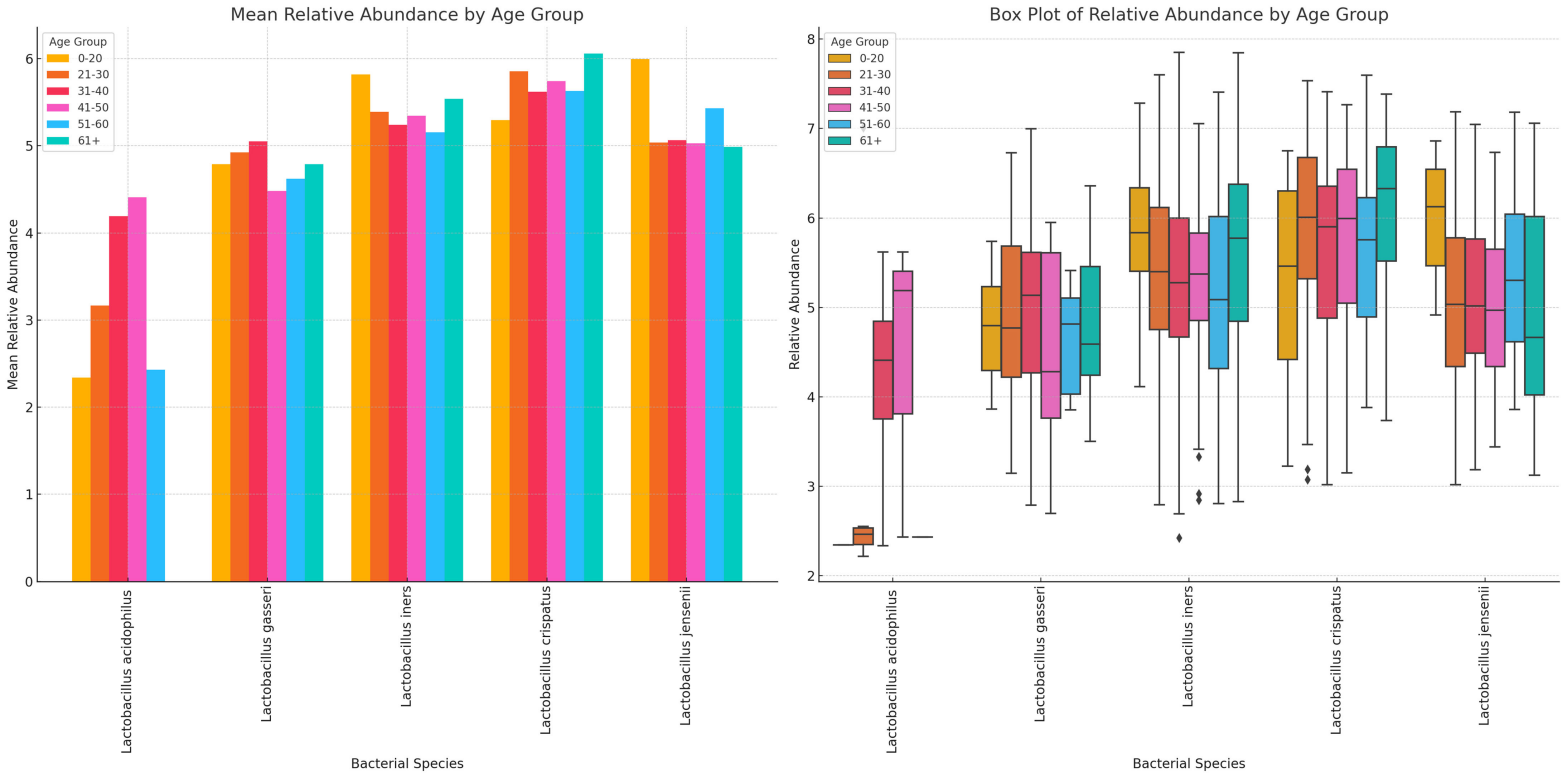
Charts displaying the mean and absolute relative abundances of five *Lactobacillus* species across different age groups (0–20, 21–30, 31–40, 41–50, 51–60, 61+). *L. acidophilus* was more abundant in age 41–50 group, followed by age 31–40 and 21–30 groups. *L. crispatus* was higher than the other species in all age groups except 0–20 group, where *L. iners* and *L. jensenii* were higher. Except. *L. acidophilus*, the other species had a wider distribution of relative abundances for the different age groups.

*L. acidophilus* was absent in females aged 61+ years and highest in those aged 41–50 years while *L. crispatus* was highest in those aged 61+ years. *L. jensenii* was most dominant in the 0–20 years’ cohort while *L. gaserri* had similar abundance levels across all age groups, except the 51–60 age group where it was slightly lower. *L. iners* was also most abundant in the 0–20 years group (**Figure 12**; **Supplementary Figure S28**).

**Figure 13:**
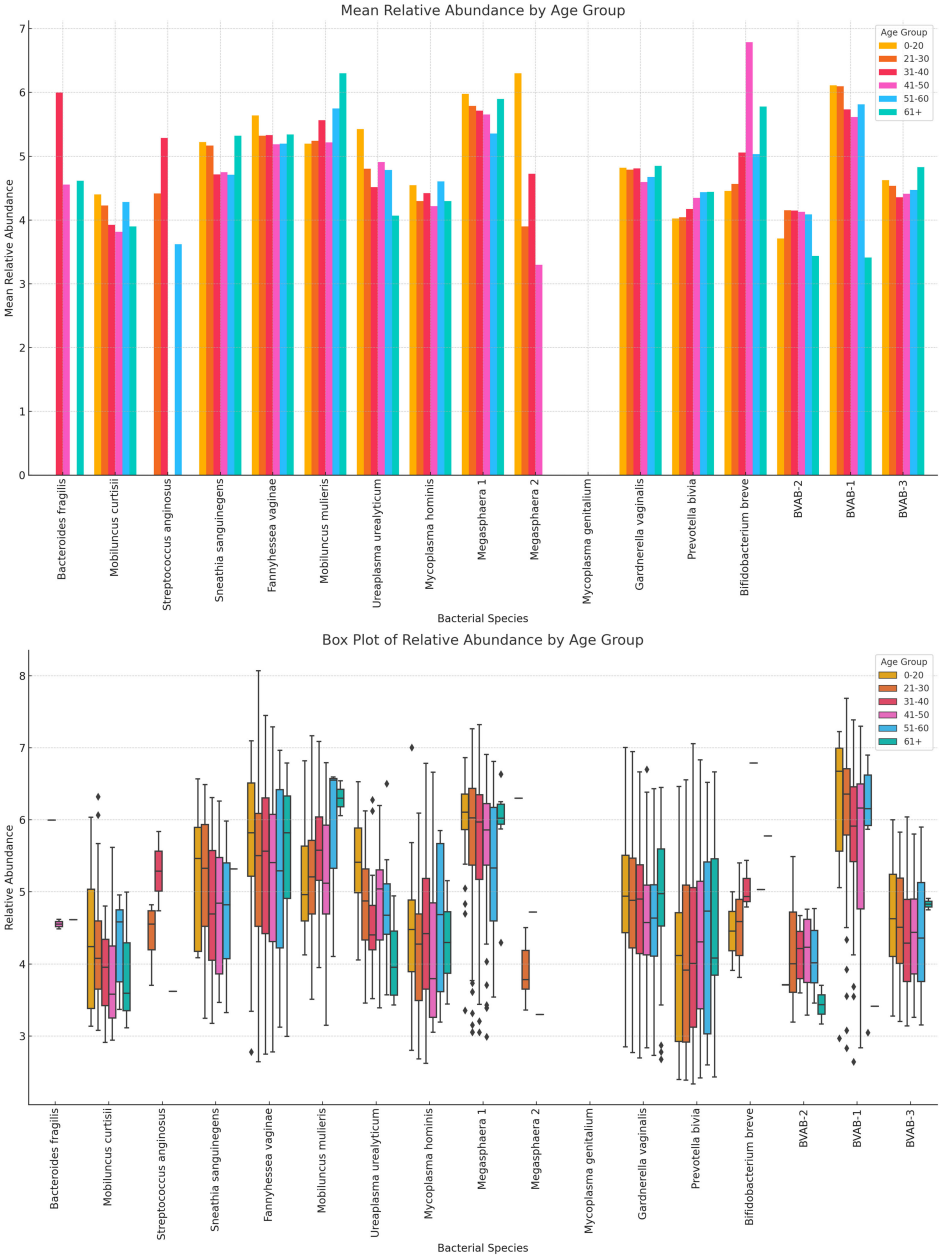
The bar chart displays the mean and absolute relative abundances of various non-*Lactobacillus* bacterial species across different age groups. Fourteen species were present in age 0–20 group, 15 were found in age 21–30, 41–50, and 51–60 groups, 16 was found in age 31–40 group, and 13 were found in 61+ age group. Species such as *P. bivia, F. vaginae* and *S. sanguinegens* had broader spread of relative abundances across the age groups. Overall, non-*Lactobacillus* bacterial species are primarily observed in the 21–30 age group, with little to no data available for other age groups. This indicates a higher microbial diversity in this age group compared to others.

Likewise, the anaerobic bacterial species differed between age groups, with *S. sanguinegens, F. vaginae, M. mulieris, Megasphaera* sp. type 1, *G. vaginalis, B. breve*, and BVAB-3 being very dominant in females aged 61+ years. *B. breve* was very abundant in the 41–50 years group while *Megasphaera* sp. type 2 was also very prevalent in the 0–20-year group. *B. fragilis, S. anginosus, M. mulieris*, and *Megasphaera* sp. type 2 were also highly prevalent within the 31–40-year cohort. Notably, within the 21–30, 31–40, and 41–50 age groups, most of the species were mostly of similar or slightly higher relative abundance; the outliers are described above (**Figure 13**; **Supplementary Figure S29**).

There were 13 species in the 0–20 group, 15 in the 21–30, 41–50, and 51–60 groups, and 13 in the 61+ group, showing that the diversity increases after age 21, plateaus until age 60 and decreases after age 61. The highest relative abundances were mainly within the age 21–50 bracket for majority of the species (**Figure 13**; **Supplementary Figure S29**).

#### 3.7.4 Variance of Lactobacillus and vaginal anaerobes show differences by age group

**Figure 14:**
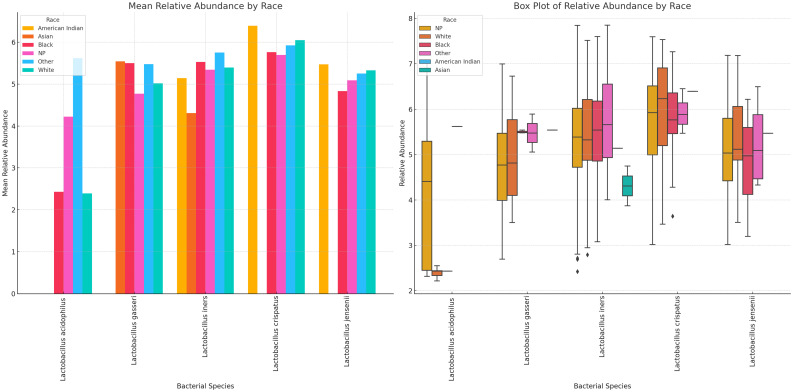
Mean and absolute relative abundance distribution of *Lactobacillus* species in different racial/ethnic groups. White, Black, and other races had more diversity of *Lactobacillus* sp. while Asian people had very little diversity. *L. acidophilus* was higher in other (Hispanics and Pacific Islanders) races than White and Black people. *L. crispatus* was higher in American Indians and White people while *L. gasseri* and *L. iners* were higher in Black and Other races. *L. gasseri* was also higher in Asian people. The relative abundance spread of the species differed per race. NP means not provided. The Whisker show the upper and lower quartiles while the boxes show the 25^th^ and 75^th^ percentile range (50% of the population) while the stars show outliers.

Variance of Lactobacillus and vaginal anaerobes show diversity and abundance vary by race *Lactobacillus* sp. and non-*Lactobacillus* sp. across the different racial groups. Specifically, *L. iners, L. crispatus*, and *L. jensenii* were present in most of the races, albeit *L. crispatus* was higher in most races and *L. acidophilus* was consistently lower than the other *Lactobacilli. L. iners* was very common in Asian people compared with the other *Lactobacilli; L. acidophilus* was higher in Others (Hispanics and Pacific Islanders) races and lower in the remaining races. *L. gasseri* was high in Asian, Black, and Others (Hispanics and Pacific Islanders) races across all races. Notably, *L. crispatus* was the highest *Lactobacilli* in White people (**Figure 14**; **Supplementary Figures S30**, **S31**).

**Figure 15:**
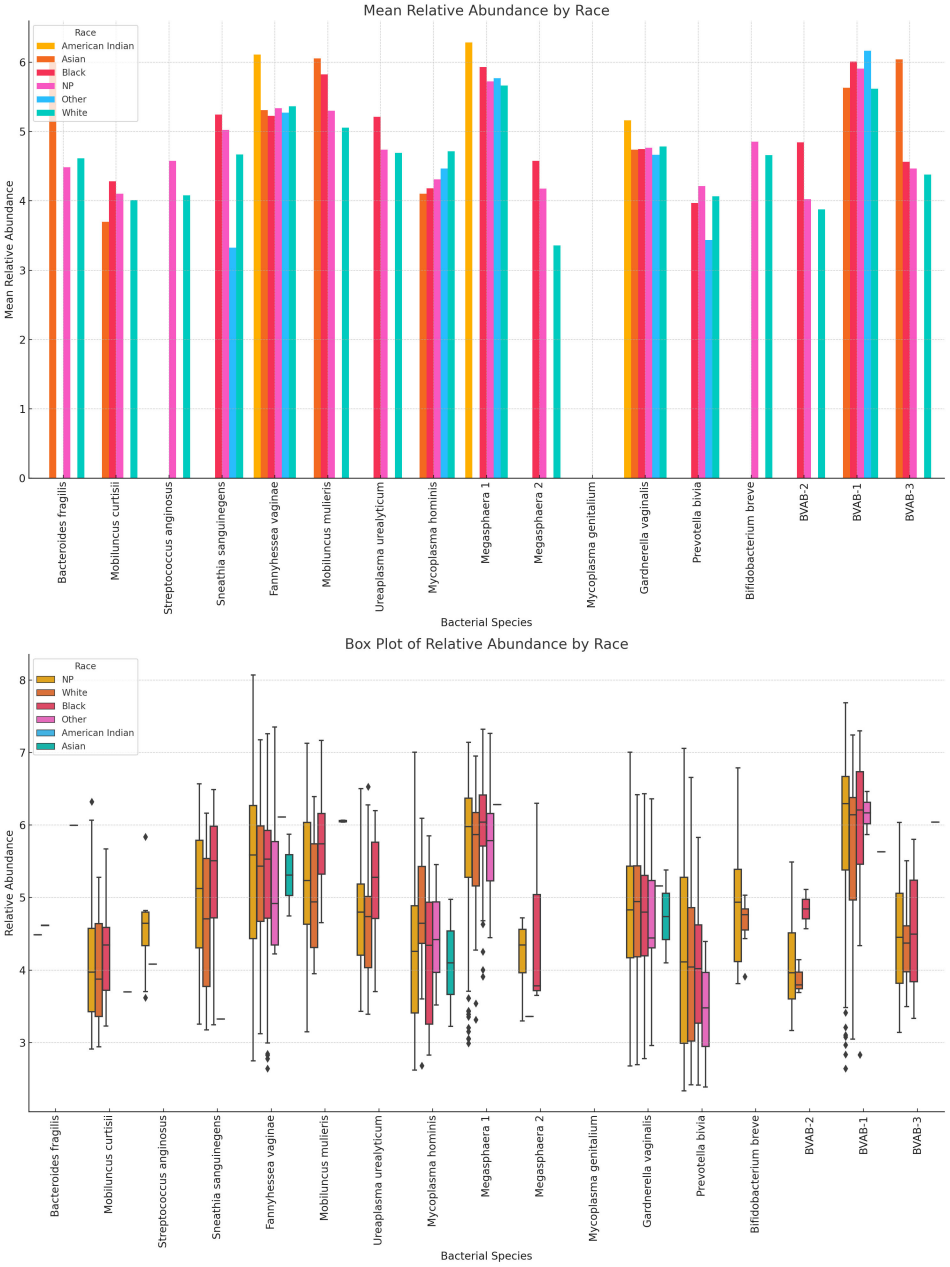
Relative abundance distribution of non-*Lactobacillus* species in different racial/ethnic groups. For many of the species, there were a higher relative abundance among Black people than White people, explaining the higher prevalence of BV in Black people than White people. *F. vaginae, Megasphaera* sp. type 1, and *G. vaginalis* were highest in American Indians. Relative abundance distribution of the species differed per species. The Whisker show the upper and lower quartiles while the boxes show the 25^th^ and 75^th^ percentile range (50% of the population) while the stars show outliers. NP means not provided.

Racial variations in relative abundance were most obvious in *B. fragilis, S. anginosus, M. hominis, Megasphaera* sp. type 2*, P. bivia, B. breve*, and BVAB-2. For many of the species, there were a higher relative abundance among Black people than White people: *Mobilincus* sp.*, S. sanguinegens, U. urealyticum, G. vaginalis, Megasphaera* sp., and BVAB-2. *B. fragilis M. mulieris, G. vaginalis*, BVAB-1 *and* BVAB-3 were highly abundant in Asian people. Among White people, *F. vaginalis, M. hominis, S. sanguinegens, Megasphaera* sp. type 1, *G. vaginalis, P. bivia, B. breve*, BVAB-2 and BVAB- 3 were dominant (**Figure 15**; **Supplementary Figures S32**, **S33**).

## 4 Discussion

Unlike other infections with single etiological agents, BV is a polymicrobial infection with no clear consensus on the specific bacterial species responsible for the altered vaginal microbiota state (Mondal et al., 2023; Sobel and Vempati, 2024). The exact contributions of BV-associated microbes to the pathogenesis of BV, which may be relevant for accurate diagnosis and therapeutics, remain unresolved. Nevertheless, the occurrence of normal, transitional, and abnormal vaginal microbiota states is dependent on the composition and interactions of the different *Lactobacillus* and anaerobic species present (Onderdonk et al., 2016; Lynch et al., 2019; Chee et al., 2020; Drew et al., 2020). To address this gap between the vaginal microbiome dynamics and the current BV diagnostic tests’ limitations, a new qRT-PCR test that detects and measures the gDNA concentrations of 22 species found in all the various conditions of the vaginal microbiome was designed. The relatively shorter turnaround time of this test and the ease of adoption in a routine diagnostic laboratory makes it possibly more valuable than the current classical tests (Muzny et al., 2023; Swidsinski et al., 2023).

With such a broad spectrum of bacterial species, the resolution of the test is enhanced to efficiently distinguish between normal, abnormal, and transitional microbiota. The test has been validated with 95 – 100% sensitivity and specificity with a short turnaround time of 8 hours (from sample reception). In studies where results from PCR or qPCR studies targeting 2 – 13 species have been compared with Amsel’s criteria or Nugent’s score, the results have been highly sensitive and specific (Menard et al., 2008; Malaguti et al., 2015; Lynch et al., 2019; Drew et al., 2020; Vodstrcil et al., 2021). We are therefore confident that subsequent studies with our test, using Nugent score or Amsel’s criteria-diagnosed samples, may equally yield similar or better results with its larger bacteria target spectrum. Although this study is limited by the inability to compare the current results with clinical presentation data, its sensitivity and specificity in detecting the controls used confirm its efficiency.

In fact, the test’s diagnostic capability is further appreciated when the make-up and sample size of our vaginal specimens are considered: 946 vaginal samples from a wide spectrum of ages (18 – 83), races (White, Black, Asian, American Indian, and “Other (Hispanics and Pacific Islanders)”, not to mention the races of most women who failed to state their race), and pregnancy status. The Bray-Curtis dissimilarity matrix and the principal component analysis (PCoA) showed how dissimilar the samples were as few of the samples clustered together (**Figure 6**). The sample differences were further highlighted by the species richness (α-diversity) of the samples in which the number of species per sample ranged from 2 – 12, with 50% of the samples having 2 – 6 species per sample. Instructively, the Shannon index further clarified this observation by showing that the species diversity per sample was low, with most samples having an index between 0.8 and 1.8 and the most diverse sample having an index of 3.2 (out of 3.5).

Thus, although the samples were dissimilar in terms of composition, their diversity was relatively low, which could be characteristic of a healthy vaginal microbiome if the relative abundance of *Lactobacillus* sp. is high (Balashov et al., 2014). However, it is worth noting that the Shannon diversity is affected by the number of species sampled and the vaginal microbiome is naturally not as diverse as the gut microbiome (Javed et al., 2019; Muzny et al., 2020; Abou Chacra and Fenollar, 2021). Hence, although the 22 species used are more than any PCR test, it may not represent all the species in the vaginal microbiota. For instance, the alpha diversity of the samples was calculated through a direct count of the number of species observed in each sample without using any estimators or indices. Hence, it does not account for undetected species or attempt to estimate the total species richness in the samples, which methods like the Chao1 index aim to do. Notwithstanding, the representative nature of the samples used in this study is self-evident.

To our knowledge, no qPCR assay has the same broad-spectrum species target, making this assay an important innovation and the first to do so. Whereas other assays have used only two or up to 13 species (Malaguti et al., 2015), not all of the species were quantitatively detected as was done in this assay (Brotman and Ravel, 2008; Menard et al., 2008; Malaguti et al., 2015; Drew et al., 2020). This is despite the calls for increasing the bacterial spectrum for molecular BV diagnosis, focusing on *Megasphaera, G. vaginalis, F. vaginae*, and other anaerobes (Brotman and Ravel, 2008; Menard et al., 2008; Lynch et al., 2019). Moreover, owing to the biofilm-forming nature of these BV-associated species, which leads to immune evasion, high treatment failure rates and recurrence, a shorter-turnaround diagnostic tool is merited (Cangui-Panchi et al., 2023; Muzny et al., 2023). This study answers that call.

By both detecting and quantifying the 22 species in vaginal samples, we were able to obtain a clearer picture of the vaginal microbiome’s composition and make a better diagnosis of its condition; a feat only achievable with whole-genome metagenomics (Asante and Osei Sekyere, 2019; Osei Sekyere et al., 2020; Osei Sekyere et al., 2023). For instance, although BVAB-1, -2, and -3 were detected together in the same study (Fredricks et al., 2005), BVAB-2 has been commonly tested as a marker of BV positivity without BVAB-1 and -3. Yet, this study showed that BVAB-2 is the least prevalent/abundant among these three species, with BVAB-1, followed by BVAB-3, being the most common. A Brazilian study using 223 BV-positive samples also reported the same findings regarding the relative abundance of BVAB-1, BVAB-2, and BVAB-3, affirming our observations (Malaguti et al., 2015). A positive association between BV and high-risk Human Papilloma Virus (HPV) genotypes has been reported, owing to the occurrence of BVAB 1 and 3, and other BV-associated- bacteria in women co-infected with HIV and HPV (Naidoo et al., 2022). The same study also showed that the presence of BVAB 1 and 3 had an elevated likelihood of increasing the severity of cervical neoplasia in this population (Naidoo et al., 2022). Nevertheless, these species are not as closely related as initially thought as a recent phylogenetic analysis identified BVAB 1, 2, and 3 to be “*Clostridiales genomosp*.”, *Oscillospiraceae bacterium* strain CHIC02, and *Mageeibacillus indolicus*, respectively (Osei Sekyere et al., 2023).

Additionally, the relative abundance of the various species identified by this test mirrors what has been reported (Malaguti et al., 2015; Bayigga et al., 2019; Abou Chacra et al., 2021; Zhang et al., 2022; Powell et al., 2023), with *Lactobacillus* sp. (except *L. acidophilus*), *G. vaginalis, A. (F.) vaginae, P. bivia*, and *Megasphaera* sp. type 1 being the most dominant and widely distributed (**Figures 2**, **4**; **Supplementary Figures S1**–**S22**). The absence of *M. genitalium* [which was found in low abundance in other studies using BV-positive samples (Malaguti et al., 2015)], and lower abundance and prevalence of *L. acidophilus, M. hominis, U. urealyticum, Megasphaera* sp. type 2*, Mobiluncus* sp.*, S. sanguinegens, B. breve*, and *S. anginosus* are also consistent with other findings (Atassi et al., 2019; Chee et al., 2020; Argentini et al., 2022; Wu et al., 2022), further crediting the diagnostic efficiency of this test.

Overall, the *Lactobacillus* species had higher mean abundances in BV-negative samples, lower abundances in BV-positive samples, and in-between for transitional BV, indicating their potential protective role against BV (**Figure 9**). The higher relative abundance of *L. iners* in pregnant women and transitional BV samples could further suggest that *L. iners* play a crucial role in transitioning the vaginal microbiome from BV-negative to BV-positive. The transitioning effect of *L. iners* has been investigated and reported earlier and these observations further throws more light on the veracity of this conclusion (Zheng et al., 2021), albeit other studies provide conflicting findings and call for additional studies (Carter et al., 2023).

It was observed from the correlation, and co-existence analysis that the five *Lactobacillus* sp. mostly co-existed together while *G. vaginalis, A. (F.) vaginae, P. bivia*, and *Megasphaera* sp. type 1 also co-existed in the same samples (**Figures 4**, **7**; **Supplementary Figures S1**–**S23**). The Chi-square and T-tests also largely agreed with the significant association between the *Lactobacillus* sp. and the non-*Lactobacillus* species, with few exceptions (**Figure 7**). This was used to form two separate groups of species-based biomarkers. Furthermore, the BVABs, *S. anginosus*, and *B. fragilis*, which are known to be mainly associated with BV microbiomes and absent in normal vaginal microbiomes (Weyers et al., 2009; Matu et al., 2010; Malaguti et al., 2015; Schmidt et al., 2015; Barrientos-Durán et al., 2020), were also teased from the remaining non-*Lactobacillus* species and grouped into another biomarker group. The remaining eight species were then also bundled together into another fourth group (**Table 1**). Using the relative abundance distributions of each species (**Figure 4**; **Supplementary Figures S1**–**S22**), the relative abundance range for each of the four species biomarker groups was set to form the *MDL-BV index*, which was then further trained and tested on the data using machine-learning algorithms (Decision Trees and Random Forests) to diagnose BV. A relative abundance-based approach was adopted over a nominal concentration value because concentrations vary from sample to sample, and the swabbing method of sample collection among clinicians is not standardized; hence, samples collected by each swab is not quantitative.

The *MDL-BV index* has a high cut-off range for negative BV status (≥70% *Lactobacillus* sp. relative abundance) to ensure that samples diagnosed as normal were BV-negative in verity. It also errs on the side of caution by classifying samples that meets the criteria of both BV-negative and transitional or transitional and BV-positive, as transitional BV or BV-positive respectively. The training of the model on the data further revealed certain intricacies and nuances in the distribution of the species and their relative abundances, which were used to refine it by including more details and rules. For instance, in situations where only two of the four biomarkers had a relative abundance score, the ratios were adjusted (**Table 1**). This strengthened the diagnostic efficiency of the *MDL-BV index*, resulting in a final classification of the samples as 491 BV-positive, 318 BV-negative, and 137 transitional BV. A manual verification of the results confirmed the veracity of the classifications (**Supplementary Table S3**).

Instead of using just two biomarkers involving all *Lactobacillus* sp. and all non-*Lactobacillus* species (Balashov et al., 2014), this index uses four biomarkers, which further refines the ability of the index to correctly distinguish between transitional BV or BV-positive microbiomes. Evidently, a correct molecular diagnosis of BV is critical to its treatment and monitoring, making this test and index very important in gynecology and obstetrics. The next stage of this research is to train the model on large datasets with Nugent scores/Amsel criteria as a means of further enhancing it and strengthening its proof of concept in BV diagnosis.

The distribution of BV among the different demographic variables viz., age, race, and pregnancy status showed important differences, albeit none of the differences were statistically significant (**Figure 8**). Specifically, although the number of vaginal samples from Black people was lower than that from White people, there were more BV-positive samples from the former than from the latter. While most of the vaginal samples from White people were BV-negative, those from Black people were either BV-positive or transitional. Notably, the samples from the “Other” races (which includes Hispanics/Latinos and Pacific Islanders), Asians, and Native American Indians, although fewer in number, were also BV-positive, with none being negative. Furthermore, the mean age and age range for White people with transitional BV were higher than those of all the other races, including the mean age of White people with abnormal vaginal microbiomes (**Figure 8**). Hence, although these differences were not statistically significant, they concur with other studies that show a higher prevalence of BV among Black and Hispanics than among White people and Asians (Peebles et al., 2019). This agreement between our data and other studies further confirms the accuracy of our *MDL-BV index*.

A further analysis of the racial vaginal microbiome composition showed that Black women had a higher prevalence of many of the anaerobes implicated in the BV pathogenesis, including *G. vaginalis, F. vaginae*, and *Megasphaera* sp., followed by Hispanics and Asian women who also had higher abundance of these BV-associated species than Caucasians (**Figure 15**; **Supplementary Figures S32**, **S33**). These findings are not singular as other studies have also identified the same. Particularly, Borgdoff et al. (2017) found a higher abundance of *L. crispatus* among Caucasians than Black and Asian women and a higher *G. vaginalis* and *L. iners* in Ghanaian and Asian (and Mediterranean) women, respectively (Borgdorff et al., 2017). Contrarily however, Roachford et al. (2022) also found a higher abundance of *L. crispatus* and *L. iners* in African Americans than in other ethnicities (Roachford et al., 2022); nevertheless, most studies agree that Africans have lower *Lactobacillus-*rich microbiomes compared with White women. These racial and ethnic variations were examined recently by Xin Wei et al. (2024) and found that the nucleotide identities of these vaginal denizens found in different ethnicities varied from each other, underscoring an evolutionary mechanism that explains these observed variations (Wei et al., 2024).

In effect, the genomes of the same species had differences when isolated from different races. While they also observed similarly higher abundance of BV-associated bacteria in Black pregnant women than other races (higher alpha diversity and Shannon index), they established that the alpha diversity remained fairly constant over gestational time and across ethnicities (Wei et al., 2024). This is similar to our finding in which the differences between pregnant and non-pregnant cohorts’ vaginal microbiome variations were marginal. Notwithstanding, this study brings out certain unique findings that merit further investigation: the absence of *B. fragilis, Megasphaera* sp. type 2, and *B. breve* in pregnant women; the higher differential abundance of *G. vaginalis*, BVAB-1 and -2*, U. urealyticum, Megasphaera* sp. type 1, and *S. anginosus* in pregnant women; and the higher abundance of *S. sanguinegens* in non-pregnant women compared with pregnant women.

Albeit pregnancy had no significant effect on BV, we posit that the observed marginal variations of higher diversity and higher relative abundance in pregnant women than in non-pregnant women can be due to the hormonal changes that occur in the former. Hormonal variations in pregnancy also explain the lower relative abundance of *Lactobacillus* sp. (except *L. iners*) and anaerobes among non-pregnant women. Notably, *L. acidophilus* presented the widest variations between cohorts, suggesting that it is more sensitive to these changes than the other species and could be adopted as a biomarker. Indeed, the higher abundance of *L. crispatus* among non-pregnant and menopausal women, the absence of *L. acidophilus* in menopausal women, and the higher abundance of *L. iners* among pregnant women further show how the variations in hormones affect these species’ relative abundance through changes in the hosts estrogen-medicated metabolisms (Auriemma et al., 2021). The higher abundance of *L. crispatus* and *L. iners* was been reported previously (Borgdorff et al., 2017; Wei et al., 2024).

Although BV among non-pregnant women was more prevalent than among pregnant women, BV-negative or transitional BV samples were not obtained from pregnant women (**Figure 8**). This is a concerning observation as BV in pregnancy is associated with preterm labor, low birth weight, premature rupture of membranes, miscarriage, chorioamnionitis, and birth asphyxia (Bayigga et al., 2019; Basavaprabhu et al., 2020; Vodstrcil et al., 2021). Although the pregnancy stage of the women from whom the samples were obtained is unknown, it is known that the first-trimester vaginal microbiome is similar to a BV-positive microbiome while the 2^nd^ and 3^rd^ trimesters are similar to a normal vaginal microbiome (Kaur et al., 2020). Hence, the stage of the pregnancy and vaginal symptoms should be considered in making a final therapeutic decision regarding pregnant women when using molecular-based tests. Furthermore, the difference in the number of pregnant women (n = 53) vis-a-viz the number of non-pregnant (or unknown pregnancy status) women is large and can skew the data. This is a major limitation in our data set and should be considered when analyzing the pregnancy data.

Although BV-positive samples were found in all age groups (18 – 83 years), they were mostly prevalent among women who were between 20 and 60 years, with a higher number of BV-positive and transitional BV samples being found among women between 41 – 50 years. The species variations per age group further support this observation as the anaerobic species diversity and abundance increase from after 20 years, plateau until age 50, and begin to decline again (**Figures 12**, **13**; **Supplementary Figures S28**, **S29**). Indeed, the median age found in this study’s population is similar to what was found in Brazil (Malaguti et al., 2015) and in the USA (Watkins et al., 2024), with women above 21 years being more likely to suffer recurrence of BV (Coudray and Madhivanan, 2020; Coudray et al., 2020). Hence, the age groups found in our data concur with that of other studies, which confirms that BV is common among reproductive-age women and lower before puberty and after menopause (Auriemma et al., 2021). However, age was not significantly associated with BV occurrence in this study.

## 5 Conclusion

The new qRT-PCR assay developed by MDL is the first of its kind to quantitatively detect 22 species in the vaginal microbiome. We developed a novel classification system, the new *MDL-BV index*, which was trained on large sets of data and based on four species-based and relative abundance-based biomarkers. This multifaceted two-tier approach of using species and relative abundance provides a better diagnostic resolution for a polymicrobial infection such as BV. We are working to extend this approach to apply to other microbiome-based diagnostics to make disease diagnosis reflective of the clinical presentations. Our study is, however, limited by the absence of a clinical Nugent score or Amsel’s criteria; however, we are working to include these in the next studies that will involve the *MDL-BV index* and the 22-species qRT-PCR BV test. The clinical and socio-economic importance of this novel proprietary BV diagnostic test in obstetrics and gynecology is notable.

## Data availability statement

The original contributions presented in the study are included in the article/**Supplementary Material**. Further inquiries can be directed to the corresponding authors.

## Ethics statement

Ethics approval was not required for this study as the samples used were leftover, discarded samples provided by healthcare providers and were anonymized, posing no risk of identifying individual patients. According to the Common Rule (45 CFR 46) and HIPAA (45 CFR 164.514(b)(2)), research involving anonymized data does not constitute human subjects research and thus does not require IRB approval. Written informed consent was not obtained as the study used leftover, discarded samples, and the study did not involve direct interaction with patients.

## Author contributions

AO: Conceptualization, Data curation, Formal analysis, Investigation, Methodology, Project administration, Software, Supervision, Validation, Visualization, Writing – original draft, Writing – review & editing. RH: Conceptualization, Data curation, Formal analysis, Investigation, Methodology, Project administration, Software, Supervision, Validation, Visualization, Writing – original draft, Writing – review & editing. JT: Investigation, Methodology, Supervision, Writing – review & editing. SF: Supervision, Validation, Writing – review & editing. EM: Funding acquisition, Resources, Supervision, Validation, Visualization, Writing – review & editing. MA: Funding acquisition, Project administration, Resources, Supervision, Validation, Visualization, Writing – review & editing. JO: Conceptualization, Data curation, Formal analysis, Investigation, Methodology, Project administration, Software, Supervision, Validation, Visualization, Writing – original draft, Writing – review & editing.

## Supplementary material

Supplementary Table 1 Raw demographic and qPCR data showing the demographics of the women from whom the samples were obtained and the qPCR gDNA concentrations of each species within the 946 samples.

Supplementary Table 2 Chi-square and T-test statistical data showing the significant and non-significant associations between *Lactobacillus* sp. and the other non-*Lactobacillus* species.

Supplementary Table 3 BV status classification data generated by the Python algorithm trained on the MDL-BV index ranges. The relative abundance of each group of biomarkers are summed up and shown under their respective groups. The ratio of these relative abundances are used to infer the BV status of the sample.

Supplementary Figures 1–22 Concentration distribution of each of the 22 bacterial species tested by the qPCR test across all the samples. Both boxplot and histograms charts are shown for each species.

Supplementary Figure 23 A correlation matrix showing the correlation between the individual species found within the samples. Different color codes are used to show positive and negative correlations.

Supplementary Figure 24 A correlation heatmap showing the correlation between age, pregnancy, race, and BV status.
