# Supplementary material for: Molecular characterization of vaginal microbiota using a new 22-species qRT-PCR test to achieve a relative-abundance and species-based diagnosis of bacterial vaginosis
Source: Front Cell Infect Microbiol. 2024 Jun 28;14:1409774. doi: 10.3389/fcimb.2024.1409774 (PMC11239351; doi:10.3389/fcimb.2024.1409774)

Supplemental Figures

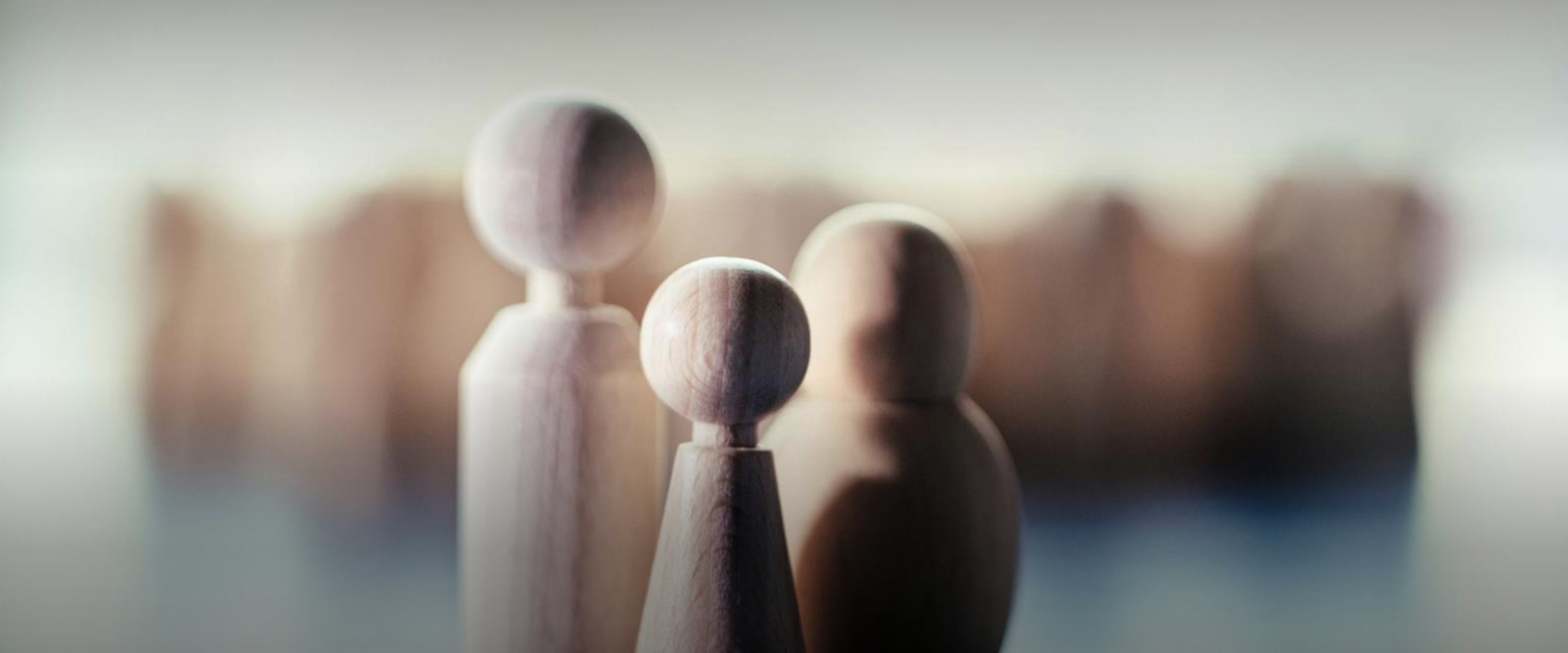

Fig. S1. Concentration distribution of *Bacteroides fragilis* across the samples. On the left, a Box and Whisker Plot displays the distribution's median, quartiles, and potential outliers. On the right, a Histogram with a Kernel Density Estimate (KDE) overlay shows the concentration distribution across all samples, offering a view of the data's spread and density.

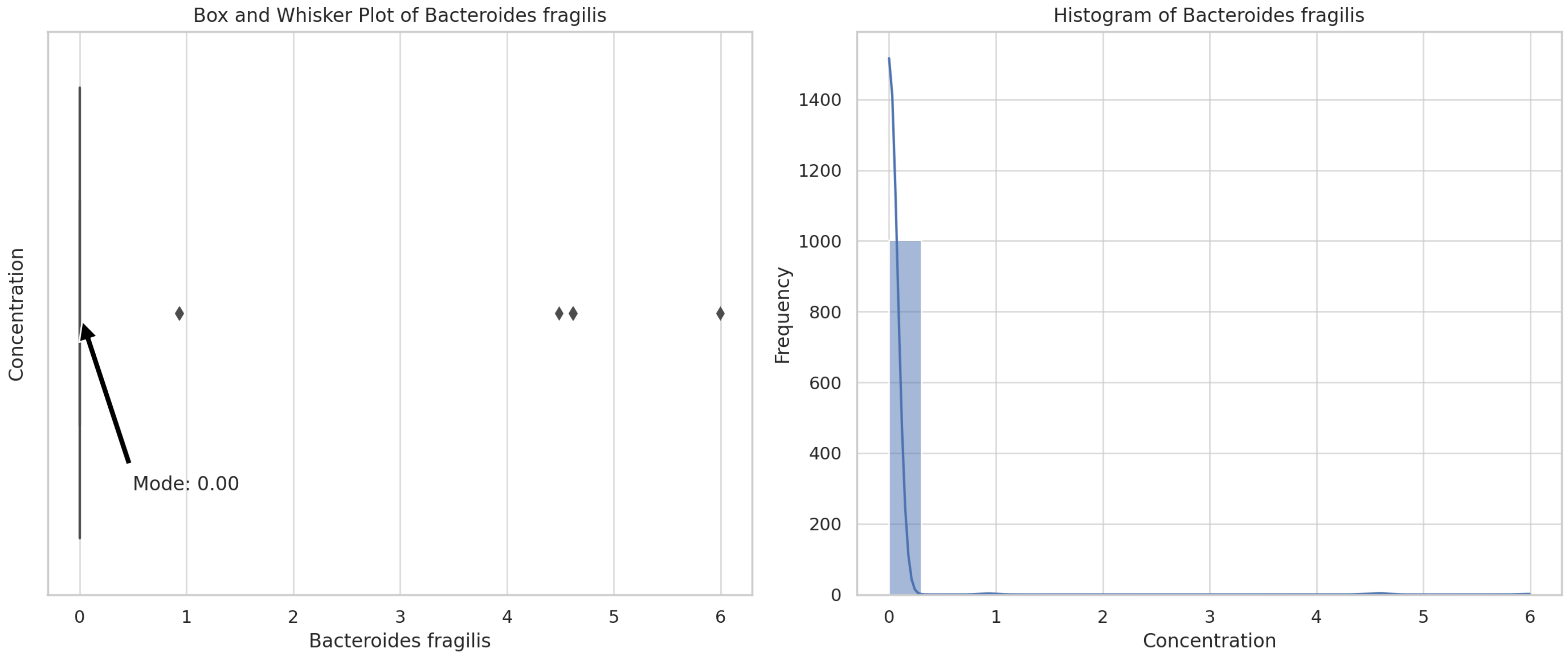

Fig. S2. Concentration distribution of *Bifidobacterium breve* across the samples. On the left, a Box and Whisker Plot displays the distribution's median, quartiles, and potential outliers. On the right, a Histogram with a Kernel Density Estimate (KDE) overlay shows the concentration distribution across all samples, offering a view of the data's spread and density.

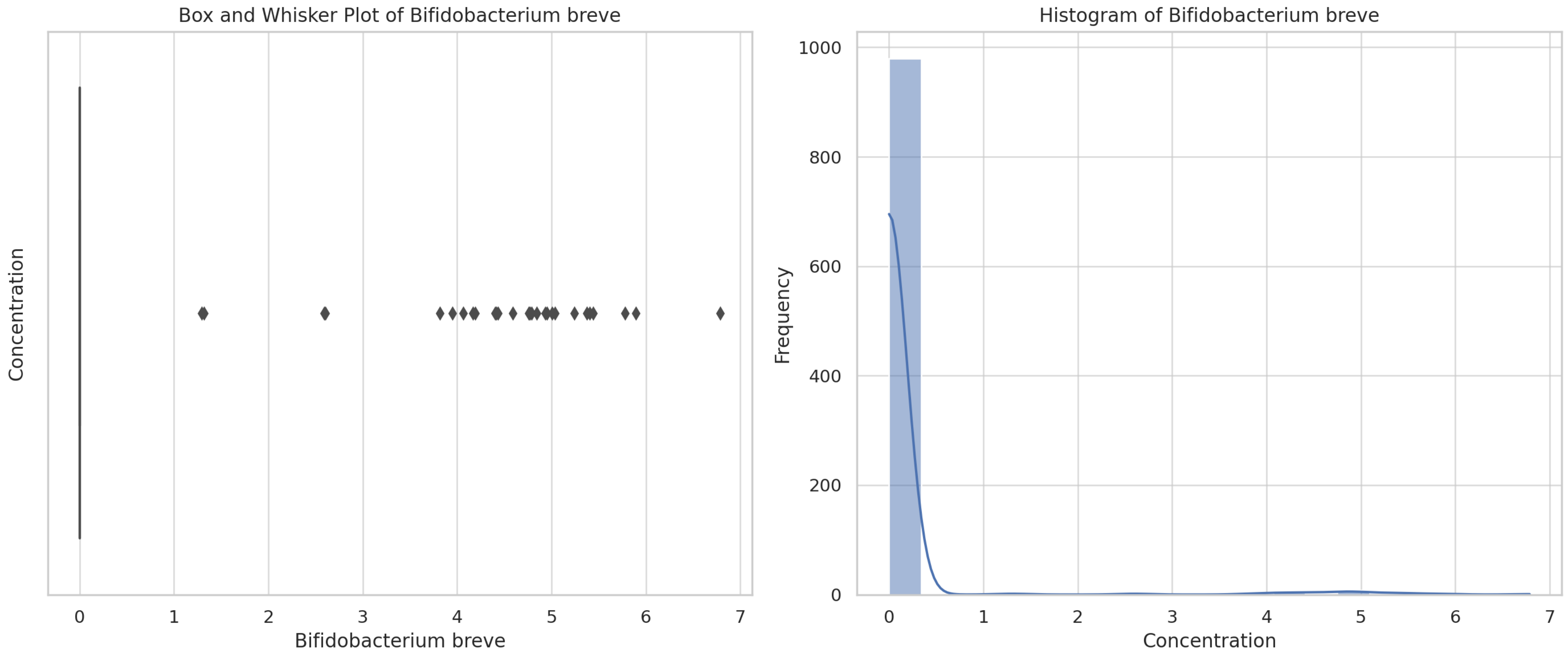

Fig. S3. Concentration distribution of *Mobiluncus curtisii* across the samples. On the left, a Box and Whisker Plot displays the distribution's median, quartiles, and potential outliers. On the right, a Histogram with a Kernel Density Estimate (KDE) overlay shows the concentration distribution across all samples, offering a view of the data's spread and density.

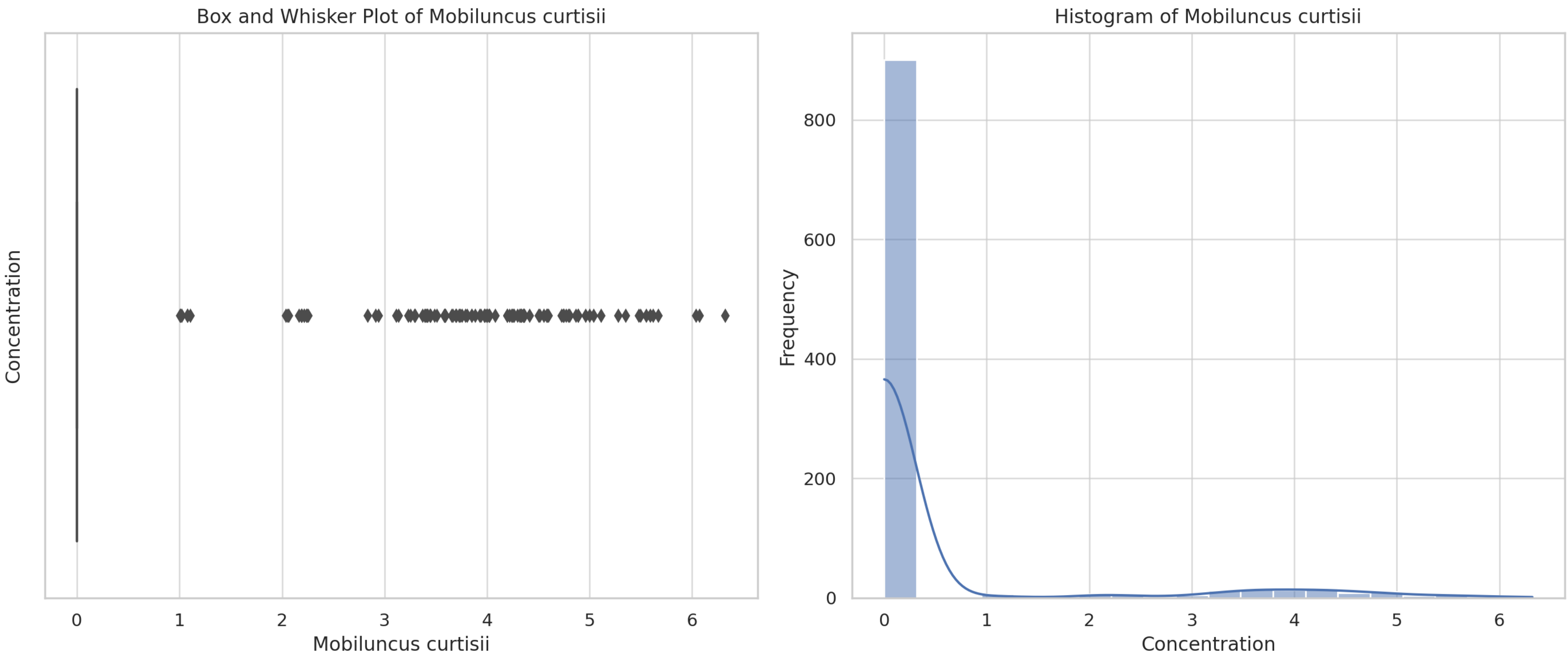

Fig. S4. Concentration distribution of *Mobiluncus mulieris* across the samples. On the left, a Box and Whisker Plot displays the distribution's median, quartiles, and potential outliers. On the right, a Histogram with a Kernel Density Estimate (KDE) overlay shows the concentration distribution across all samples, offering a view of the data's spread and density.

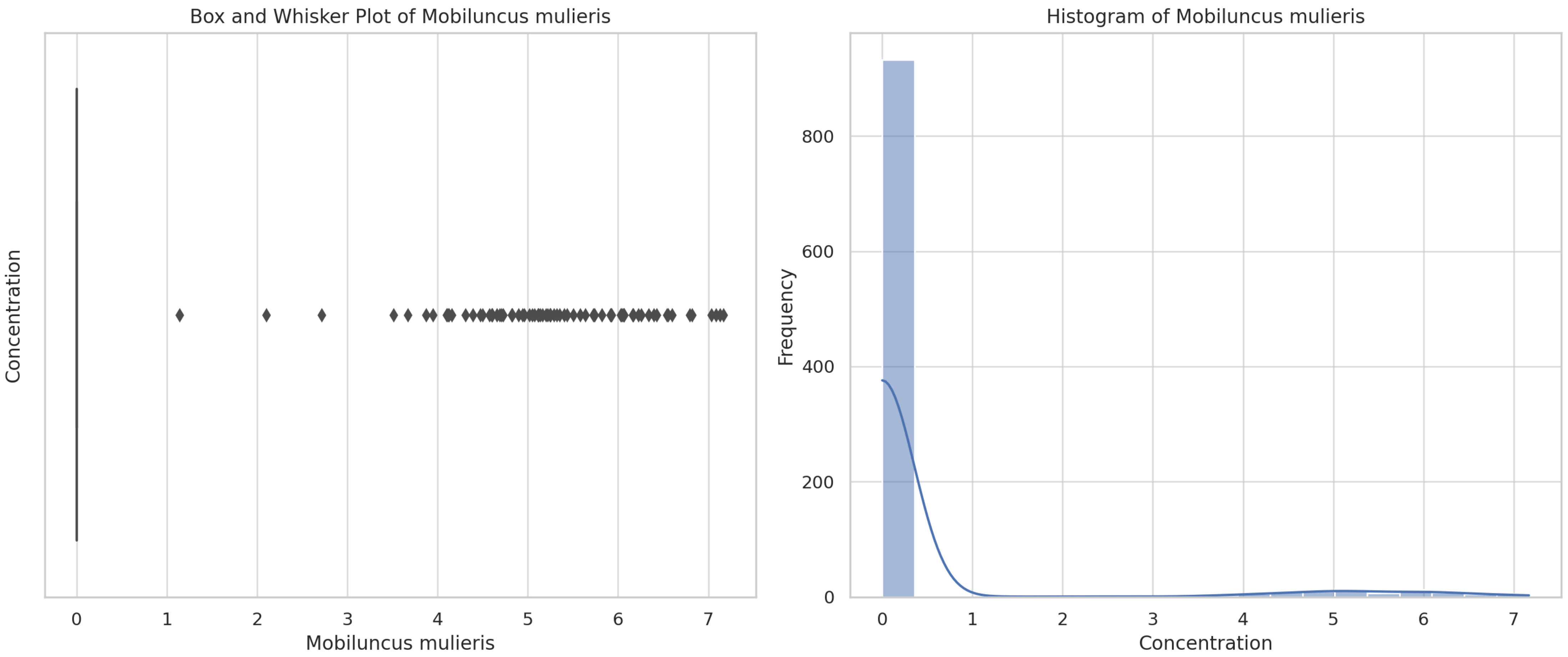

Fig. S5. Concentration distribution of *Streptococcus anginosus* across the samples. On the left, a Box and Whisker Plot displays the distribution's median, quartiles, and potential outliers. On the right, a Histogram with a Kernel Density Estimate (KDE) overlay shows the concentration distribution across all samples, offering a view of the data's spread and density.

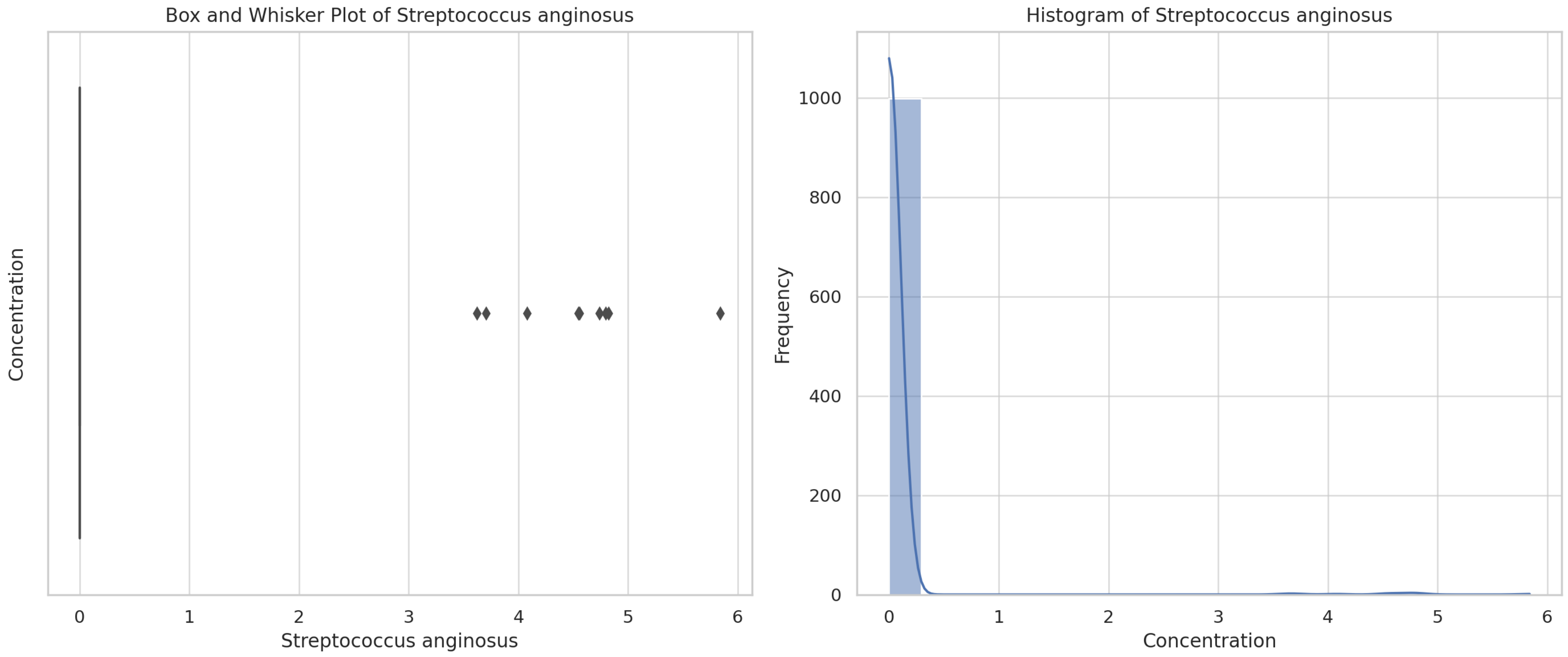

Fig. S6. Concentration distribution of *Sneathia sanguinegens* across the samples. On the left, a Box and Whisker Plot displays the distribution's median, quartiles, and potential outliers. On the right, a Histogram with a Kernel Density Estimate (KDE) overlay shows the concentration distribution across all samples, offering a view of the data's spread and density.

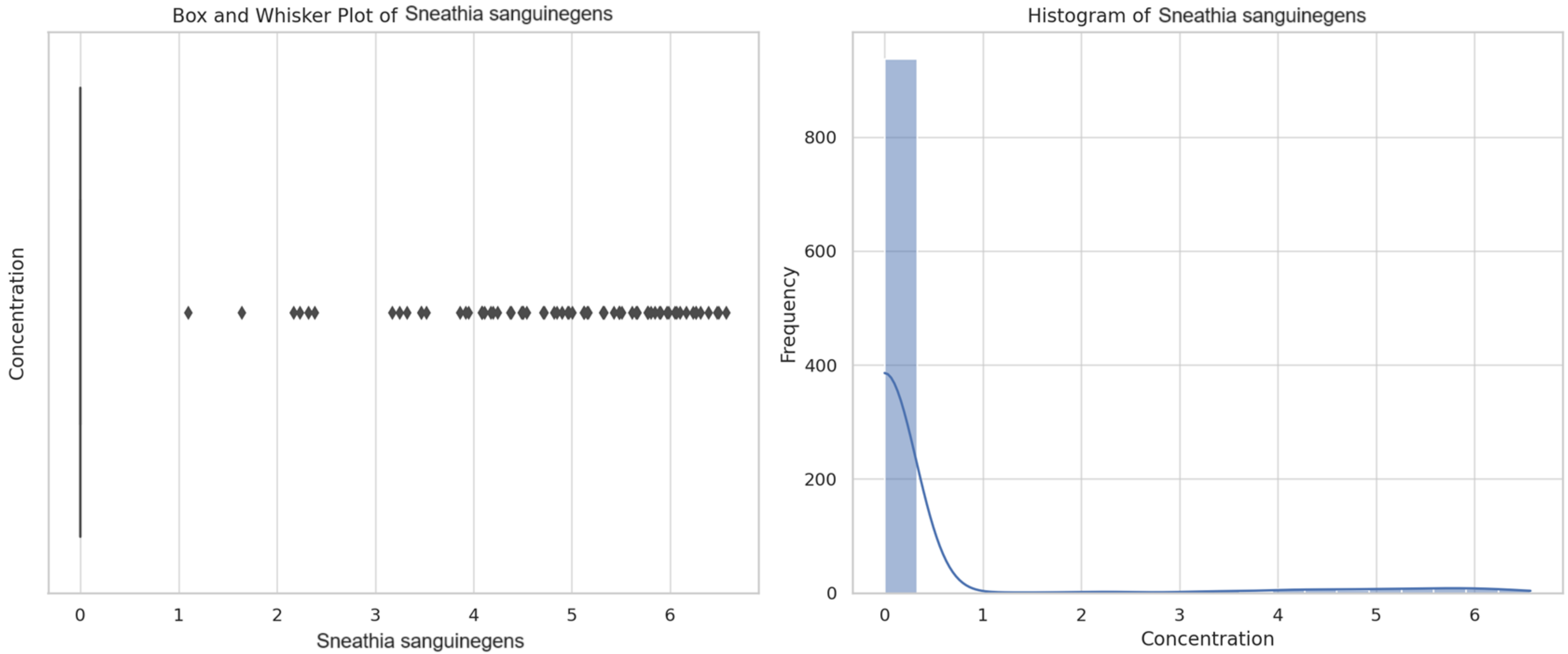

Fig. S7. Concentration distribution of *Ureaplasma urealyticum* across the samples. On the left, a Box and Whisker Plot displays the distribution's median, quartiles, and potential outliers. On the right, a Histogram with a Kernel Density Estimate (KDE) overlay shows the concentration distribution across all samples, offering a view of the data's spread and density.

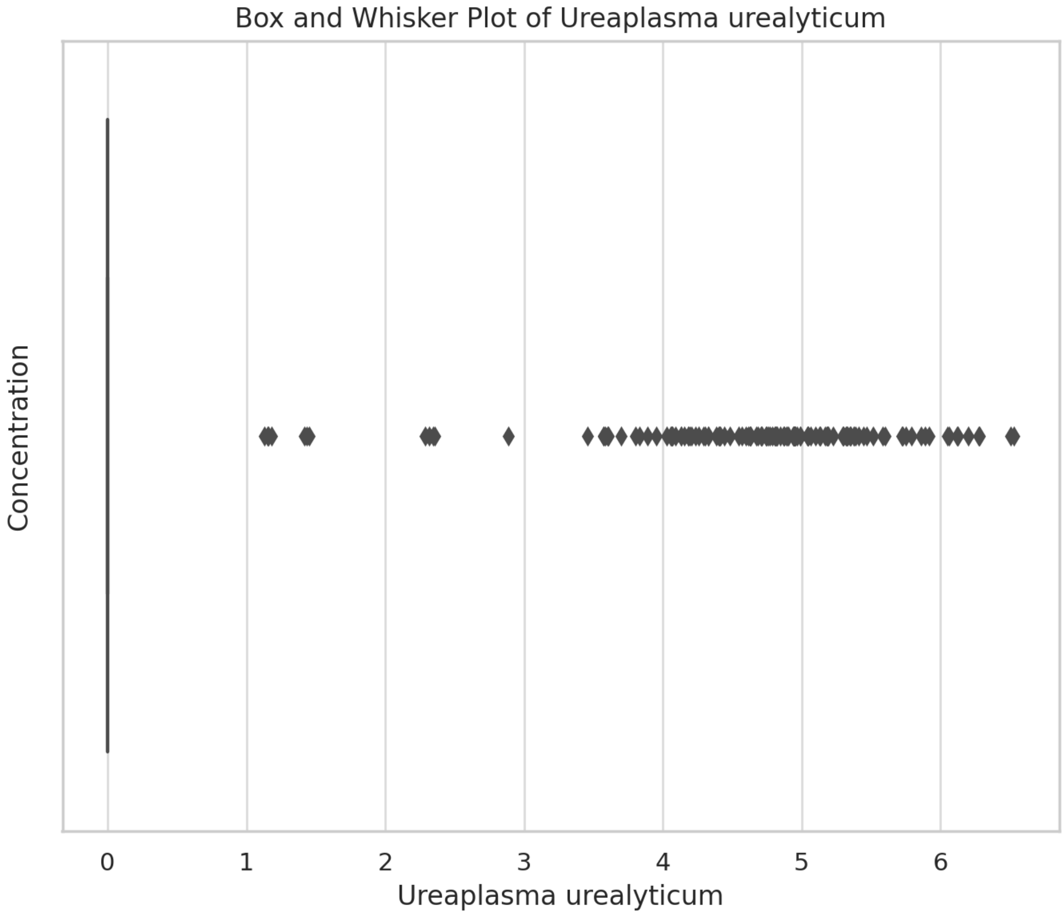

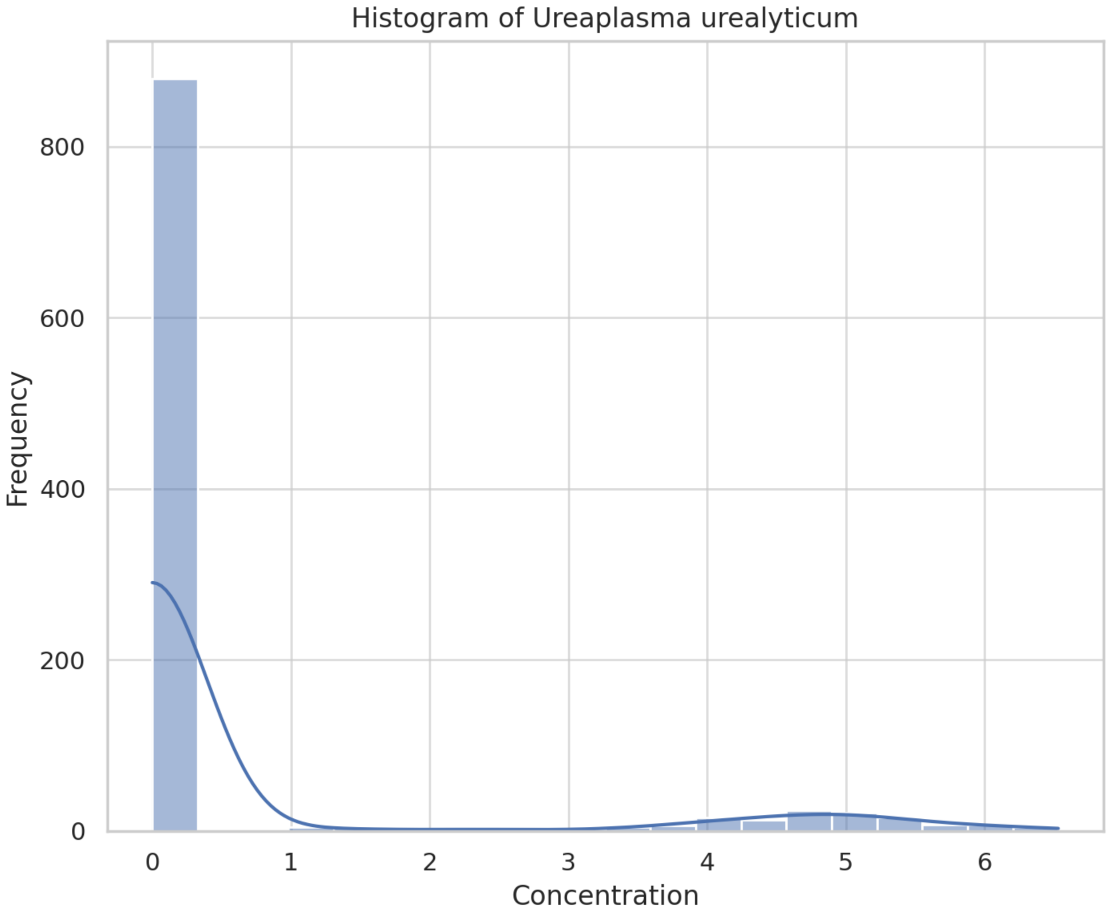

Fig. S8. Concentration distribution of *Mycoplasma hominis* across the samples. On the left, a Box and Whisker Plot displays the distribution's median, quartiles, and potential outliers. On the right, a Histogram with a Kernel Density Estimate (KDE) overlay shows the concentration distribution across all samples, offering a view of the data's spread and density.

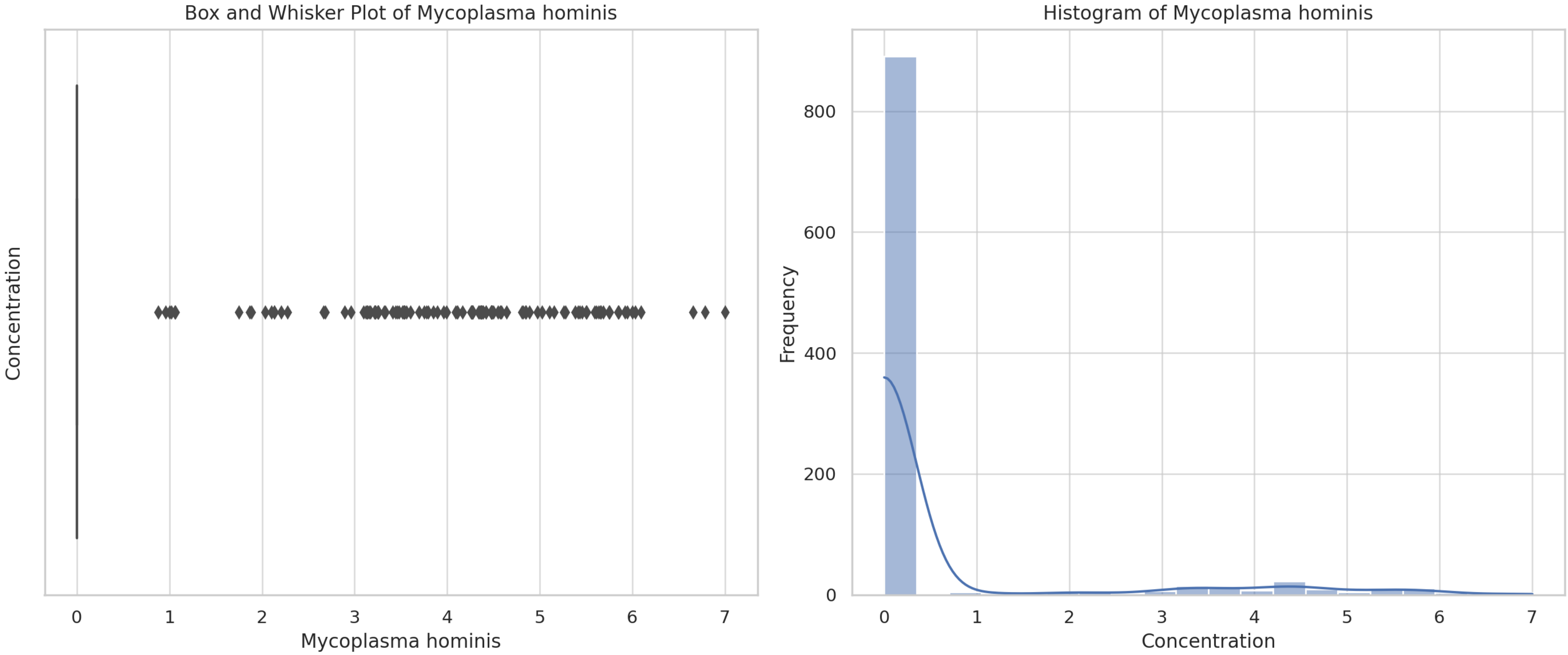

Fig. S9. Concentration distribution of *Mycoplasma genitalium* across the samples. On the left, a Box and Whisker Plot displays the distribution's median, quartiles, and potential outliers. On the right, a Histogram with a Kernel Density Estimate (KDE) overlay shows the concentration distribution across all samples, offering a view of the data's spread and density.

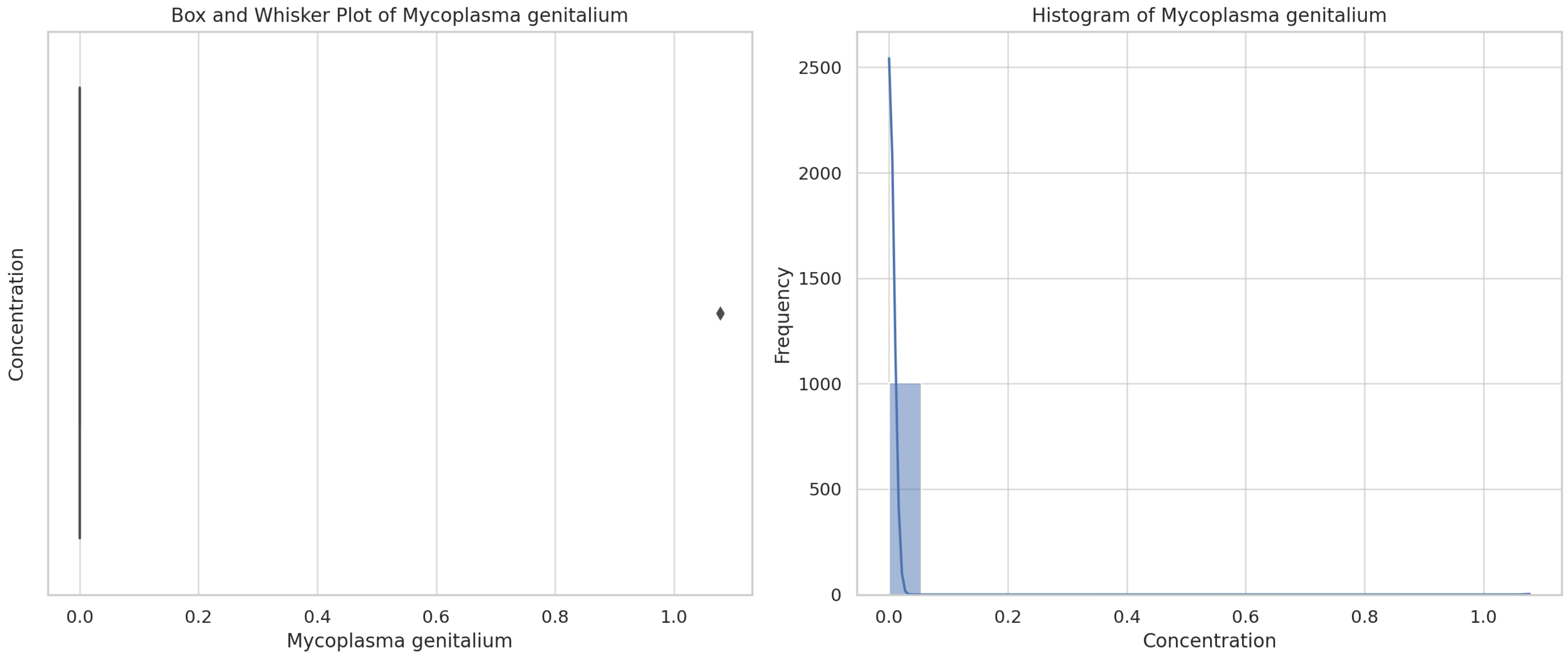

Fig. S10. Concentration distribution of *Megasphaera sp. type 1* across the samples. On the left, a Box and Whisker Plot displays the distribution's median, quartiles, and potential outliers. On the right, a Histogram with a Kernel Density Estimate (KDE) overlay shows the concentration distribution across all samples, offering a view of the data's spread and density.

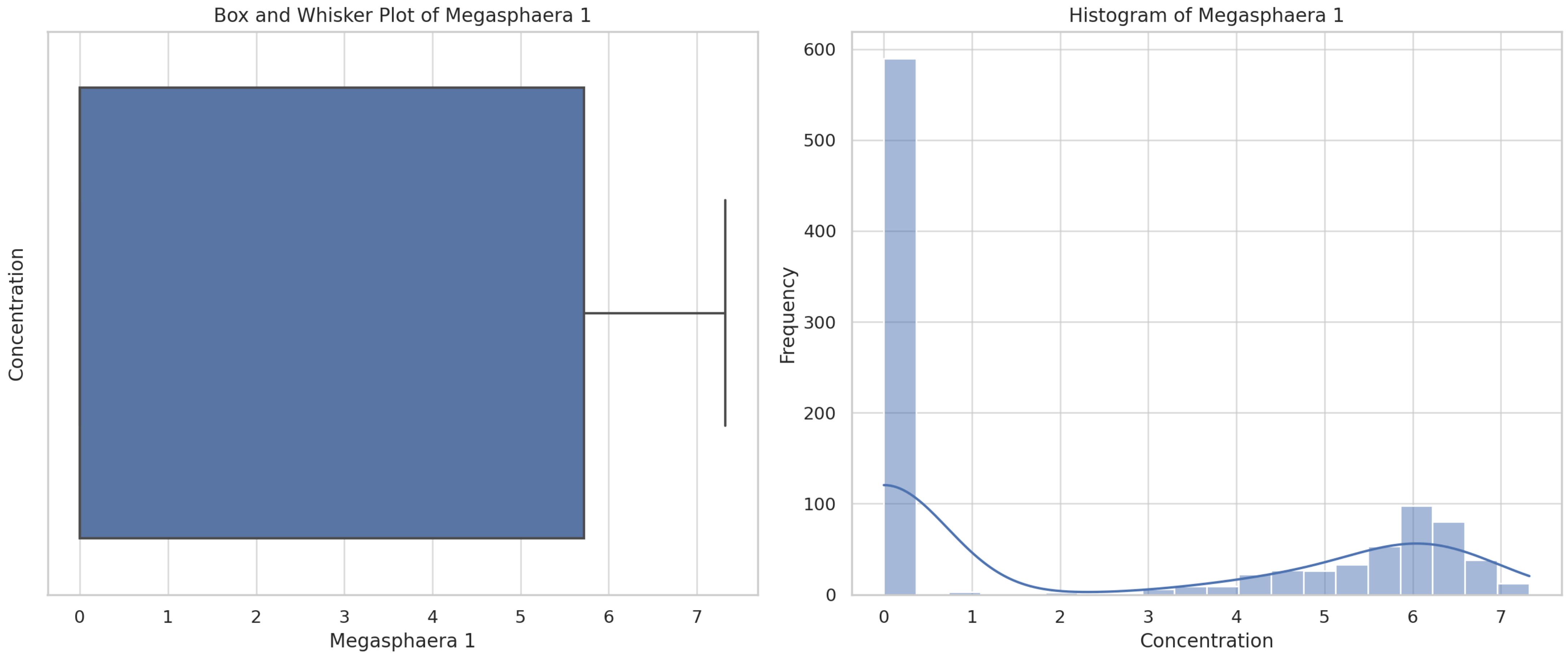

Fig. S11. Concentration distribution of *Megasphaera sp. type 2* across the samples. On the left, a Box and Whisker Plot displays the distribution's median, quartiles, and potential outliers. On the right, a Histogram with a Kernel Density Estimate (KDE) overlay shows the concentration distribution across all samples, offering a view of the data's spread and density.

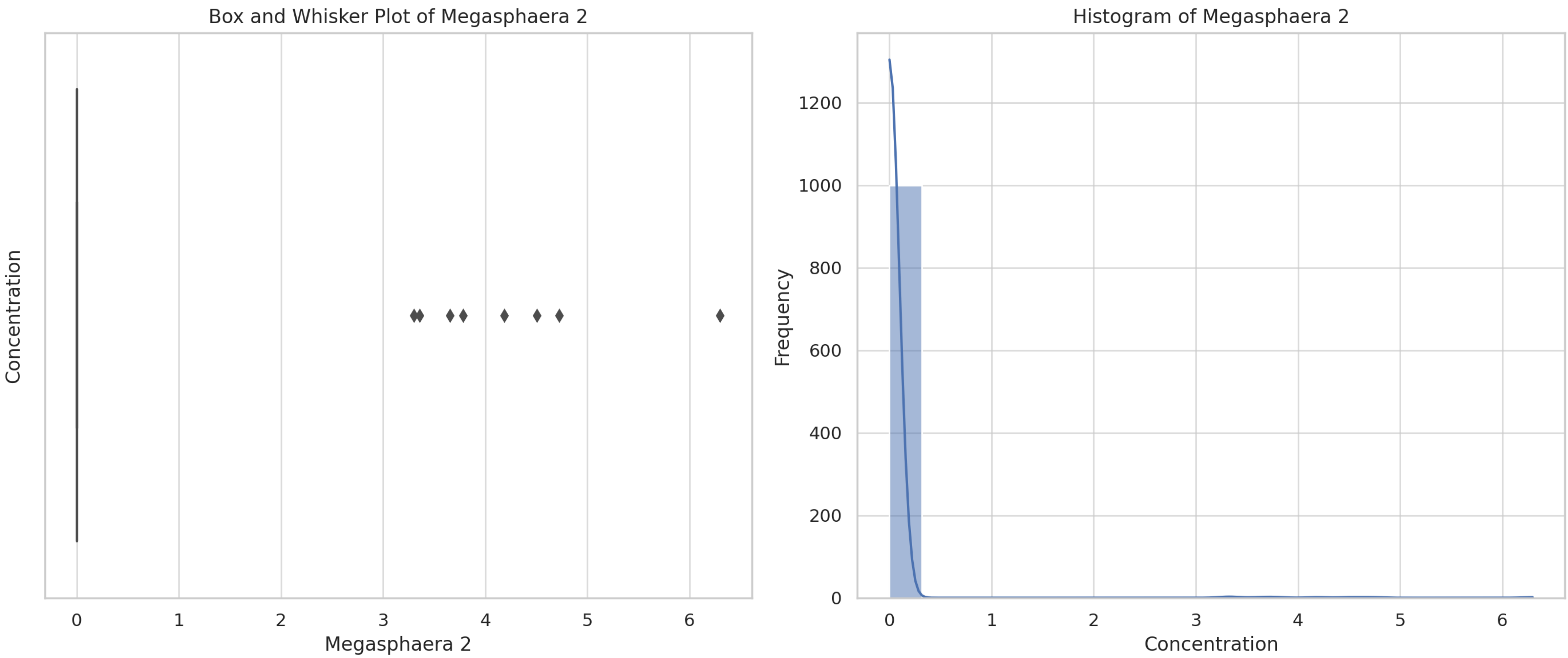

Fig. S12. Concentration distribution of *Gardnerella vaginalis* across the samples. On the left, a Box and Whisker Plot displays the distribution's median, quartiles, and potential outliers. On the right, a Histogram with a Kernel Density Estimate (KDE) overlay shows the concentration distribution across all samples, offering a view of the data's spread and density.

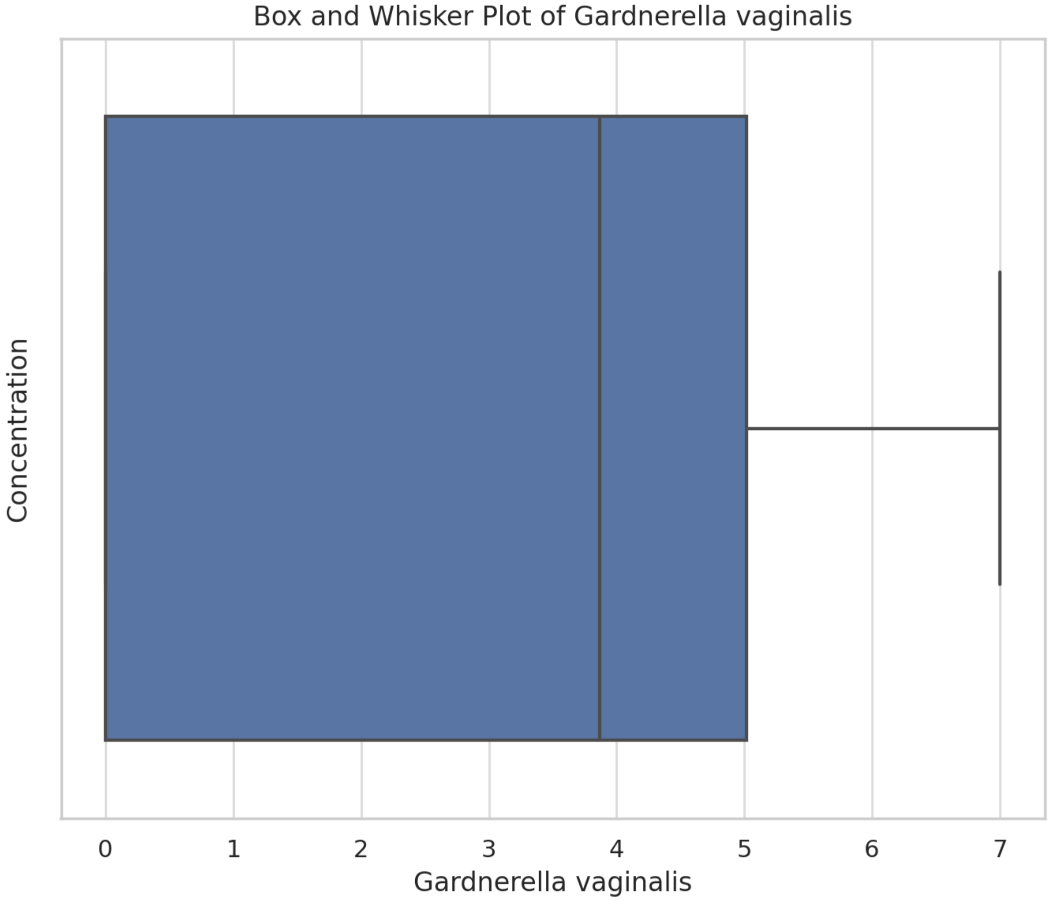

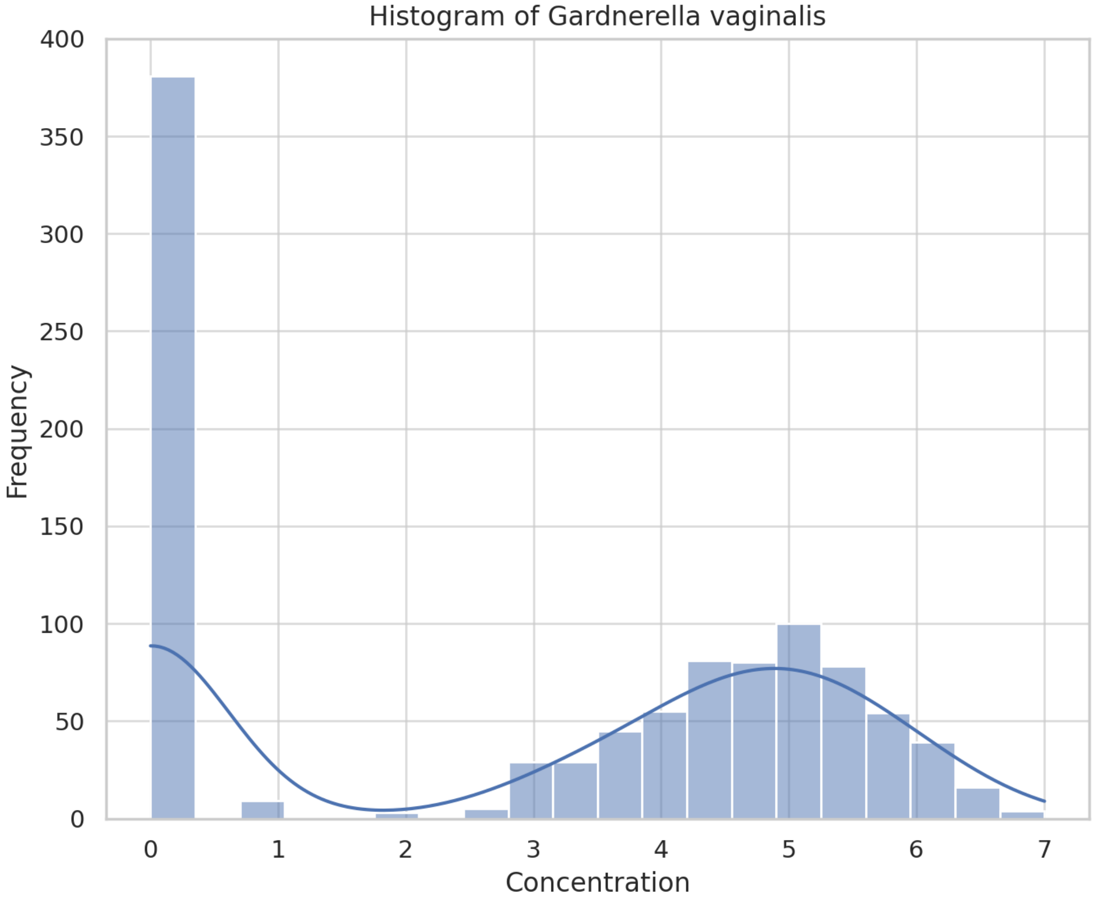

Fig. S13. Concentration distribution of *Atopobium (Fannyhesae) vaginae* across the samples. On the left, a Box and Whisker Plot displays the distribution's median, quartiles, and potential outliers. On the right, a Histogram with a Kernel Density Estimate (KDE) overlay shows the concentration distribution across all samples, offering a view of the data's spread and density.

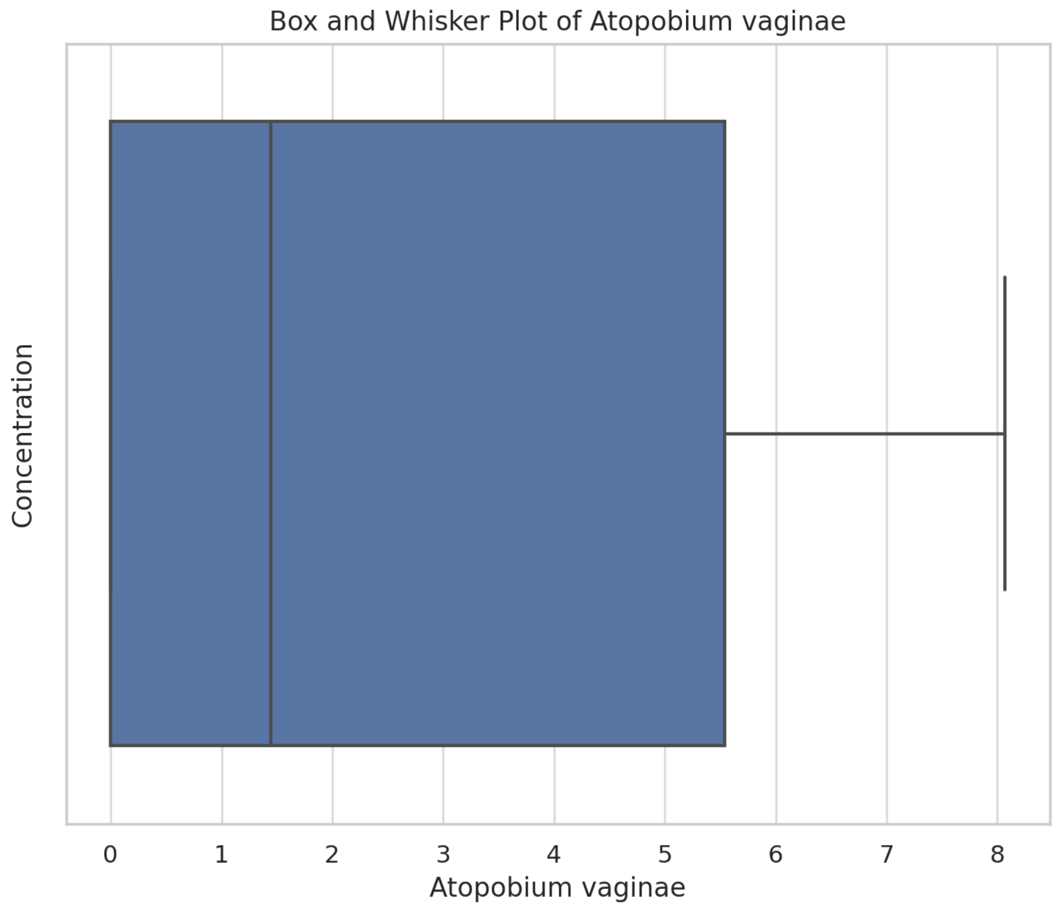

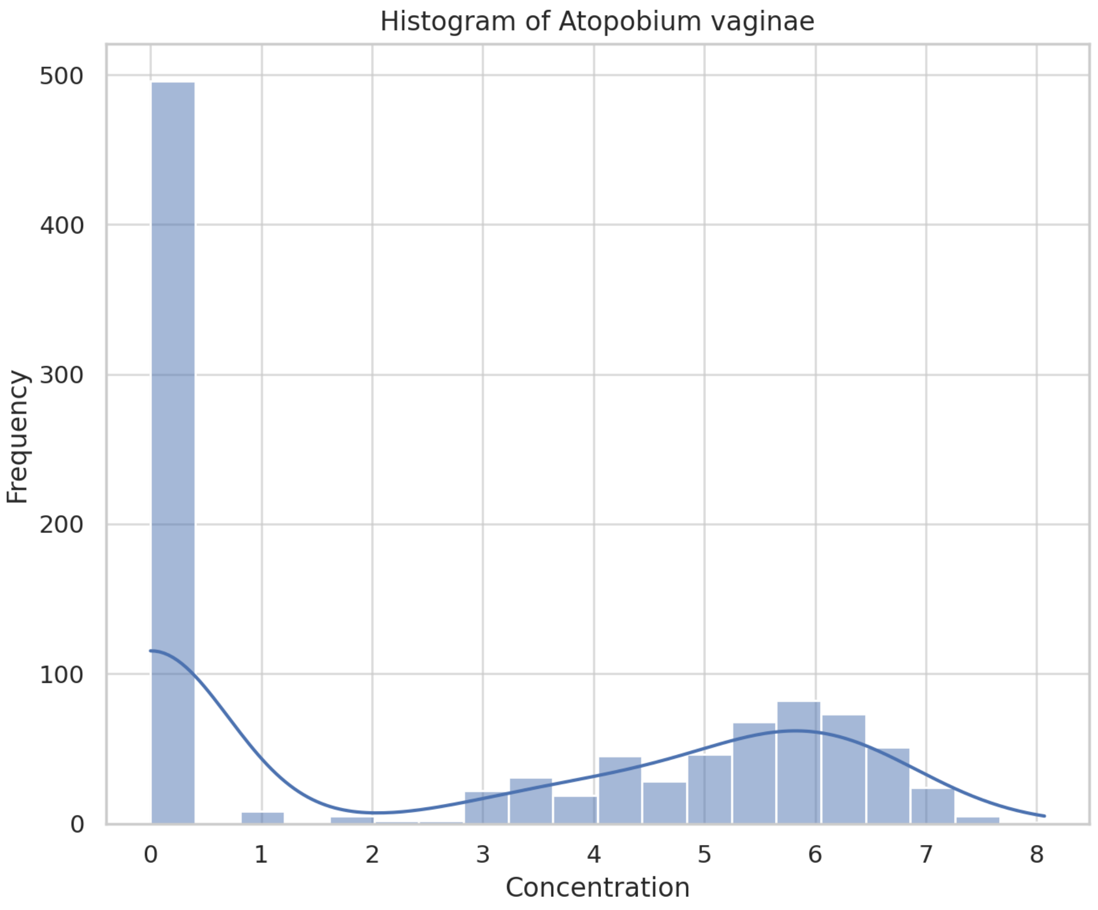

Fig. S14. Concentration distribution of *Prevotella bivia* across the samples. On the left, a Box and Whisker Plot displays the distribution's median, quartiles, and potential outliers. On the right, a Histogram with a Kernel Density Estimate (KDE) overlay shows the concentration distribution across all samples, offering a view of the data's spread and density.

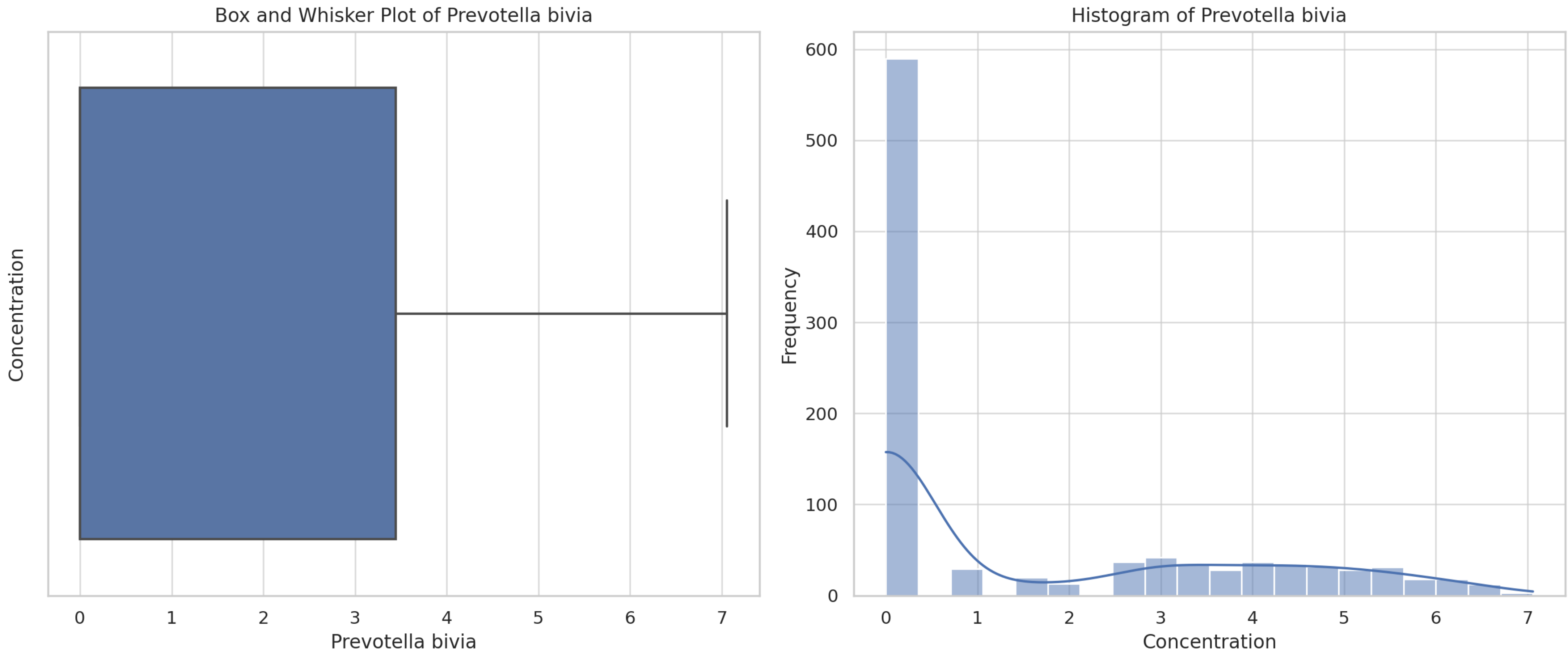

Fig. S15. Concentration distribution of *BVAB-1* across the samples. On the left, a Box and Whisker Plot displays the distribution's median, quartiles, and potential outliers. On the right, a Histogram with a Kernel Density Estimate (KDE) overlay shows the concentration distribution across all samples, offering a view of the data's spread and density.

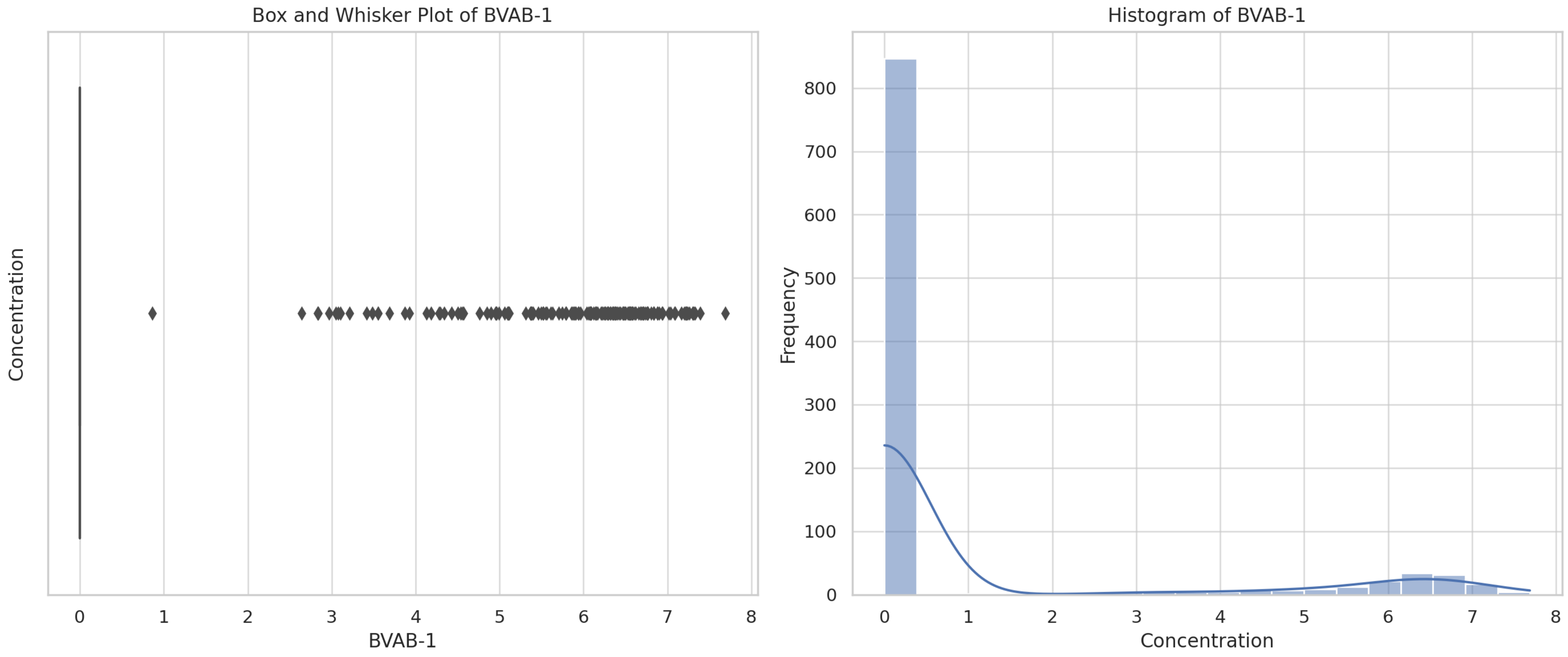

Fig. S16. Concentration distribution of *BVAB-2* across the samples. On the left, a Box and Whisker Plot displays the distribution's median, quartiles, and potential outliers. On the right, a Histogram with a Kernel Density Estimate (KDE) overlay shows the concentration distribution across all samples, offering a view of the data's spread and density.

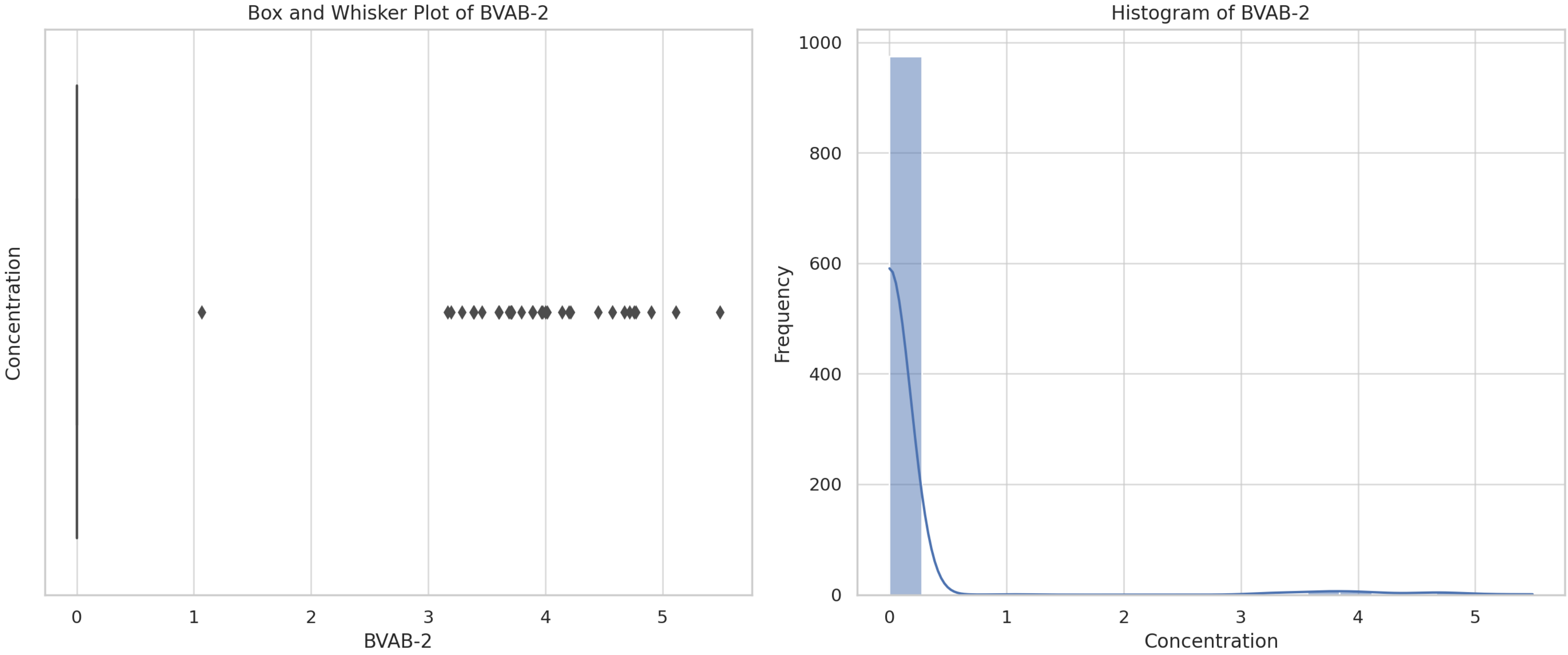

Fig. S17. Concentration distribution of *BVAB-3* across the samples. On the left, a Box and Whisker Plot displays the distribution's median, quartiles, and potential outliers. On the right, a Histogram with a Kernel Density Estimate (KDE) overlay shows the concentration distribution across all samples, offering a view of the data's spread and density.

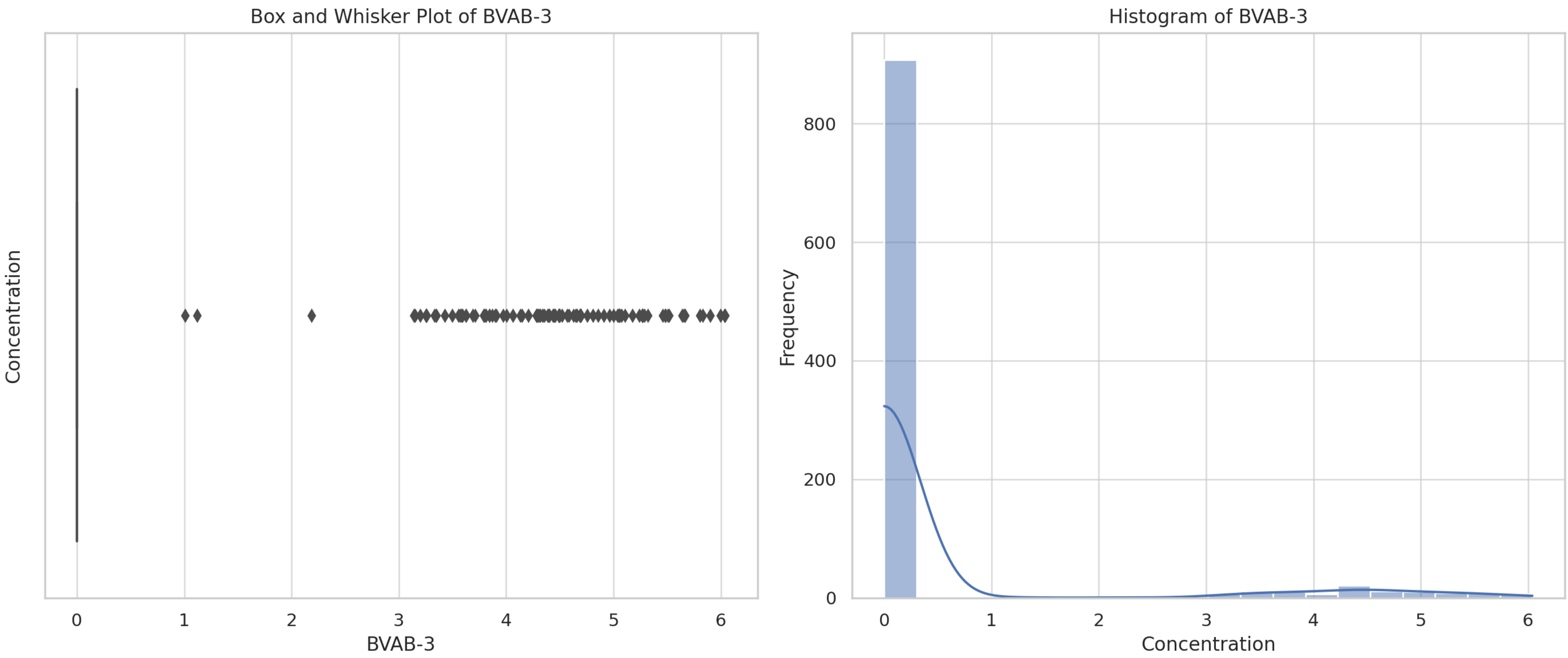

Fig. S18. Concentration distribution of *Lactobacillus gasseri* across the samples. On the left, a Box and Whisker Plot displays the distribution's median, quartiles, and potential outliers. On the right, a Histogram with a Kernel Density Estimate (KDE) overlay shows the concentration distribution across all samples, offering a view of the data's spread and density.

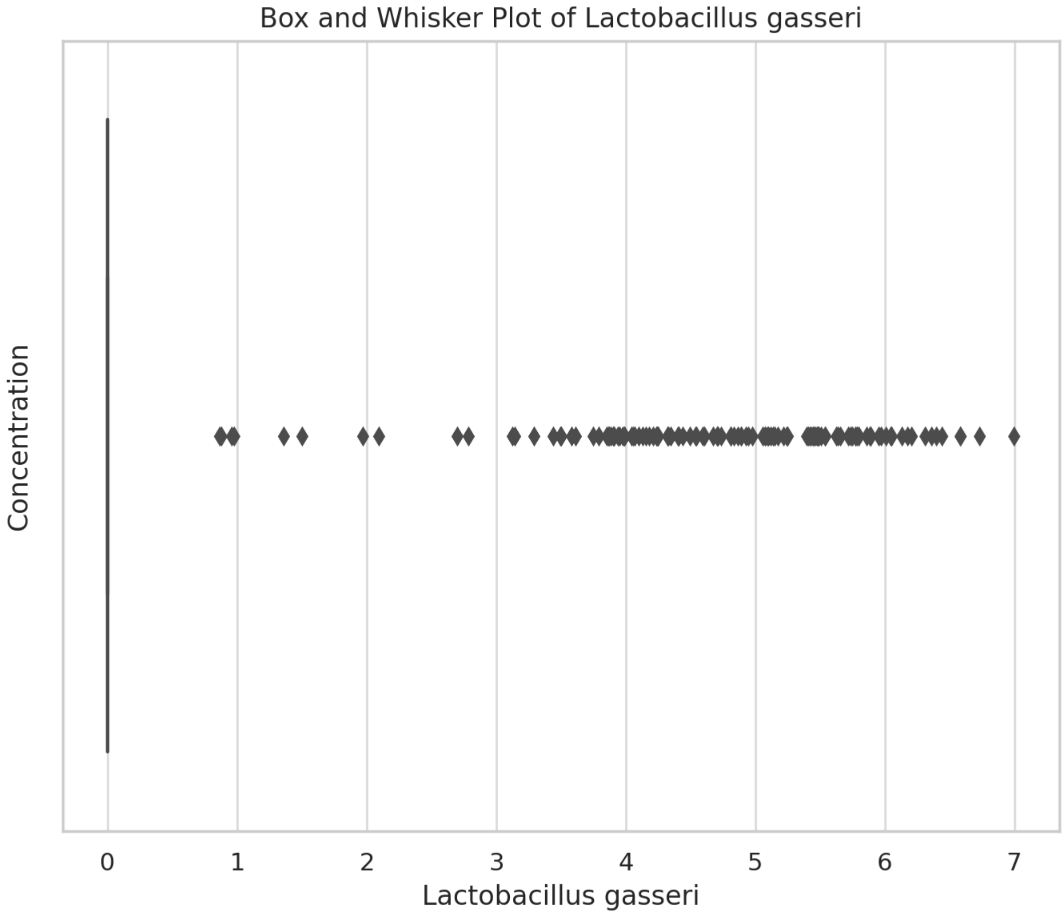

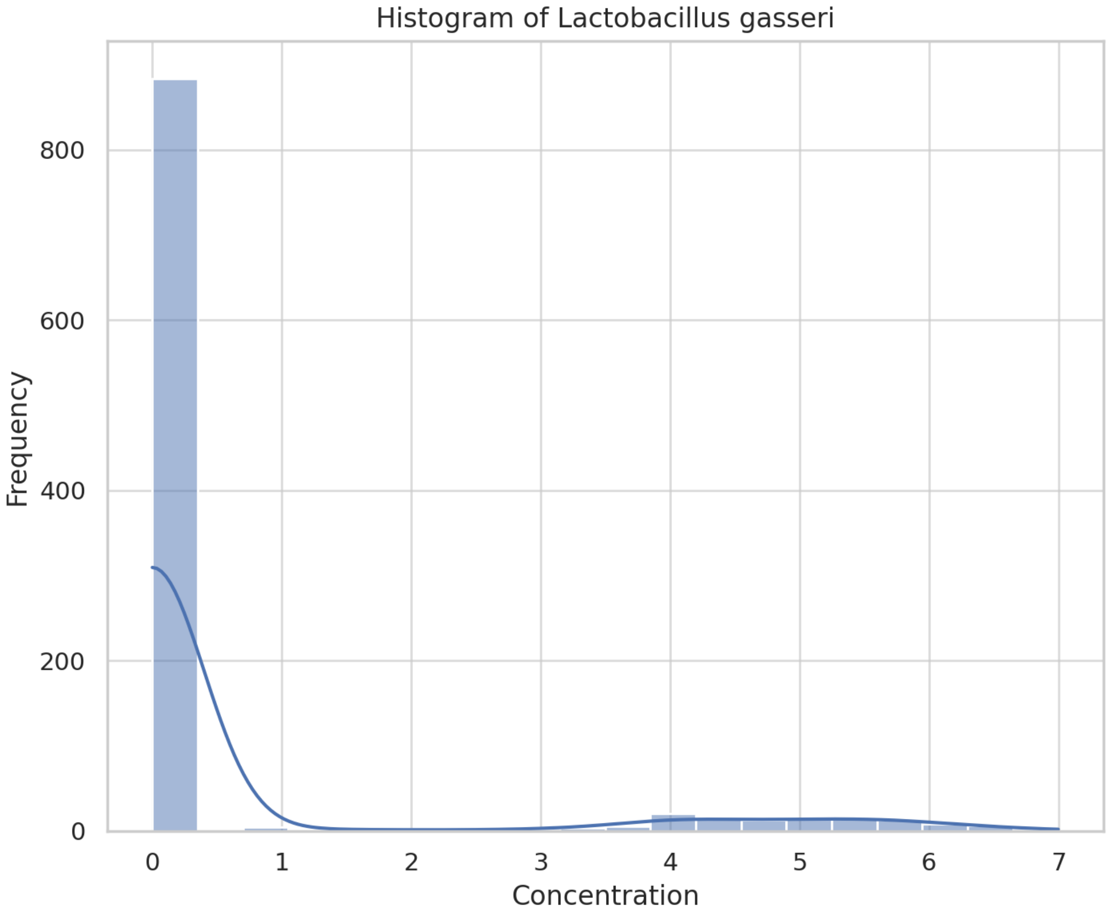

Fig. S19. Concentration distribution of *Lactobacillus iners* across the samples. On the left, a Box and Whisker Plot displays the distribution's median, quartiles, and potential outliers. On the right, a Histogram with a Kernel Density Estimate (KDE) overlay shows the concentration distribution across all samples, offering a view of the data's spread and density.

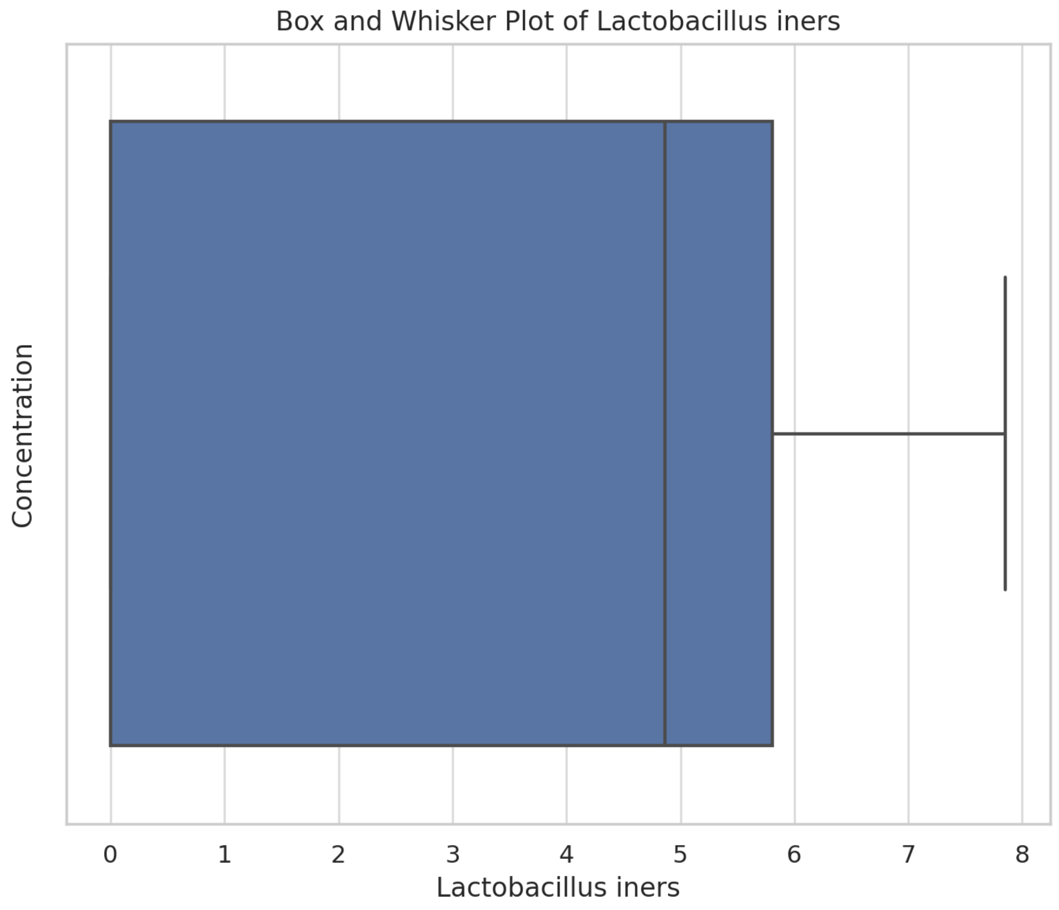

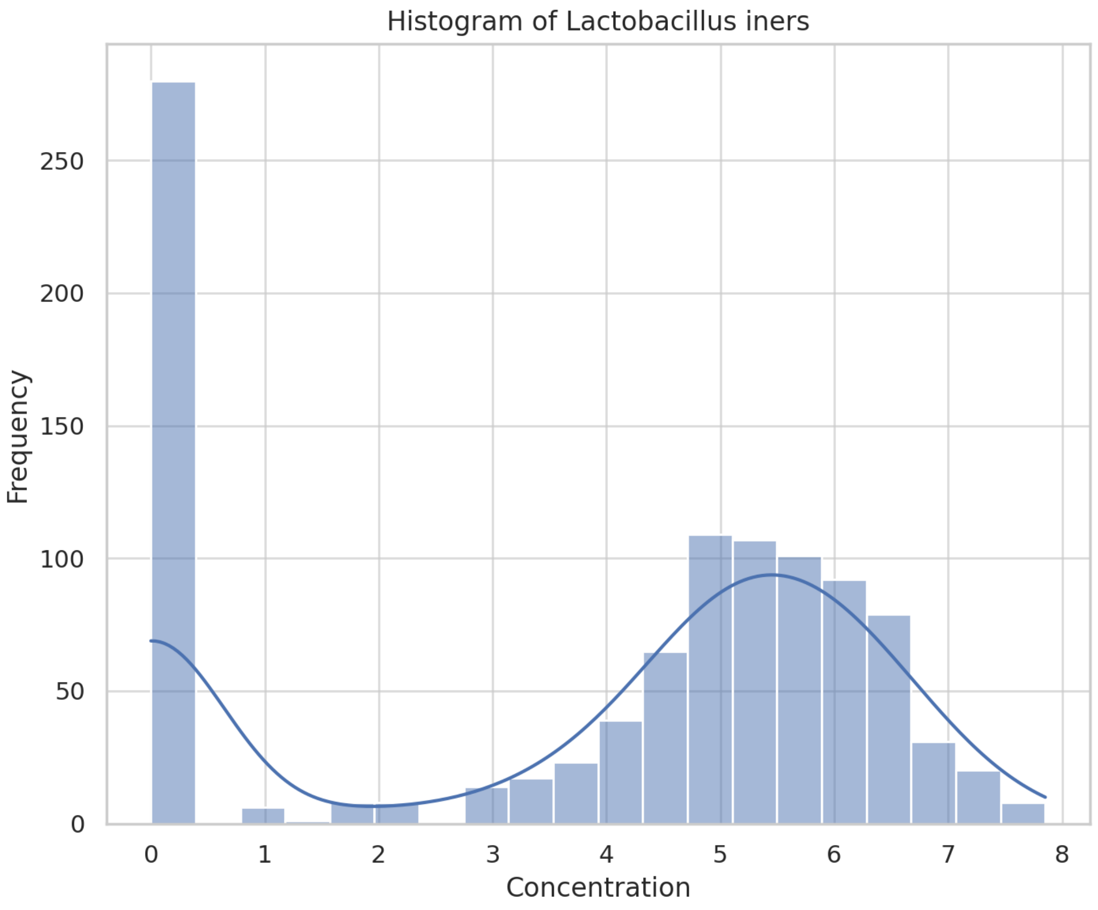

Fig. S20. Concentration distribution of *Lactobacillus crispatus* across the samples. On the left, a Box and Whisker Plot displays the distribution's median, quartiles, and potential outliers. On the right, a Histogram with a Kernel Density Estimate (KDE) overlay shows the concentration distribution across all samples, offering a view of the data's spread and density.

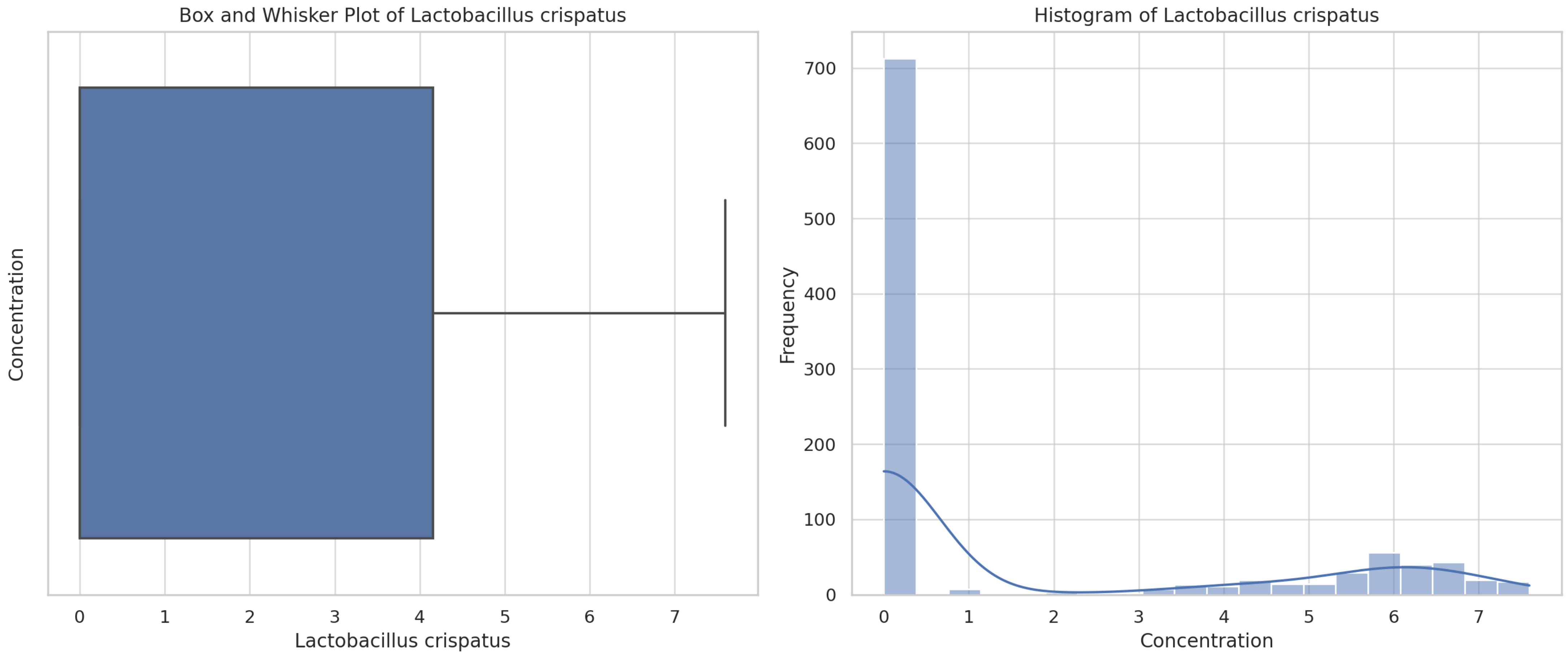

Fig. S21. Concentration distribution of *Lactobacillus jensenii* across the samples. On the left, a Box and Whisker Plot displays the distribution's median, quartiles, and potential outliers. On the right, a Histogram with a Kernel Density Estimate (KDE) overlay shows the concentration distribution across all samples, offering a view of the data's spread and density.

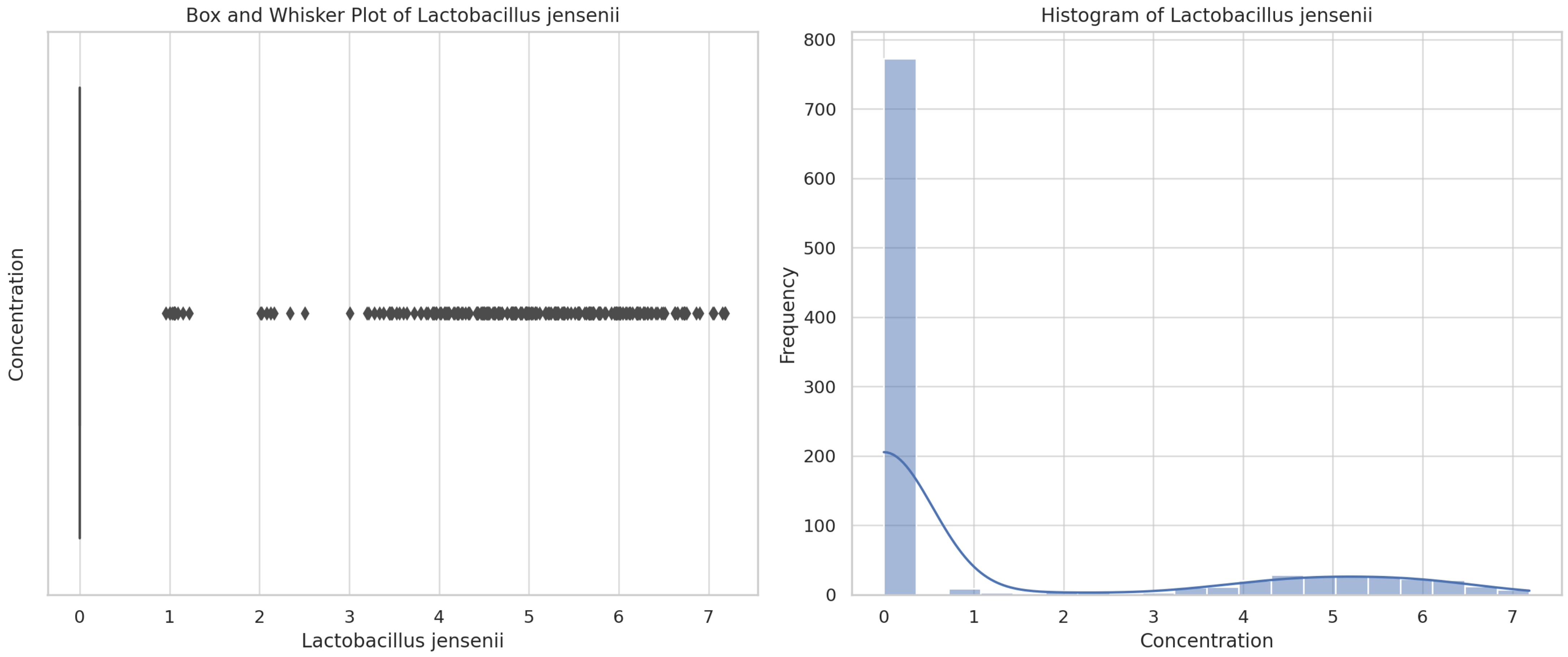

Fig. S22. Concentration distribution of *Lactobacillus acidophilus* across the samples. On the left, a Box and Whisker Plot displays the distribution's median, quartiles, and potential outliers. On the right, a Histogram with a Kernel Density Estimate (KDE) overlay shows the concentration distribution across all samples, offering a view of the data's spread and density.

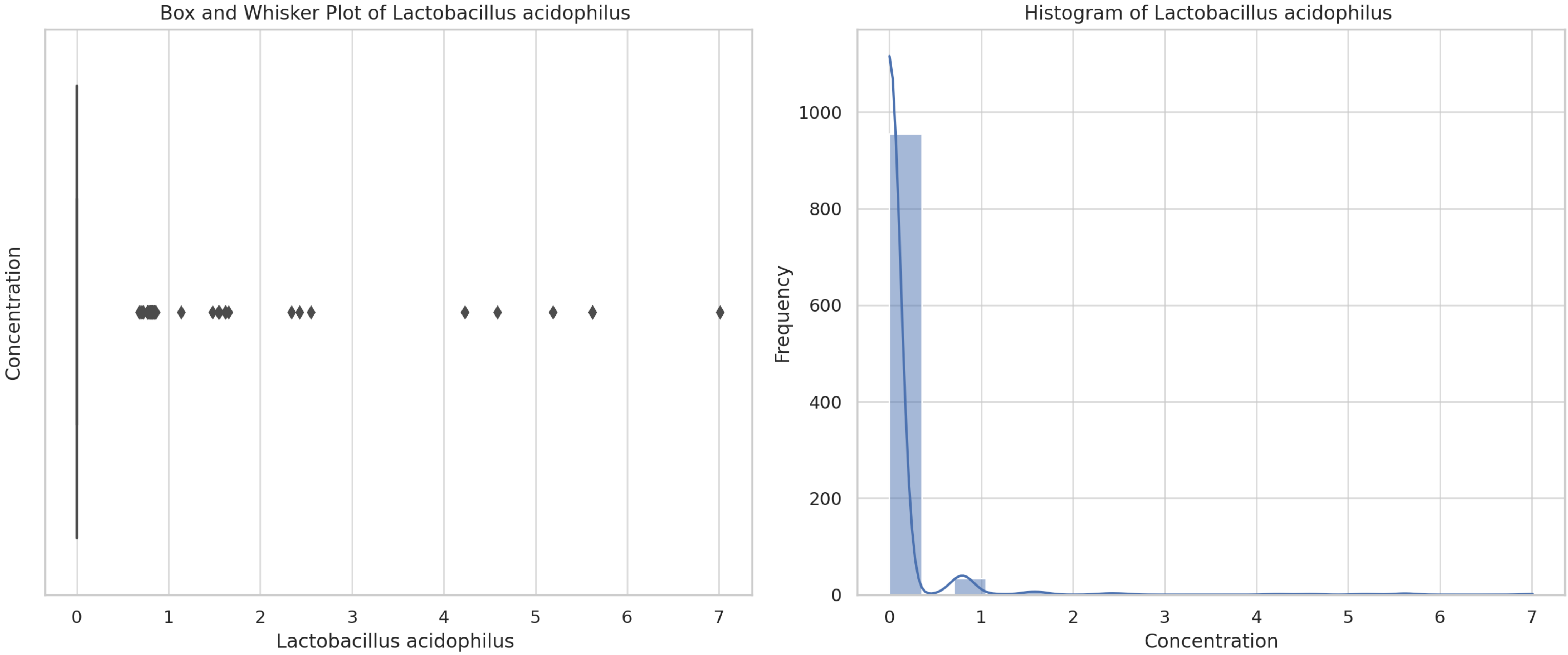

Figure S23. Correlation matrix showing the pairwise association of the species to each other using Pearson's correlation co-efficient. The heatmap above displays the correlation matrix of microbial species across the vaginal samples. Each cell in the matrix shows the Pearson correlation coefficient between pairs of species, with the color scale ranging from -1 (perfect negative correlation, shown in blue) to 1 (perfect positive correlation, shown in red). Values close to 0, shown in lighter colors, indicate no linear correlation between the species abundances.

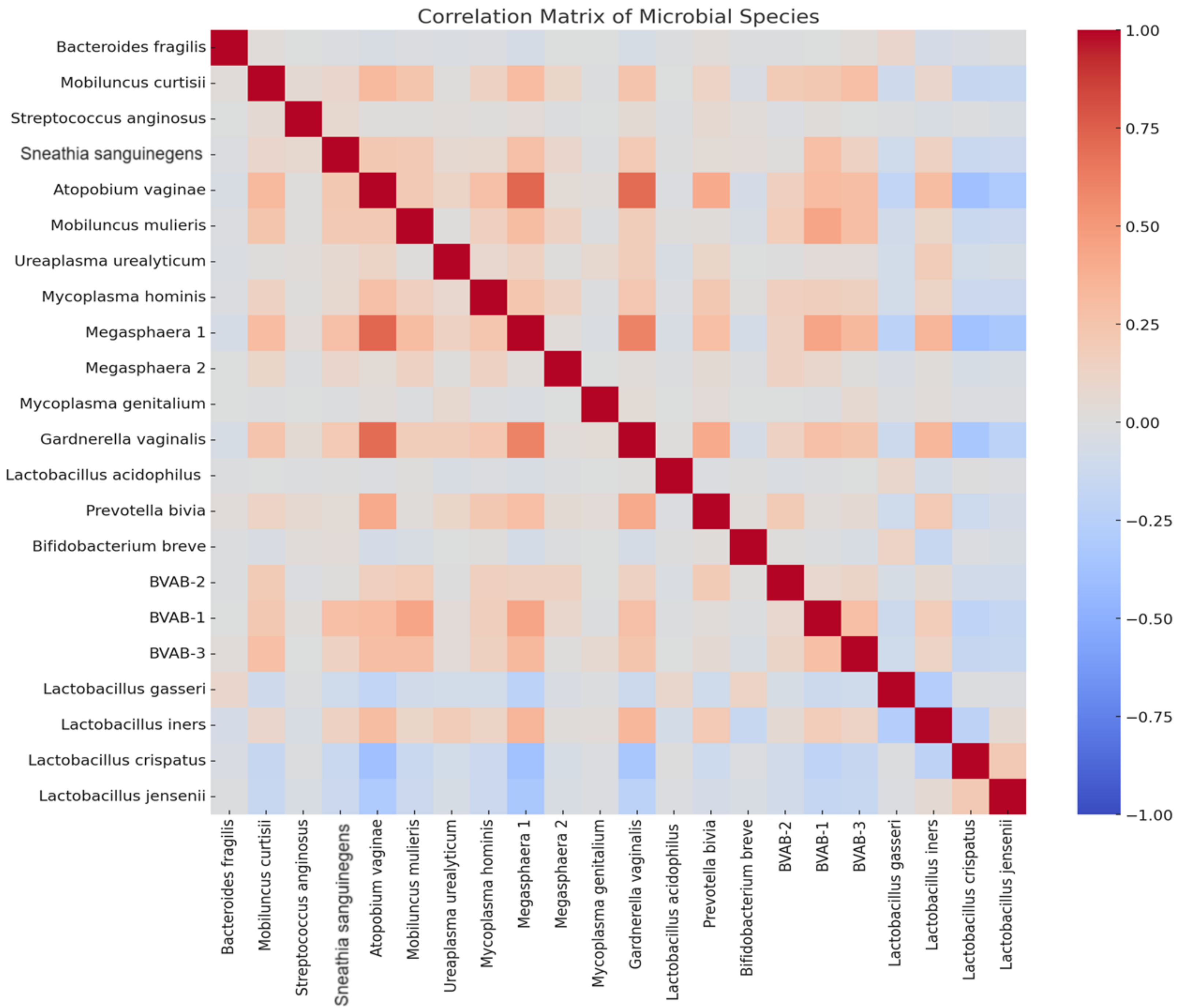

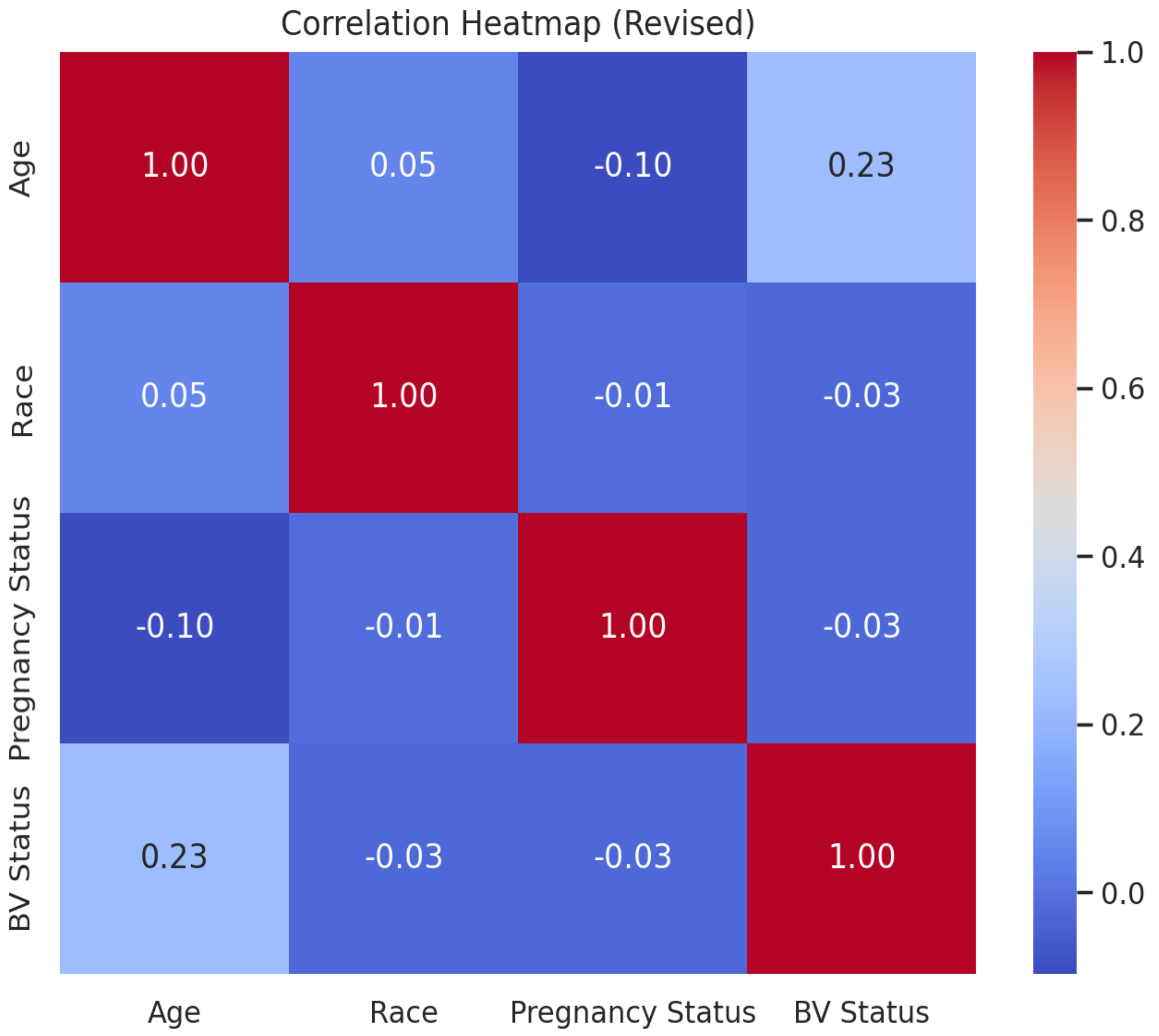

- **Figure S24. A heatmap showing the correlation between Age, Race, Pregnancy Status, and BV status.** Red boxes show strong positive correlation while blue ones show strong negative correlation. Of all these four variables, only age was positively correlated with BV status while pregnancy status and race were negatively correlated.

Fig. S25. A bar chart displaying the relative abundances of five *Lactobacillus* species in vaginal samples collected from pregnant and non-pregnant women. Except for *L. crispatus,* the mean relative abundance of the *Lactobacillus* sp. were generally a little higher in non-pregnant women than in pregnant women. Only *L. acidophilus* had a wider abundance variance between pregnant and non-pregnant women.

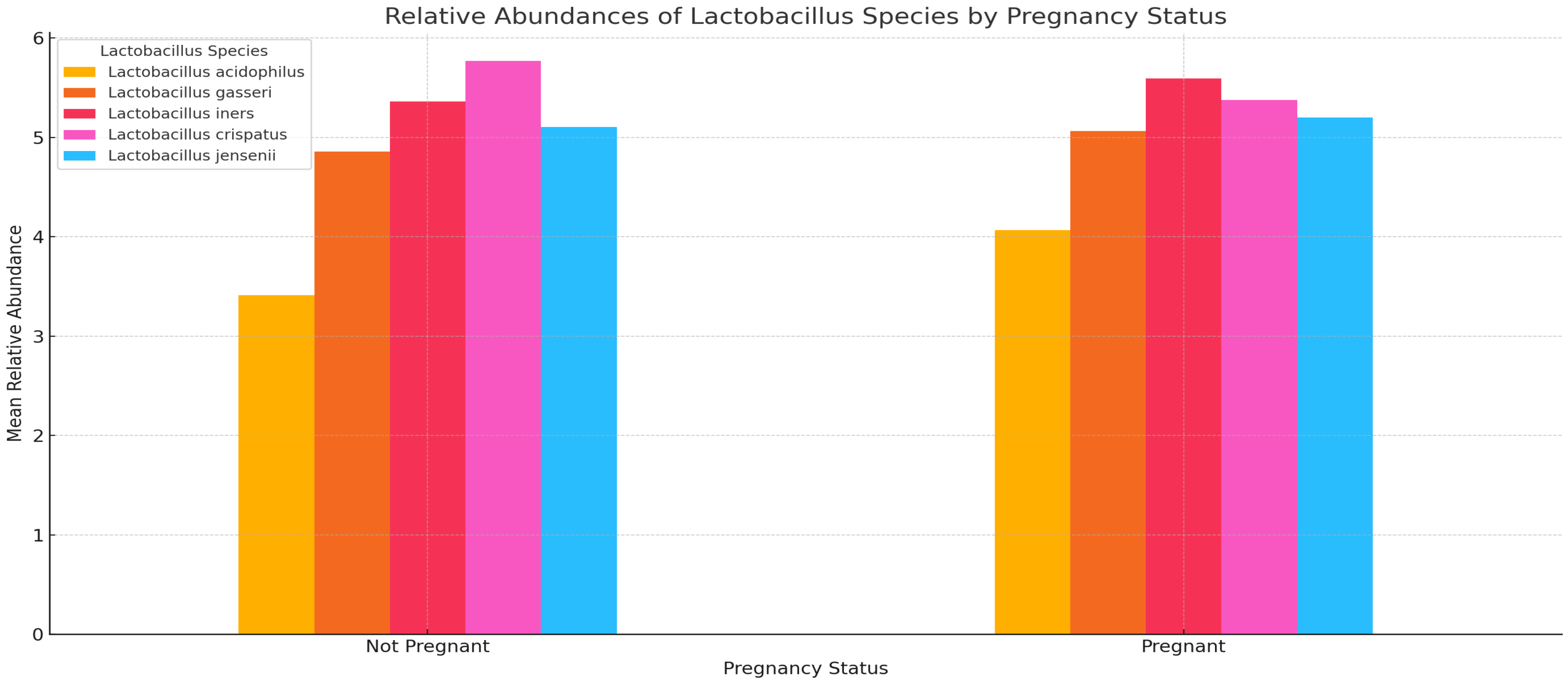

Fig. S26. A bar chart displaying the relative abundances of various non-Lactobacillus bacterial species across pregnancy statuses (Pregnant and Not Pregnant). Species such as *B. fragilis, Megasphaera* sp. type 2, and *B. breve* were only present in non-pregnant women. *M. curtisii, S. anginosus, U. urealyticum, M. himinis, Megasphaera* sp. type 1, *G. varginalis*, BVAB-2 and BVAB-1 had higher abundance in pregnant women; only *S. sanguinegens, F. vaginae, M. mulieris, P. bivia,* and BVAB-3 were higher in non-pregnant women.

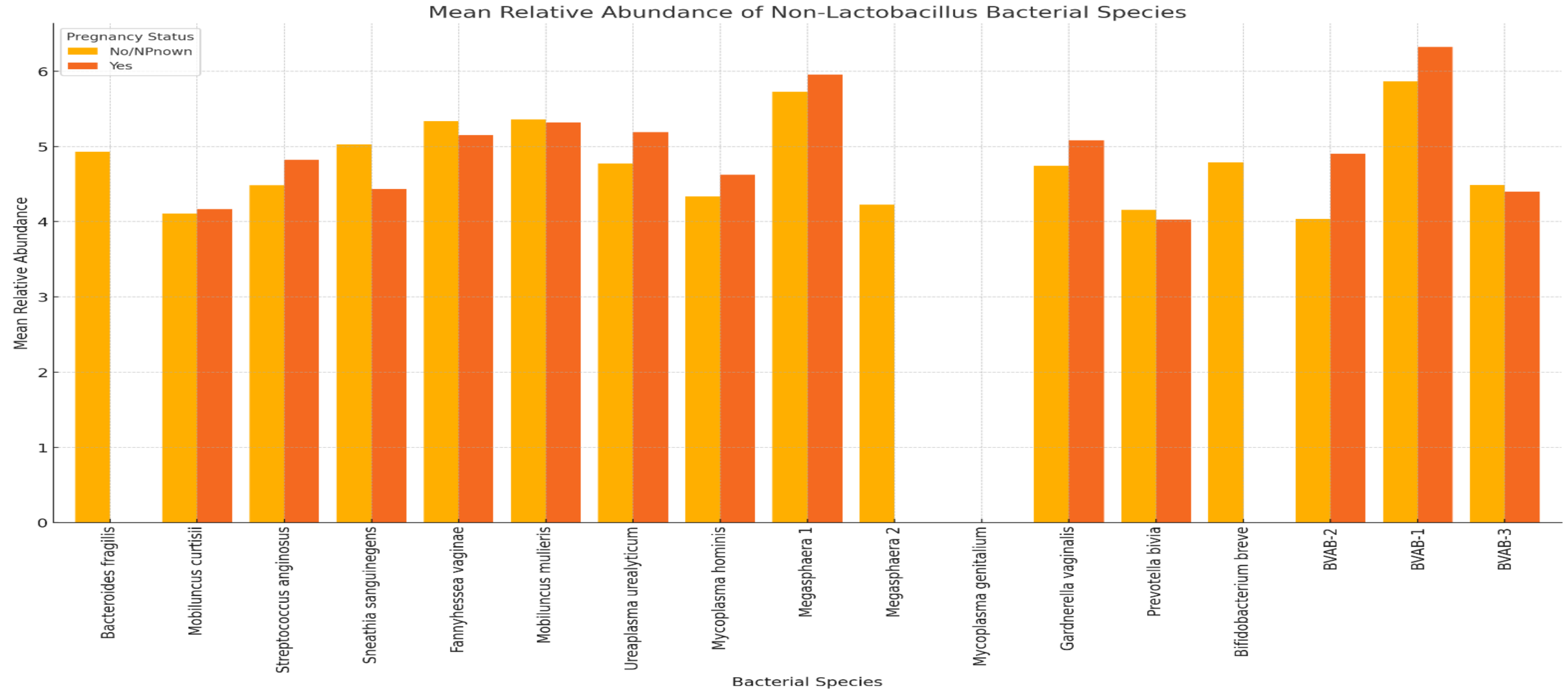

Fig. S27. Fig. A bar chart displaying the relative abundances of various non-Lactobacillus bacterial species across pregnancy statuses (Pregnant and Not Pregnant). Non-pregnant women had higher diversity (n = 16 species) and generally lower mean relative abundance than pregnant women (n = 13 species). Species such as *B. fragilis, Megasphaera* sp. type 2, and *B. breve* were only present in non-pregnant women. *M. curtisii, S. anginosus, U. urealyticum, M. himinis,* Megasphaera sp. type 1, *G. varginalis*, BVAB-2 and BVAB-1 had higher abundance in pregnant women; only *S. sanguinegens, F. vaginae, M. mulieris, P. bivia,* and BVAB-3

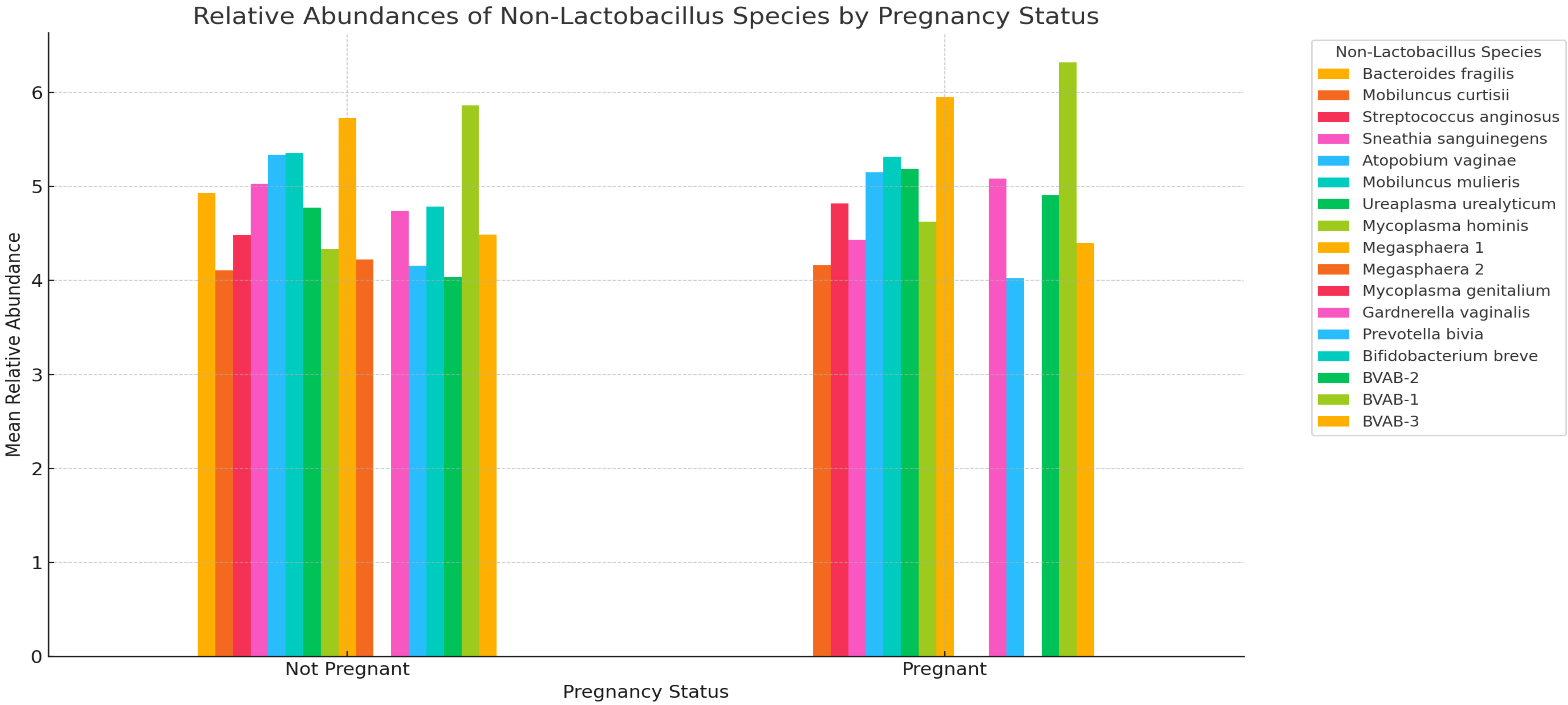

Fig. S28. A bar chart showing the mean relative abundance of the five *Lactobacillus species* within each age group. The different species had different abundances per age group, albeit some age groups had almost the same level of mean abundance. *L. acidophilus* was more abundant in age 41-50 group, followed by age 31-40 and 21-30 groups. *L. crispatus* was higher than the other species in all age groups except 0-20 group, where *L. iners* & *L. jensenii* were higher.

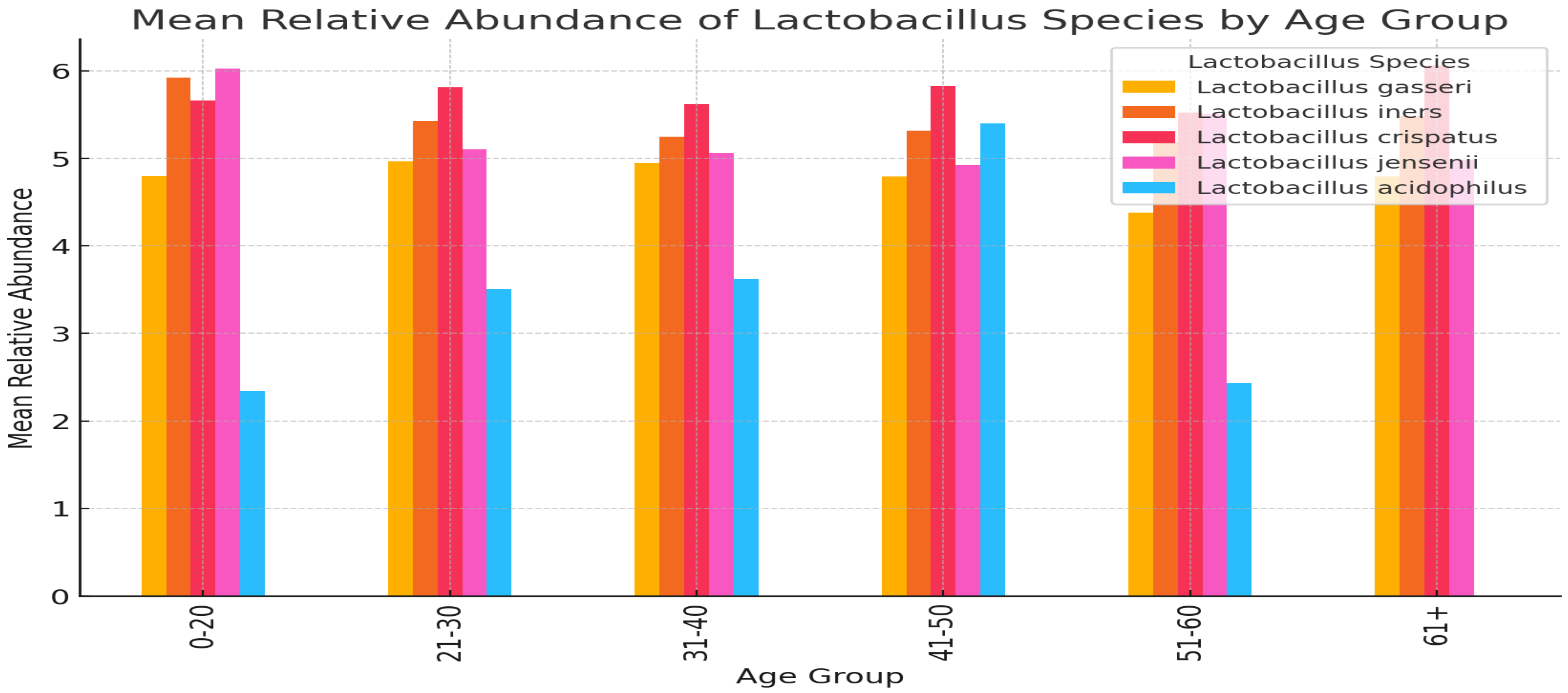

Fig. S29. A bar chart showing the mean relative abundance of the non-Lactobacillus species within different age groups. *B. fragilis, S. anginosus, and Megasphaera* sp. type 2 were absent from some age groups.

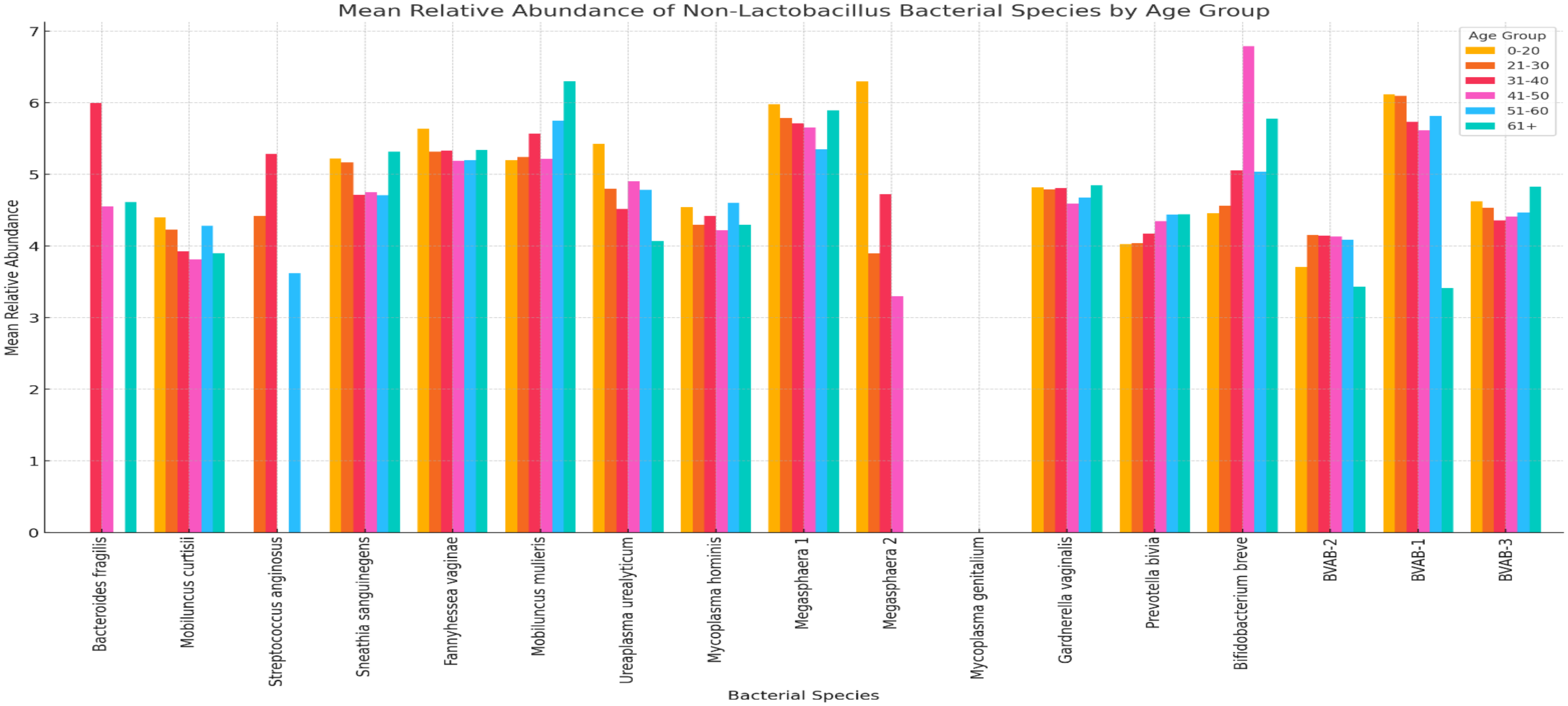

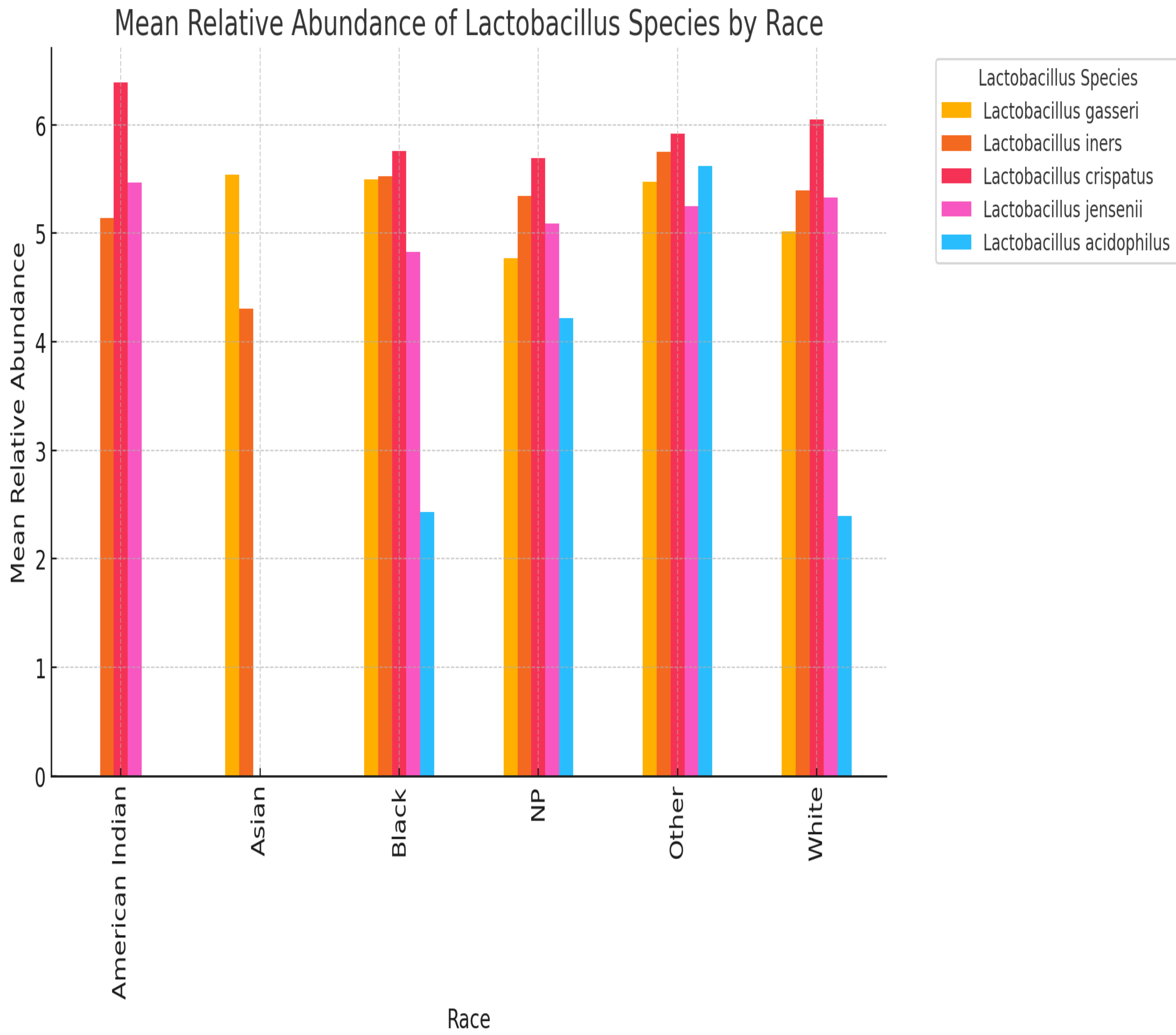

Fig. S30. A bar chart showing the mean relative abundance of Lactobacillus species within different races. White, Black, and other races had more diversity of *Lactobacillus sp*. while Asian people had very little diversity. Lactobacillus acidophilus was higher in other (Hispanics and Pacific Islanders) races than White and Black people. *L. crispatus* was higher in American Indians and White people while *L. gasseri* & *L. iners* was higher in Black and Other races. *L. gasseri* was also higher in Asian people.

Fig. S31. Relative abundance distribution of Lactobacillus species in different racial/ethnic groups. The different races had variations in the relative abundance of Lactobacillus sp. as can be seen in the varying heights of the Box and Whiskers. *L. iners, L. crispatus,* and *L. jensenii* had a broader diversity distribution among most the races than the rest. NP means not provided. The Whisker show the upper and lower quartiles while the boxes show the 25th and 75th percentile range (50% of the population) while the stars show outliers.

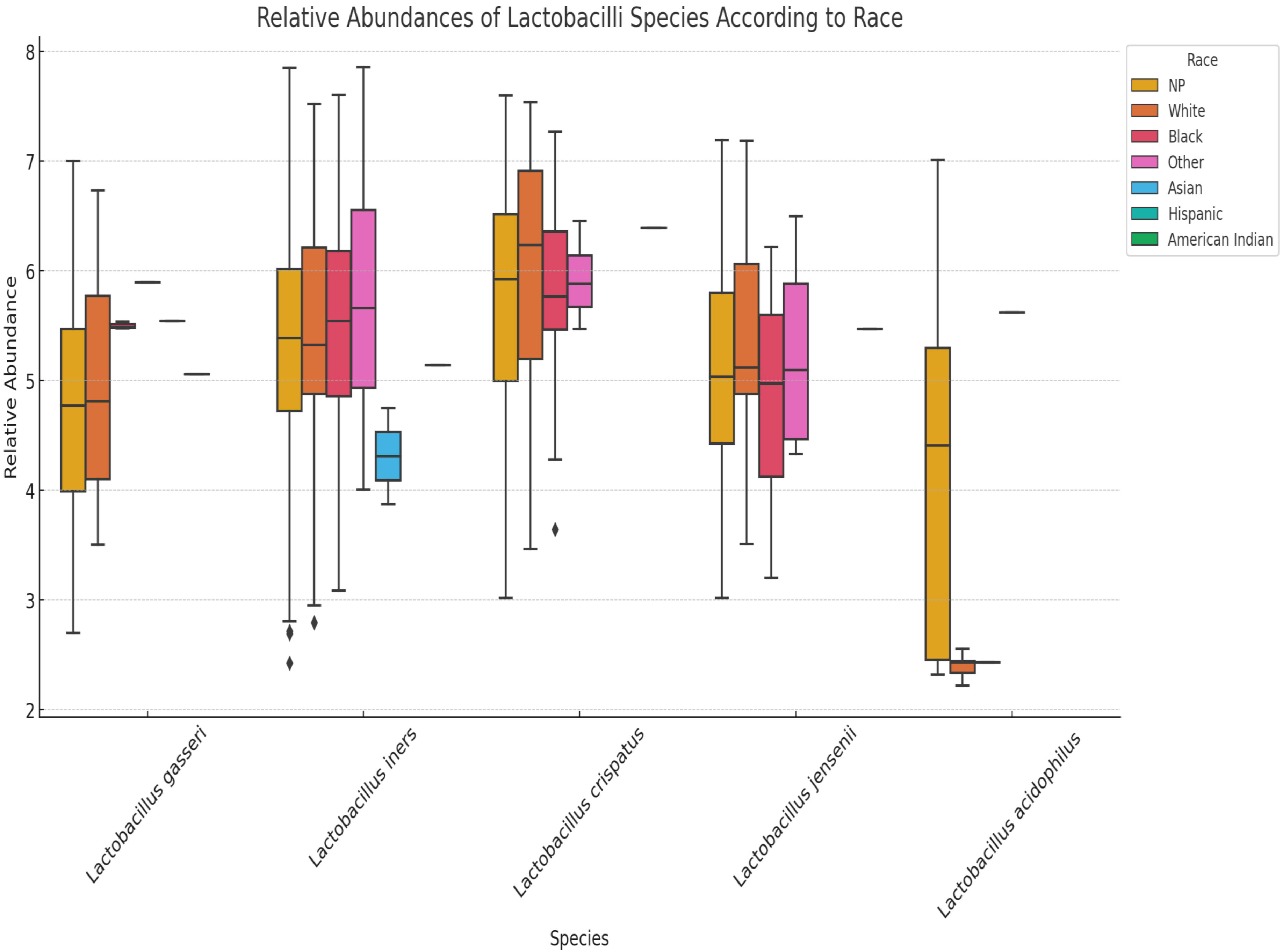

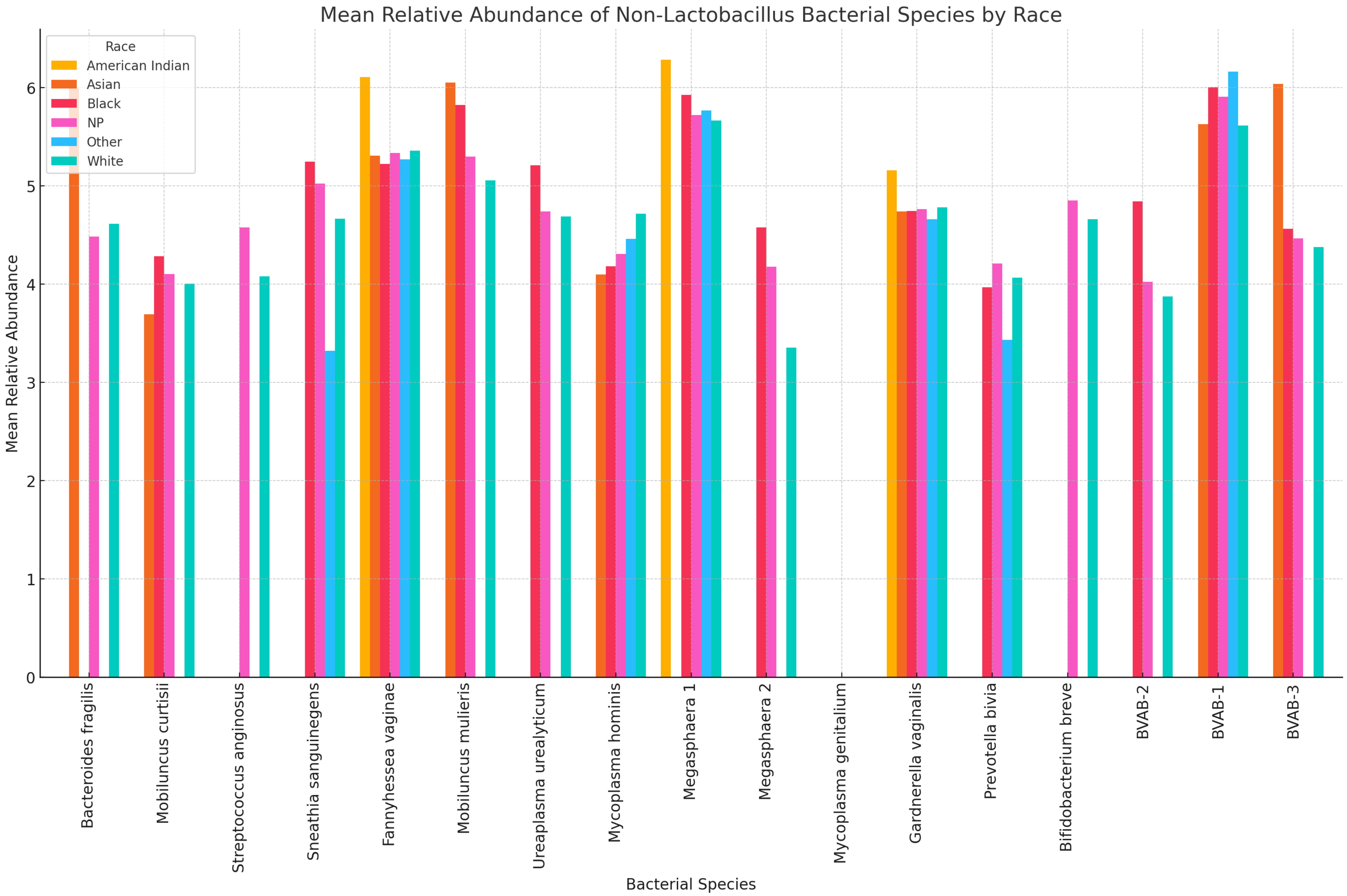

Fig. S32. A bar chart displaying the relative abundance distribution of non-*Lactobacillus* species in different racial/ethnic groups. For many of the species, there were a higher relative abundance among Black people than White people, explaining the higher prevalence of BV in Black people than White people. NP means not provided

Fig. S33. A Box and Whiskers plot showing the distribution of the non-*Lactobacillus* sp. in different racial groups.

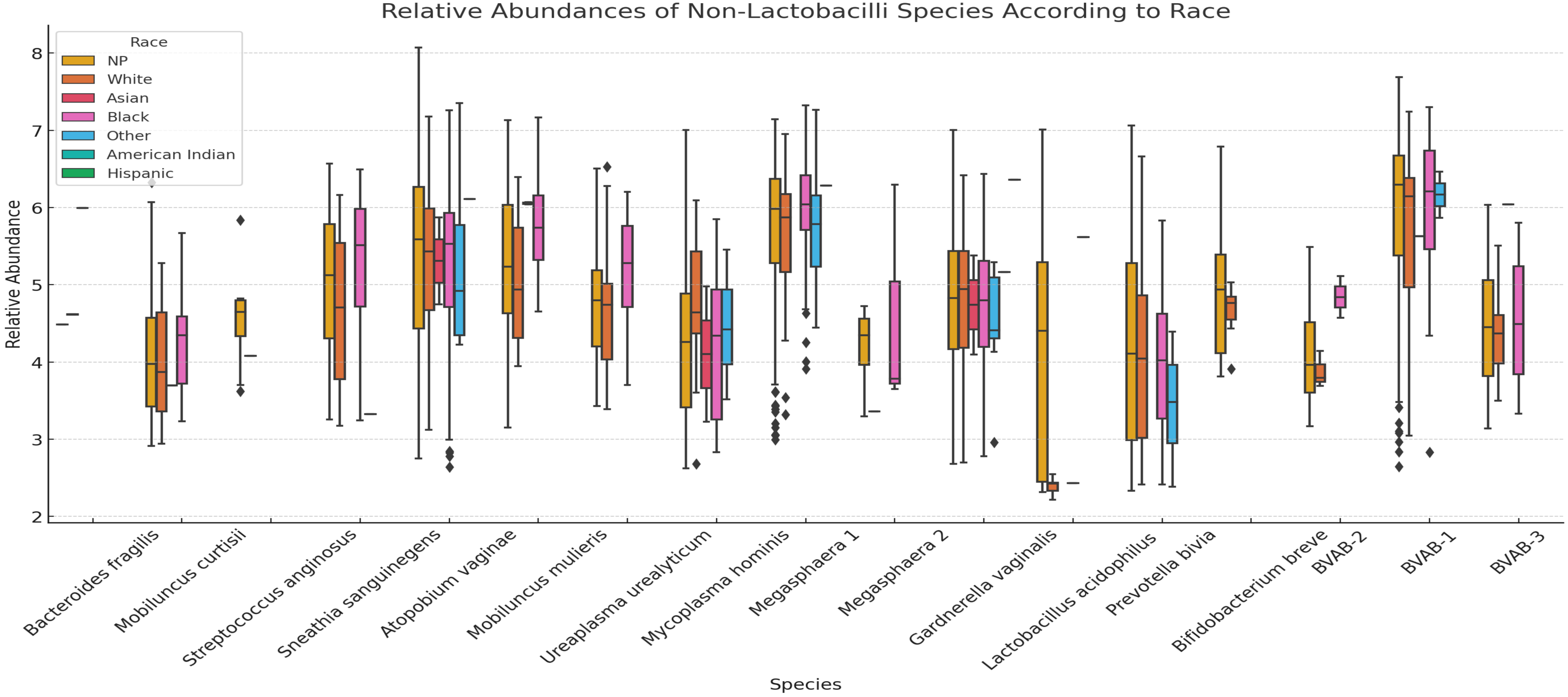

Supplement: Supplementary Figures 1–22 — Concentration distribution of each of the 22 bacterial species tested by the qPCR test across all the samples. Both boxplot and histograms charts are shown for each species. [file DataSheet_1.pdf]
